# Ethnic Accommodation and the Backlash From Dominant Groups

**DOI:** 10.1177/00220027251343836

**Published:** 2025-05-22

**Authors:** Andreas Juon

**Affiliations:** 1 Department of European Studies and Slavic Studies, University of Fribourg, Fribourg, Switzerland

**Keywords:** conflict resolution, peace agreement, separatism, internal armed conflict, conflict management

## Abstract

When does the accommodation of subordinate ethnic groups generate a backlash from the politically dominant group? I argue that power-sharing, regional autonomy, and multiculturalism lend themselves to the articulation of grievances and fears among members of dominant groups, especially if they explicitly recognize subordinate groups’ collective identities. In turn, nationalist parties can exploit such sentiment to organize protests, incite violence, and increase their electoral prospects. To test these arguments, I combine new monthly data on ethnic accommodation in 125 multi-ethnic electoral regimes between 1990 and 2018 with information on dominant group mobilization in anti-government protests and communal violence. I find systematic increases in dominant group mobilization around times when group-based accommodation is first introduced or expanded. These results enhance our understanding of mass mobilization by dominant ethnic groups. Moreover, they point to concrete proposals to reap the benefits of ethnic accommodation while avoiding a potentially destabilizing backlash against it.

## Introduction

How can ethnically-divided states reap the benefits of ethnic accommodation, while avoiding a backlash against it? The accommodation of subordinate groups—through power-sharing, autonomy, and multiculturalism—has demonstrated benefits for achieving peace and democracy in ethnically divided states. Yet such accommodation can trigger a potentially destabilizing backlash from the dominant group, in the form of large-scale anti-government protests or communal violence. Despite the risk that such a backlash poses for conflict resolution efforts, it has not been systematically investigated in existing cross-national research. Indeed, we know little about the factors that drive dominant groups’ mobilization more generally, as lamented by numerous observers (Basta 2021; Bonikowski 2016; Coakley 2011).

Investigating the conditions under which ethnic accommodation can generate a large-scale backlash from dominant groups is politically urgent. Following World War II, the international community has converged on the accommodation of subordinate ethnic groups—often corresponding to minorities—to safeguard peace around the world (McCulloch and McEvoy 2018; McGarry et al. 2008). Indeed, observers have noted the emergence of a global inclusive norm, especially since the end of the Cold War (Gurr 2002). Ominously, however, recent years have seen an accelerating global backlash against such accommodation (Norris and Inglehart 2019; Tamir 2019). In some countries, such as India, this backlash threatens foundational public goods, including democratic quality and liberal rights (Pevnick 2024); in others, like Macedonia or Rwanda, it has triggered waves of violence against subordinate groups (Gottlieb and Hislope 2017; Straus 2006); in yet others, it has brought reactionary far-right parties to power (Bustikova 2014, 2019), endangering political moderation in the long-term.

These observations delimit the questions I address in this article. First, the form and scale of a potential backlash against accommodation—in the form of anti-government protests and violence against subordinate groups—requires explanation. While it is not surprising that some members of dominant groups oppose accommodation, it is far from clear when such resistance takes the form of potentially destabilizing mass protests and violence, rather than remaining confined to less costly electoral participation. Second, it remains unclear what drives variation in the *degree* of such a backlash. This raises the urgent question of how to reap the benefits of ethnic accommodation, while avoiding such a risk.

In this article, I address these questions and shed light on the conditions under which ethnic accommodation sparks a large-scale backlash from dominant groups—and how this risk can be mitigated. I focus on constitutional concessions that provide subordinate ethnic groups with representation in government, regional autonomy, and recognition of their cultural practices. I argue that such concessions lend themselves to the articulation of grievances and fears among the dominant group. In turn, nationalist parties claiming to represent the dominant group can exploit such sentiment to organize protests and incite violence to increase their electoral prospects. I argue that the risk of a large-scale backlash is particularly pronounced under two conditions: first, where at least one dominant nationalist party competes for political office; second, if concessions explicitly recognize subordinate groups’ collective identities.

I systematically test these expectations in a quantitative analysis comprising all multiethnic electoral regimes around the world since the end of the Cold War. To set up my analysis, I combine new monthly data on concessions to subordinate groups with information on anti-government protests and targeted violence involving the politically dominant group. I find that frequency of such mobilization is substantially higher in 3-month windows before and after concessions that introduce or expand ethnic accommodation. This association is particularly pronounced if a dominant nationalist party competes for office and is driven by concessions that formally recognize subordinate groups’ identities.

My findings are remarkably robust and apply to both post-conflict and stable contexts. Sensitivity analyses indicate that confounders equally or twice as strong as actually observed ethnic protest are needed to render the results statistically non-significant. This indicates that my findings are unlikely due to strategic considerations whereby concessions are tailored with respect to anticipated mobilization. Moreover, they cannot be explained away by selection effects whereby both accommodation and a backlash are more likely in especially divided societies. Finally, I provide individual-level corroborating evidence for the intermediate steps in my hypothesized mechanisms.

## Previous Research

I start by introducing three concepts. First, by *dominant group*, I refer to the ethnic group in a polity that holds the predominant share of political power, by virtue of its demographic, military, or bureaucratic dominance (cf. Mylonas 2012). This often, but not always, coincides with *demographic* plurality groups. However, in some cases, it refers to numerical minorities that hold a disproportionate share of political power, for instance the Afrikaners in apartheid South Africa or Iraq’s Sunni community under Saddam Hussein’s rule. In contrast, by *subordinate groups*, I refer to all other, politically less influential groups.

Second, by *ethnic accommodation* I refer to two main accommodative strategies: first, institutions that provide subordinate groups with governmental representation and influence over the legislative process; and, second, institutions that award them political and cultural autonomy. These dimensions closely correspond to the horizontal and vertical types of power-sharing, which have been characterized as the *sine qua non* of accommodation (Cederman et al. 2013, 2015; Lijphart 2004). Moreover, they encompass the most widely-advocated accommodative institutions, including consociationalism, informal power-sharing, regional autonomy, and multiculturalism (McGarry et al. 2008).

Finally, by *dominant group mobilization*, I refer to the incidence of mass mobilization that involves members of dominant groups. This comprises both non-violent and violent forms of mobilization (McAdam and Tarrow 2000), most notably anti-government protests and targeted violence against subordinate groups. In some cases, such mobilization may be conducted explicitly in the name of the dominant group. An example are Hindu nationalist protests and anti-Muslim riots in India, aiming to constitutionally enshrine Hindu dominance, abolish Kashmir’s autonomy, and limit Muslims’ cultural rights (Girvin 2020). In other cases, such mobilization may promote the interests of the dominant group, but be expressed in the language of individual equality. For instance, Bosniak nationalists have rallied against Bosnia’s consociational system by referring to the limitations its ethnic quota system places on individual equality (Basta 2016).

In spite of its demonstrated potential to prevent or unravel ethnic accommodation, dominant group mobilization has not figured prominently in the study of ethnic conflict. Existing research overwhelmingly focuses on the mobilization of subordinate groups, often corresponding to demographic minorities. In contrast, dominant groups, often corresponding to majority communities, are largely left off the radar (Basta 2021; Bonikowski 2016; Coakley 2011). In this vein, previous work highlights governmental efforts to preserve reputation vis-à-vis potentially conflict-prone subordinate groups, constraining its ability to offer accommodation (Roessler and Ohls 2018; Walter 2009). In contrast, the potentially equally destabilizing reaction by dominant groups has rarely been examined.

Of course there is important work that examines outcomes related to the mobilization of dominant groups. An influential literature highlights that nationalist or opportunistic elites claiming to represent the dominant group may incite riots for instrumental reasons, in order to politicize ethnic cleavages, intimidate voters of subordinate communities, and gain electoral advantages (Brass 1991; Müller-Crepon 2022). Some studies point to an important role of ethnic accommodation in these processes, including in Serbia (Hislope 1997), Sri Lanka (Horowitz 1985), and India (Wilkinson 2000), highlighting that radical nationalist flanks may exploit resentment against accommodation to incite riots. This underlines the need to examine when accommodation lends itself to such mobilization, including in less prominently-discussed cases and for nonviolent forms of mobilization.

A nascent literature illuminates the processes that can spark a backlash against ethnic accommodation in specific cases or regions. Bustikova (2014, 2019) shows that extreme right parties in Eastern Europe were more successful following the government inclusion of pro-minority parties. Basta (2021) traces back mobilization by dominant groups against federal arrangements in Canada, Spain, and Yugoslavia to their recognition of subordinate groups’ nationhood. These studies highlight the potential of accommodation to generate resentment among members of dominant groups—and spark mass mobilization—in specific cases. This underlines the importance of examining whether these findings form part of a generalizable pattern.

Finally, a prominent literature examines assertions that ethnic accommodation may boost the vote shares of nationalist and right-wing populist parties, by generating grievances and fears over the dominant group’s diminished political, economic, and cultural status (Bonikowski 2016; Norris and Inglehart 2019; Pevnick 2024). Mainly focusing on Western Europe and North America, this literature shows that nationalist and populist parties may politicize even limited forms of accommodation geared towards recent immigrants to gain electoral advantages. This highlights the need to consider the consequences of the—often far more extensive—accommodation of long-resident groups, including in other world regions.

In sum, extant research focuses on the mobilization of politically subordinate groups and governing elites. In contrast, we know much less about the conditions under which members of dominant groups are receptive to appeals to mobilize against ethnic accommodation and how this risk can be mitigated. In the rest of this article, I address these gaps.

## Theory

### Grievances and Fears as Drivers of Dominant Group Mobilization

My theoretical framework is centered on the perceptions and actions of ordinary members belonging to the politically-dominant ethnic community. I conceive of these individuals as boundedly rational actors whose political behavior is critically influenced by their emotions, personal value orientations, societal norms, and the framing of political issues by elites (cf. Mintz et al. 2022).

Departing from this general conception, I expect ethnic accommodation to facilitate the mobilization of dominant groups through two attitudinal channels. First, accommodation may generate grievances among members of dominant groups, defined as assessments that they are subject to unjust treatment. Such assessments become more likely, as the mismatch increases “between reality” and their “conception of a ‘just’ hierarchy” (Petersen 2002, 43). Critically, for many members of dominant groups, this conception may be radically different than for subordinate groups, who may be content with sharing political power and achieving recognition of cultural parity (Cederman et al. 2013). In particular, with their own group’s representation assured under the status quo, members of dominant groups may oppose concessions on the grounds that they violate the principle of individual equality. Moreover, conservative members of dominant groups with a strong sense of national identification may even feel entitled to political and cultural *pre-eminence* and oppose the representation and recognition of subordinate groups across the board (Horowitz 1985, chapter 5).

Accordingly, ethnic accommodation can violate dominant groups’ conception of a just status hierarchy in several ways. Most obviously, members of dominant groups that are at the same time *demographic* pluralities may perceive ethnic accommodation as a violation of the democratic norms of individual equality and majority rule (Lijphart 1996, 262; McCulloch 2014, 511). For instance, this was the case in Cyprus, where power-sharing was “resented” by some Greeks “for giving disproportionate powers to Turks” (Ghai 2002, 146). However, irrespective of their group’s demographic size, members of dominant groups may also perceive ethnic accommodation as unjust where it constitutes a fundamental break with enduring periods of political dominance (Cederman et al. 2013, 182). For in 2004, Sunni protests erupted against the drafting of a new power-sharing constitution that abolished their long-standing privileges as the ruling group (Bogaards 2019).

Second, accommodation may generate fears, especially among members of dominant groups that hold authoritarian value or social dominance orientations, who have strong preferences for conformity, security, and hierarchically-structured intergroup relations (Sidanius et al. 2017). In some cases, such individuals may perceive ethnic accommodation as a threat to their state’s national identity and its territorial integrity (Basta 2021). One example is North Macedonia, where the 2001 Ohrid agreement recognized the Albanian language and raised prospects for decentralization reforms. This reinforced pre-existing fears of a Greater Albanian project that might split up the state, which nationalists exploited to organize protests and riots (Brunnbauer 2002). In other cases, especially if they are demographic minorities, members of dominant groups might fear that accommodation will enable formerly excluded, resentful outgroups to take revenge, as is more likely following status reversals (Bulutgil 2016; Petersen 2002). An example was South Africa in the final stages of apartheid, when a large proportion of formerly-dominant Afrikaners feared that, under a potential power-sharing deal, they might be discriminated against, their living standards might suffer, and their physical safety would decline (Clark and Worger 2011, 110).

More widespread grievances and fears among dominant groups do not necessarily translate into more frequent anti-government protests or violence against subordinate groups. However, they facilitate the task of dominant group elites to mobilize followers for either purpose. Tapping into grievances and fears, they can formulate well-resonating injustice or fear frames (Benford and Snow 2000; Gamson 2013). Such frames enable them to create consensus around both an asserted problem—ethnic accommodation—and its solution—policies that abolish privileges for subordinate groups or even explicitly protect the dominant community’s status. As shown by work that focuses on subordinate groups, perceptions of unfair treatment or impending threat increase the plausibility of claims that the political system needs to be reformed or even overthrown, if need be with pressure from the street or violence (Cederman et al. 2013; Germann and Sambanis 2021). I expect similar dynamics to apply to the mass mobilization of dominant groups as well.

Following the above argumentation, ethnic accommodation should be associated with a persistently larger reservoir of individuals that can be mobilized. However, elites’ ability to do so should be especially pronounced right before proposals for accommodation are introduced or after they have been adopted. Such periods are windows of opportunity during which elites can employ urgency and existential threat frames, which call for immediate action to avoid an imminent loss of political privilege or evade an impending threat (cf. Desrosiers 2012). Moreover, concessions that restrict their dominance can trigger loss perceptions, which entail particularly intense emotional reactions. Similar to other individuals, members of dominant groups are likely to see recent losses more keenly as disutility than gains as utility (Kahneman and Tversky 1979). Thereby, the perception of impending loss offers elites the opportunity to generate a disproportionate amount of collective action.

I summarize these arguments in a first hypothesis:

Hypothesis 1 Dominant group mobilization is more frequent immediately before and after concessions that accommodate subordinate groups.

### The Moderating Role of Inter-Elite Competition

Why would elites of dominant groups organize mass protests and incite communal violence? While elites representing dominant groups by definition hold influential government positions, they are often subdivided into distinct organizations and factions that are engaged in nested competition with one another (Horowitz 1985, 353; Hislope 1997). Following previous research (Brass 1991; Müller-Crepon 2022; Vogt et al. 2021; Wilkinson 2000), I expect ethnic accommodation to generate or accentuate outbidding dynamics between rival elites seeking political support from the dominant community. Competing elites will use ethnic accommodation to organize protests and violence in order to increase their visibility, sharpen their ideological profile, and improve their electoral prospects.

Based on this reasoning, I expect dominant group elites’ incentives to organize mass mobilization to increase with the degree of competition between them. Of particular importance are nationalist parties that claim to represent the dominant group. Such *dominant nationalist* parties’ electoral platforms are based on the affirmation of the dominant group’s pre-eminence and the negation of subordinate groups’ political and cultural rights. Dominant nationalists often compete for support from conservative and authoritarian members of the dominant group, who are most receptive to claims that ethnic accommodation violates their group’s entitlements or constitutes a serious threat to the established status hierarchy. Organizing protests and inciting violence allows them to distinguish themselves from electoral rivals and portray themselves as the most committed defenders of the dominant community (cf. Vogt et al. 2021).

Three examples illustrate this logic. A first is the abuse of Indian Muslims’ cultural rights and Kashmir’s autonomy as wedge issues by Hindu nationalist parties, such as the Bharatiya Janata Party and Shiv Sena (Girvin 2020, 790; Wilkinson 2000). A second example is North Macedonia, where the appointment of ethnic Albanian ministers was exploited by nationalist parties to organize widespread protests and anti-Albanian riots in 2001, 2013, and 2017 (Clark and Regan 2016), allowing them to boost their vote share (Bustikova 2014, 1741-2). A final example is Sri Lanka’s 1956 Bandaranaike-Chelvanayakam Pact. Thereby, the ruling Sri Lanka Freedom Party, itself ardently Sinhala nationalist, sought to accommodate the Tamil minority with cultural autonomy. Exploiting discontent among Sinhala extremists and the conservative Buddhist electorate, the opposition United National Party rallied against these largely symbolic measures, paving the way to its 1960 electoral victory (Hennayake 1992; Horowitz 1985).

I summarize the observable implications of these arguments in a second hypothesis:

Hypothesis 2 The frequency of dominant group mobilization before and after concessions increases if at least one nationalist party claiming to represent the dominant group competes in national elections.

### Group-Based Versus Formally Group-Blind Forms of Accommodation

So far, I have argued that ethnic accommodation, whichever its precise form, enables dominant group elites to articulate grievances and fears, which resonate among members of dominant groups, especially those that are politically conservative and hold social dominance or authoritarian value orientations. In this section, I argue that their ability to do so critically depends on the institutional form of accommodation.

I distinguish between two fundamentally different ways of providing for ethnic accommodation. First, by *group-based* accommodation, I refer to constitutional provisions that explicitly recognize subordinate groups’ collective identities. This characterizes ethnically based power-sharing institutions, which provide for subordinate groups’ representation in government through group-based quotas and veto rights (McCulloch 2014; McGarry et al. 2008). It also characterizes constitutional provisions that designate administrative regions as specific groups’ national homelands or that explicitly recognize their cultural customs (Basta 2021; McGarry et al. 2008). Prototypical examples thereof are Bosnia’s 1995 and Burundi’s 2004 constitutions, which introduced comprehensive quota and veto systems benefiting Serb, Croats, and Tutsi minorities, respectively. Another is Montenegro’s 2007 constitution, which formally recognized Serbs, Bosniaks, Albanians, Muslims, and Croats as national minorities, provided for the official use of their languages and scripts, and acknowledged their rights to set up their own educational, cultural, and religious associations.

Conversely, by formally *group-blind* accommodation, I refer to policies and institutions which protect subordinate groups’ interests without formally recognizing their collective identities. This characterizes informal power-sharing pacts that are not constitutionally mandated and non-ethnic power-sharing institutions, such as proportional electoral systems and supermajority requirements (McCulloch 2014; McGarry et al. 2008). It also characterizes decentralization reforms that delegate policy and fiscal competencies to diverse regions, without formally recognizing subordinate groups’ nationhood (Basta 2021). Prominent examples include South Africa’s 1994 interim constitution, which gave all parties above a 5 percent vote share the right to cabinet representation, and Iraq’s 2004–2005 federalization reforms, which uniformly expanded the competencies of all pre-existing governorates (McGarry et al. 2008).

Building on my hypothesized mechanisms, I expect group-based concessions to generate a disproportionate amount of dominant group mobilization. First, they increase the plausibility of injustice and fear frames. As their ethnically differentiated institutions ostensibly deviate from pure majority rule, dominant group elites can cite concrete constitutional provisions as evidence for claims that accommodation violates the principle of individual equality or even that the dominant community is unjustly treated. Moreover, by recognizing *multiple* ethnic communities, group-based concessions may also visibly clash with conservative citizens’ monist political view of the state (Basta 2021, 46), making them more amenable to claims that accommodation constitutes a threat to their national project. For instance, as Basta (2021) demonstrates, Serb resistance to Yugoslavia’s 1974 federal constitution centered on the explicit “principle of multinationalism” that the constitution established, which nationalists and opportunistic former Communist elites could frame as “advantageous to all but the Serbs” (Basta 2021, 134).

Second, group-based concessions also incisively circumscribe the responsiveness of the political system to the dominant group’s preferences. Their constitutionally-entrenched, explicitly ethnic guarantees raise the expectation that these restrictions will persist over time. Indeed, this is one of the main reasons why subordinate groups tend to demand formal, group-based guarantees in the first place (Bogaards 2019; Lijphart 1995; McCulloch 2014). Yet, this greatly raises the stakes of constitutional negotiations. When group-based concessions are first proposed, their rigidity and expected time persistence enables dominant group elites to employ gravity and urgency frames to “create the sense that windows of opportunity are closing, that it is ‘now or never’” (Desrosiers 2012, 15). Hence, they will find it easier to convince conservative and authoritarian group members of engaging in potentially costly mobilization to ward off difficult-to-reverse concessions.

Of course, formally group-blind concessions can also provoke counter-mobilization, as is evident by the examples of North Macedonia (Brunnbauer 2002; Gottlieb and Hislope 2017) and South Africa (Clark and Worger 2011) described above. However, through their cognitively accessible, symbolically salient, and rigid restrictions on the dominant group’s dominance, group-based concessions lend themselves more readily to the articulation of grievances and fears among conservative and authoritarian members of the dominant community. Thereby, they enable dominant nationalist parties to organize a disproportionate amount of collective action. I summarize these arguments in a third and final hypothesis:

Hypothesis 3 Dominant group mobilization is more frequent before and after group-based concessions, as compared to formally group-blind concessions.T

## Data

### Sample and Unit of Analysis

I test these expectations in a quantitative analysis that covers all multi-ethnic, electorally competitive countries between 1990 and 2018. I identify all countries with at least two politically relevant ethnic groups according to the Ethnic Power Relations Dataset (EPR) (Vogt et al. 2015). My argument focuses on regimes with at least a minimal level of electoral competition where elites have incentives to mobilize their membership to boost their electoral support. Hence, I only select country years that V-Dem’s *Regimes of the World* classification considers an “electoral autocracy”, an “electoral democracy”, or a “liberal democracy”, excluding “closed autocracies”. While established electoral regimes most closely fit my argument, similar dynamics apply to democratizing states, in which elites expect having to compete in upcoming elections. Hence, I additionally include years of ongoing democratic transitions, according to V-Dem Episodes of Regime Transition (Maerz et al. 2021). The resulting sample comprises a total of 125 multiethnic countries during the years in which they satisfy these minimal democratic criteria between 1990 and 2018. In appendix 3.3, I show that my results do not hinge on the inclusion of electoral autocracies and democratizing states.

I have argued that a backlash is most likely when accommodation is first introduced or expanded. Hence, I opt for a temporally fine-grained unit of analysis, focusing on the country month. I compile a monthly list of ethnic groups and their political influence for all countries in my sample, building on the yearly list of ethnic groups and their power access at the start of each year provided by the Ethnic Power Relations Dataset (Vogt et al. 2015). To transform this into a monthly format, I identify cut-off points at which the relative power of groups in government is subject to change, including national elections, successful coups, civil war termination years, and constitutional amendments. I extrapolate the list of groups and their power status in-between such cut-off points to identify the situation at the start of each month. I identify the dominant group as the group with the highest level of government influence at the start of each month, analogously to Bormann et al. (2017). Where multiple groups jointly hold the highest power status (e.g., Yugoslavia before 1992), I select the demographically largest one.

### New Monthly Data on Concessions to Subordinate Groups

Next, I operationalize concessions that provide for ethnic accommodation. In line with my theoretical framework, I consider both horizontal concessions—which increase subordinate groups’ representation in the central government—and vertical ones—which provide them with political and cultural autonomy. To test hypothesis 3, I further distinguish between group-based and formally group-blind concessions within both dimensions (see Table 1).

**Table 1.** Ethnic Accommodation: Types of Concessions Covered.

	Group-Based	Group-Blind
Horizontal	Ethnic quotas for executive or legislature; ethnic veto rights over executive selection; ethnic veto rights over legislation + const. amendments	Informal representation of subordinate groups in executive; proportional representation of elected parties in executive; proportionality of electoral system; supermajority requirements for executive election; supermajority requirements for legislation + const. amendments
Vertical	Designation of regions as subordinate groups’ national homelands; recognition of subordinate groups’ ethno- cultural identity, organizations, customs, land rights, education/media/judiciary, nongovernmental organizations, cultural symbols and festivals	Regional competencies over economy, culture, education, welfare, military, police, judiciary, institutions, citizenship, secession, residual powers; taxing and borrowing powers; ind. legislature and executive

Sources include the Ethnic Power Relations Dataset (Vogt et al. 2015), the Constitutional Power-Sharing Dataset (Juon 2023), and additional coding by the author, based on constitutions and autonomy statutes (see data supplement S1 for details).

To identify horizontal concessions, I combine information on constitutional power-sharing from the Constitutional Power-Sharing Dataset (CPSD) (Juon 2023) and de-facto power-sharing from EPR (Vogt et al. 2015). My measurement of group-based horizontal concessions captures constitutional provisions that mandate the representation of specific groups. Conversely, their group-blind alternative encompasses constitutional provisions that mandate power-sharing indirectly through electoral provisions and informal arrangements whereby subordinate groups become represented in the executive without constitutional stipulation.^1^

To identify vertical concessions, I screen all constitutions and autonomy statutes that were operational in my sample. I identify group-based vertical concessions in months where constitutional provisions where introduced or expanded that recognize subordinate groups’ cultural practices. I identify their group-blind alternative in months where institutions were introduced or expanded which provide for autonomous self-rule in minority-inhabited regions. In data supplement S1, I provide details on the coding of both horizontal and vertical concessions, along with a full list of all identified concessions.

**Figure 1.**
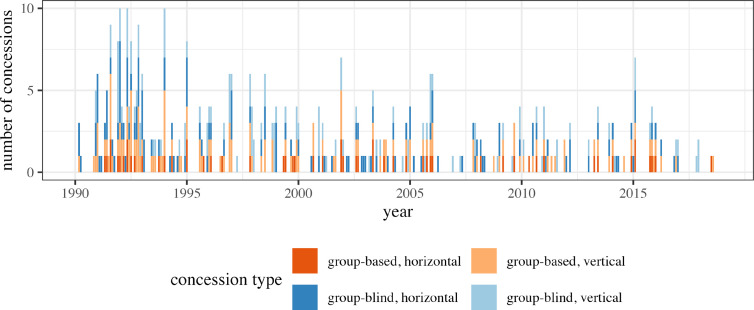
Ethnic accommodation: monthly concessions to subordinate groups around the world, 1990–2018. For a full list of concessions, see data supplement S1.

In my sample of 125 countries, I identify a total of 442 concessions between 1990 and 2018. In line with observations of an emerging inclusive norm (Gurr 2002; Jenne 2007; McCulloch and McEvoy 2018; Wimmer 2015), a descriptive overview (see Figure 1) suggests that multiethnic states have made consistent use of concessions to accommodate subordinate groups throughout the post-Cold War period, though their frequency has declined markedly since the early 1990s. In this period, governments were somewhat more likely to make group-blind concessions (252 concession months in total) than group-based concessions (190).

Using this fine-grained information, I construct three independent variables. My first variable, *concession number*, with an empirical range from 0 to 6, counts the number of concessions awarded to subordinate groups in the 3 months before and after each given month.^2^ This variable enables me to test hypotheses 1 and 2, which posit a higher frequency of dominant group mobilization in time periods around concessions. My second and third variables—*concession number (group-based)* and *concession number (group-blind)*—repeat this procedure, but exclusively consider group-based and formally group-blind concessions, respectively. Together, these variables enable me to test hypothesis 3, which posits a higher frequency of dominant group mobilization in response to group-based concessions. An important limitation of all three indicators is that they do not count concessions that were almost introduced, but discarded, for instance owing to mounting majoritarian protests. This means that I am likely to underestimate the effect of concessions. In my robustness checks (appendices 2 and 3), I explore numerous alternative operationalizations of these variables.

### Data on Dominant Group Mobilization

Next, I operationalize my main dependent variable, the monthly number of dominant group mobilization events. While several datasets exist that measure the mobilization of subordinate groups (Germann and Sambanis 2021; Vogt et al. 2015), I am not aware of any dataset with corresponding information on counter-mobilization by dominant groups. I hence rely on a new effort to systematically identify dominant group mobilization in the post-Cold War period (1990–2018).

To identity anti-government protests, I rely on information provided by the Mass Mobilization Data Project (MMD) (Clark and Regan 2016). This covers anti-government protests around the world since 1990. To identify protests involving dominant group members, I use information on protester identity provided by MMD. I code a protest event as linked to a specific group, if MMD’s news extracts mention that it was organized by members of this group or if organizers made explicit claims on its behalf. I consider any protest that is either explicitly linked to the group that I identified as the dominant group in a given month (see above) or that does not feature links to any ethnic group (and by extension, likely includes members of the dominant community in the protesters’ ranks).

To operationalize targeted violence against subordinate groups, I rely on information from the Uppsala Conflict Data Program (UCDP) dataset (Sundberg et al. 2012). This provides information on non-state conflicts—such as ethnic riots and communal violence—that resulted in at least 25 battle-related deaths in at least 1 year. To identify violence perpetrated by dominant groups, I attribute the participants on both sides of each nonstate conflict (Pettersson et al. 2019; Sundberg et al. 2012) to the respective ethnic groups by name. In data supplement S2, I provide more information on the ethnic attribution of both anti-government protests and targeted violence.

It bears emphasizing that my measurement of dominant group mobilization, as described above, primarily relies on the ethnic identity of the actors, rather than on public claims of their political intent. This is for two reasons: First, such information is difficult to obtain and reliably code; indeed, it was not systematically available for either of my underlying datasets. Second, participants in a substantial share of protest and communal conflict events, especially those initiated by informally organized groups, rarely make such public statements in the first place. Indeed, as highlighted in my argument, opposition to concessions is often publicly expressed in the language of individual equality. Hence, I preferred to err on the side of inclusivity and adopted an encompassing coding that is based on actor identity. However, in appendix 2.2, I show that my results remain robust when using a more narrowly coded dependent variable that only incorporates protests that the available information from MMD links to questions of institutional design and minority rights.

**Figure 2.**
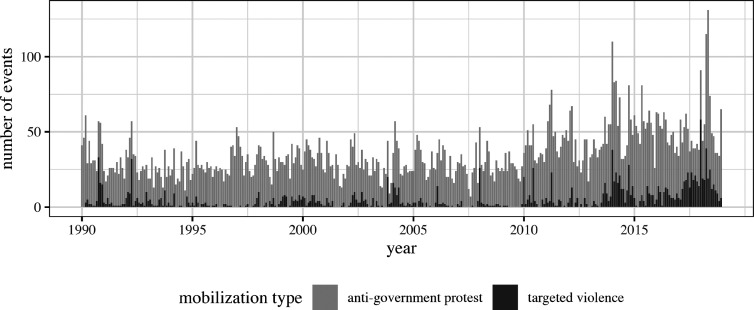
Monthly anti-government protests and targeted violence involving dominant group members around the world, 1990–2018.

Using both sources, I construct my main dependent variable, the *number of monthly dominant group mobilization events*. This counts the total number of events—anti-government protests and targeted violence—that involve members of the dominant group in each month. In my sample, I identify a total of 12,371 mobilization events between 1990 and 2018, with the vast majority (86 percent) thereof being protests (see Figure 2). Echoing warnings of a mounting backlash against ethnic accommodation (Norris and Inglehart 2019; Tamir 2019), my data show a trend towards a higher frequency of dominant group mobilization. Worryingly, this includes a pronounced increase of violence directed against ethnic minorities.

### Control Variables

I include a number of control variables across my models. A first set of controls captures strategic actions of dominant group elites that are unrelated to ethnic accommodation. First, to account for the higher risks of nationalist outbidding after nationalist parties enter the political scene (Hislope 1997; Horowitz 1985; Vogt et al. 2021), I construct a measure for the existence of at least one dominant nationalist party (DNP), which I identify using information from the V-Party dataset (Lindberg et al. 2022).^3^ Second, I control for the government access of DNPs, which I capture with a dichotomous indicator that measures whether any DNP is included in the government, again based on V-Party (Lindberg et al. 2022). Such constellations might not only constrain ethnic accommodation, but also create a more permissive environment for dominant group mobilization (cf. Gottlieb and Hislope 2017; Manekin and Mitts 2022). Third, to account for the higher utility of political mobilization in periods preceding elections, I control for the (logged) number of months until the next national election (Hyde and Marinov 2012).

A second set of controls addresses a key inferential challenge: the empirical pattern whereby ethnic tensions often precede the accommodation of subordinate groups. Besides increasing the chance of concessions, such tensions might *themselves* generate counter-mobilization (Basta 2021; Hennayake 1992). To account for this potential source of bias, I incorporate three controls. First, I control for the incidence of protests involving subordinate groups in the last 3 months, based on the ethnically-attributed information on anti-government protest presented above (Clark and Regan 2016). Second, I control for the recent incidence of civil war violence involving subordinate groups in the last 3 months, based on UCDP (Sundberg et al. 2012) and ACD2EPR (Vogt et al. 2015). Finally, I control for the (logged) number of battle-related deaths in the last 10 years (Davies et al. 2022; Lacina and Gleditsch 2005), which might remain a long-term source of polarized inter-ethnic relations.

I also include a series of standard structural and institutional controls. These capture factors that might simultaneously influence the prospect of accommodation and the incidence of dominant group mobilization. First, I account for unfinished or partial democratization processes during which opportunistic nationalist mobilization may be particularly widespread (Snyder 2000). I do so by controlling for each country’s degree of liberal democracy, as captured by the corresponding V-Dem index (Coppedge et al. 2020). Second, I control for the (logged) absolute size of the dominant group. This shapes the baseline probability that its mobilization events are reported in the media sources underlying my data. Moreover, it might also influence the dominant group’s receptivity towards accommodation. Third, I control for GDP per capita and GDP growth in a given year to account for the lower opportunity costs of mass mobilization during economic downturns.^4^

Finally, I account for the potential cross-national diffusion of dominant group mobilization by controlling for the (logged) number of dominant group mobilization events in the preceding month across all countries from the same world region as defined by the United Nations’ geoscheme. Moreover, to account for time dependence, I incorporate a cubic term counting the months since the last observed mobilization event involving a given dominant group. Table 2 provides descriptive statistics for all these variables.

**Table 2.** Descriptive Statistics.

Statistic	*N*	Mean	St. Dev.	Min	Max
No. DG mobilization events	38,130	0.323	1.159	0	56
No. DG anti-government protests	38,130	0.278	0.890	0	31
No. DG anti-minority violent incidents	38,130	0.046	0.718	0	53
Concession number	38,130	0.081	0.404	0	6
Concession number (group-based)	38,130	0.035	0.220	0	3
Concession number (group-blind)	38,130	0.046	0.240	0	3
Months to next election (log)	38,130	2.792	0.990	0.000	5.257
Recent subordinate group protest	38,130	0.060	0.238	0	1
Recent civil violence	38,130	0.143	0.350	0	1
Battle deaths (last 10 y, log)	38,130	0.448	0.922	0.000	5.141
DN party	38,130	0.453	0.498	0	1
DN party in government	38,130	0.295	0.456	0	1
Democracy level	38,130	0.413	0.248	0.017	0.861
Abs. size (log)	38,130	2.108	1.270	0.025	5.867
GDP p.c. (log)	38,130	8.818	1.136	5.870	11.357
GDP growth	38,130	0.017	0.072	−1.647	0.584
Regional DG mobilization events (log)	38,130	1.036	0.766	0.000	4.043
Months without DG mobilization	38,130	15.409	27.970	1	339
Year	38,130	2,004.522	8.218	1,990	2,018

## Country Month-Level Analyses

Throughout my main analyses, which I conduct at the level of the country month, I use negative binomial models to estimate the effect of concessions on the monthly number of dominant group mobilization events. In all models, I incorporate fixed effects at the country-level, which restrict the analysis to over-time variation within each country. This is essential for two reasons. First, it enables me to net out country-specific, time-invariant confounders that might critically shape the probability of both accommodation and dominant group mobilization, for example national founding narratives, colonial legacies, or the type of ethnic cleavages. Second, this approach is consistent with my theoretical argument, which has highlighted over-time variation within each country, most notably the temporal proximity to concessions. To account for secular time trends and common global shocks, I additionally incorporate year-fixed effects across my models.

### Main Results

**Figure 3.**
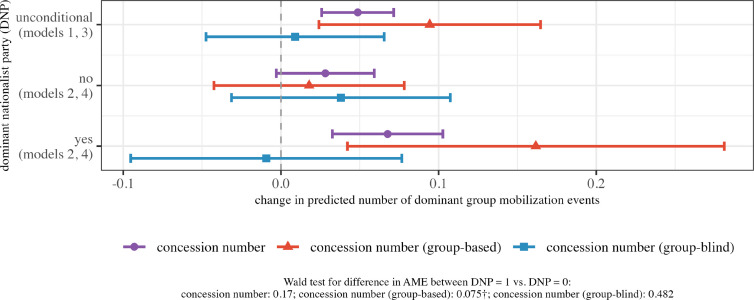
Main model results: Concessions for subordinate groups and change in monthly number of anti-government protests and targeted violent incidents involving dominant groups. Partial effects and 90 percent confidence interval of *concessions*, *group-based concessions*, and *group-blind concessions* on the number of dominant group mobilization events for observed values in my sample [based on models 1–4 in Table 3].

Table 3 shows the results of four models. In model 1, I examine how the number of dominant group mobilization events is affected by concessions, irrespective of their type (hypothesis 1). In model 2, I interact this term with my term for the existence of at least one *dominant nationalist party (DNP)*, to account for the hypothesized conditional relationship (hypothesis 2). Because the estimates are determined by an interactive term in this model, I rely on a Wald test for joint significance at the bottom of the Table. In models 3 and 4, I repeat this procedure, but introduce the distinction between group-based and formally group-blind concessions (hypothesis 3). Figure 3 visualizes the average marginal effects of these terms on the number of mobilization events for the observed values in the sample.

**Table 3.** Ethnic Accommodation and the Number of Mobilization Events Involving the Dominant Group.

	Model 1	Model 2	Model 3	Model 4
Concession number	0.147^***^	0.092		
	(0.039)	(0.060)		
Concession number × DN party		0.095		
		(0.081)		
Concession number (group-based)			0.267^*^	0.059
			(0.107)	(0.119)
Concession number (group-based) × DN party				0.340^†^
				(0.188)
Concession number (group-blind)			0.028	0.123
			(0.107)	(0.129)
Concession number (group-blind) × DN party				−0.150
				(0.205)
DN party	0.082	0.070	0.080	0.069
	(0.166)	(0.165)	(0.165)	(0.163)
DN party in government	0.041	0.046	0.043	0.049
	(0.093)	(0.093)	(0.094)	(0.094)
Months to next election (log)	−0.059^**^	−0.060^**^	−0.061^**^	−0.062^**^
	(0.023)	(0.023)	(0.023)	(0.023)
Recent minority protest	0.385^***^	0.386^***^	0.38^***^	0.387^***^
	(0.082)	(0.082)	(0.082)	(0.082)
Recent civil violence	0.145	0.144	0.144	0.143
	(0.122)	(0.122)	(0.121)	(0.120)
Battle deaths (last 10 y, log)	0.065	0.066	0.066	0.069
	(0.072)	(0.072)	(0.072)	(0.071)
Democracy level	−0.400	−0.404	−0.383	−0.401
	(0.324)	(0.327)	(0.330)	(0.326)
Abs. size (log)	0.210	0.212	0.210	0.219
	(0.183)	(0.182)	(0.182)	(0.179)
GDP p.c. (log)	−0.222	−0.225	−0.214	−0.215
	(0.298)	(0.299)	(0.297)	(0.297)
GDP growth	−0.941^†^	−0.931^†^	−0.961^†^	−0.960^†^
	(0.502)	(0.503)	(0.506)	(0.508)
Regional maj. mobilization events (log)	0.067^*^	0.067^*^	0.067^*^	0.067^*^
	(0.029)	(0.029)	(0.029)	(0.029)
Constant	0.656	0.687	0.566	0.574
	(3.248)	(3.253)	(3.231)	(3.226)
Wald-test Chisq				
Joint sig. int. concession		0.001**		
Joint sig. int. concession (group-based)				0.007**
Joint sig. int. concession (group-blind)				0.86
N	38,130	38,130	38,130	38,130
Log Likelihood	−23,038.070	−23,036.680	−23,035.530	−23,031.730
*θ*	0.513^***^ (0.014)	0.513^***^ (0.014)	0.513^***^ (0.014)	0.514^***^ (0.014)
AIC	46,412.130	46,411.360	46,409.060	46,405.460

†*p* < .1; *p < .05; **p < .01; ***p < .001; country-clustered SE’s in parentheses; country- and year-fixed effects and cubic terms for group-wise months without mobilization included but not reported.

The results from these models offer consistent support for my hypotheses. Model 1 shows that dominant group mobilization is more frequent in 3-month time windows around times during which concessions are introduced (hypothesis 1). As shown in Figure 3 (line 1), this relationship is precisely identified and substantial: For each additional concession introduced during this time window, the predicted number of dominant group mobilization events increases by 0.05. This is a substantial increase, relative to the mean number of such events across all country months in my sample (0.32).

Model 2 indicates that the mobilization-inducing effect of concessions is conditional on the existence of a dominant nationalist party (DNP) (hypothesis 2). In months without any DNP, the estimated partial effect of concessions drops to 0.03. Conversely, the magnitude of this effect increases to 0.07 in contexts where a DNP competes for national office (see Figure 3, lines 4 and 7). As indicated by the corresponding Wald test reported at the bottom of Table 3, this interaction is jointly significant at the 0.05-level. However, the difference in partial effects fails to reach conventional levels of statistical significance (*p* = .17, see Figure 3).

Finally, models 3 and 4 point to critical differences between group-based concessions and those that formally remain group-blind (hypothesis 3). Each group-based concession increases the number of predicted mobilization events in the analyzed 3-month time window by 0.09 (Figure 3, line 2). In contrast, the estimated impact of group-blind concessions is indistinguishable from 0 (line 3). In addition, I find greater support for hypothesis 2 as regards specifically group-based concessions; in particular, my results indicate that the partial effect of group-based concessions increases substantially only in contexts where a DNP competes for office (Figure 3, lines 5 and 8). The interaction terms again reach joint significance at the 0.05-level and the difference in partial effects reaches conventional levels of statistical significance (*p* = .075). This is consistent with my argument that group-based concessions best lend themselves to the articulation of grievances and fears by dominant nationalist parties and thereby enables them to generate a disproportionate amount of collective action.

In appendices 2 and 3, I show that these results remain robust to different operationalizations of key variables, variegated definitions of the politically-dominant group, a narrower sample that excludes democratizing periods from the analysis, the incorporation of additional controls for time-varying shocks, and alternative specifications.

### Addressing Reverse Causation and Endogeneity

While my findings are remarkably robust, I cannot infer from them that accommodation causally affects dominant group mobilization as indicated. A first concern is reverse causation, whereby dominant group mobilization may influence the prospect of future concessions. While such patterns likely affect my findings, actual or anticipated mobilization by dominant groups should make substantial concessions to subordinate groups *less*, and not more, likely. Most notably, the risk of a backlash should dissuade policymakers from offering group-based concessions in the first place (Basta 2021; Hennayake 1992). If such tendencies were widespread, my results would hence underestimate the effect of group-based concessions on dominant group mobilization. In line with this expectation, a reverse analysis indicates that, if anything, dominant group mobilization in the past 5 years is associated with a lower probability of group-based concessions and, conversely, with a higher probability of group-blind concessions (appendix 1.1).

A second concern is that the relationship between concessions and dominant group mobilization may be endogenous to third factors. As discussed above, severe ethnic conflict often precedes the provision of ethnic accommodation (Cederman et al. 2013, 2015). Problematically for my results, this is especially likely for group-based concessions, whose rigid reassurances and symbolic recognition are especially sought-after by minorities following violent conflict (Bogaards 2019; Lijphart 1995; McCulloch 2014). In line with this expectation, the above-referenced reverse analyses indicate that the provision of group-based concessions is more likely following widespread protests by subordinate groups (appendix 1.1). Problematically, such mobilization can also generate a backlash for reasons unrelated to concessions, for example against far-reaching minority demands (Hennayake 1992).

Ideally, these concerns would be addressed by identifying (quasi-)random variation in group-based concessions, which is not influenced by preceding ethnic tensions. However, such quasi-random variation is generally not available for large-scale policy choices, such as national-level power-sharing institutions and the constitutional recognition of minorities’ cultural customs. Instead, policymakers adopt accommodation for a variety of reasons. These include domestic strategic considerations (Roessler and Ohls 2018; Walter 2009), normative and ideological reasons—especially relevant for moderate and progressive political actors (Bustikova 2014)—, regional institutional templates (Cederman et al. 2018), or external pressure, for instance from conflict mediators (McCulloch and McEvoy 2018). In many cases, it is difficult to disentangle these factors, making it challenging to isolate the role played by preceding ethnic tensions in policymakers’ decision to introduce or expand accommodation.

To probe these concerns, I conduct three analytical procedures. First, I conduct a causal sensitivity analysis, following the approach by Cinelli and Hazlett (2020) (appendix 1.2). This approach shows that my findings are robust even to severe omitted variable bias. In particular, my findings are robust to the presence of a confounder that is at least equally (for concessions) or twice as strong (for group-based concessions) as past protests of subordinate groups, a proxy measure for ethnic tensions. Second, I conduct a difference-in-difference analysis which replicates my findings and shows no evidence for a pretrend in dominant group mobilization before concessions (appendix 1.3). Third, I split up my sample, distinguishing between contexts that have experienced ethnically based civil wars in the last 10 years and those that have not (appendix 1.4). Reassuringly, my main findings are replicated in both sub-samples.

### Alternative Explanations

Even if the attained relationships between accommodation and dominant group mobilization are robust to omitted variable bias, they could be accounted for by alternative mechanisms. In appendix 2, I address this possibility. First, I show that my findings are driven by ethnic accommodation specifically and not by wider constitutional or political shifts that might coincide with such concessions, including the adoption of new constitutions, national elections, the signing of peace agreements and ceasefires, and the incidence of coups.

Second, I address the risk that my results might be driven by mobilization events that involve members of dominant groups, yet are not directly related to the accommodation of subordinate groups. This risk looms especially large for protests, which make up a large share of the variation in my dependent variable, and which I coded based on protester identities, as opposed to the substantive issues addressed by protests. To address this risk, I show that my results can be replicated when only incorporating protests into my dependent variable that are related to questions of institutional design and minority rights.

Third, I show that my findings are likely driven by dominant group-initiated mobilization, as opposed to violence that may also be initiated by subordinate groups.

Fourth, and finally, I examine whether my findings might be due to narrow elite opposition to ethnic power-sharing, as opposed to grievances and fears among ordinary members of the dominant community. In line with my argument and contra this alternative explanation, I find that even overwhelmingly symbolic concessions that do not restrict elites’ influence directly, such as the recognition of subordinate groups’ cultural practices, can generate dominant group mobilization.

## Individual-Level Analyses

To corroborate my hypothesized mechanisms, I probe the intermediate implications of these arguments in a series of individual-level analyses (appendix 4). If my mechanisms apply, concessions should be associated with a higher willingness to mobilize among ordinary members of the dominant group, especially among conservative and authoritarian individuals. If it is possible to show such patterns, this will increase confidence in my postulated causal chain. In contrast, it will further diminish the plausibility of the above-discussed alternative explanations, which are difficult to completely dispel using information on observed mobilization alone.

To trace the observable implications of these arguments at the individual level, I rely on attitudinal data taken from the Integrated Values Surveys (IVS) (Inglehart et al. 2014). I identify respondents belonging to the politically-dominant group in the month each survey wave was administered, using information on respondent ethnicity by Juon (2023). Altogether, my sample encompasses 176,635 respondents, clustered in 73 countries. My dependent variable is each respondent’s stated *willingness to protest*. This takes the value of 1 if respondents indicated that they have recently participated in a peaceful demonstration or are willing to do so in the future. Conversely, it takes the value of 0 for respondents who state they are unwilling to do so.

Owing to the rarity of concessions, and the infrequent administration of IVS surveys, my independent variables need to be adjusted for this auxiliary analysis. Hence, instead of examining the effect of the number of concessions in narrow time windows, I examine the enduring consequences of *past* concessions that shape respondents’ assessments of the current constitutional system. I rely on the Comparative Constitutions Project (Elkins et al. 2014) to identify the constitutional system that is in place in each country month. Using my data on the timing of concessions, I then create dichotomous variables that take the value 1 if there have been (group-based or formally group-blind) concessions since the constitution has been introduced, and 0 otherwise. While not ideal, this operationalization reflects my argument that accommodation creates a persistently larger reservoir of dominant group members who are willing to engage in non-institutional mobilization reasonably well.

**Figure 4.**
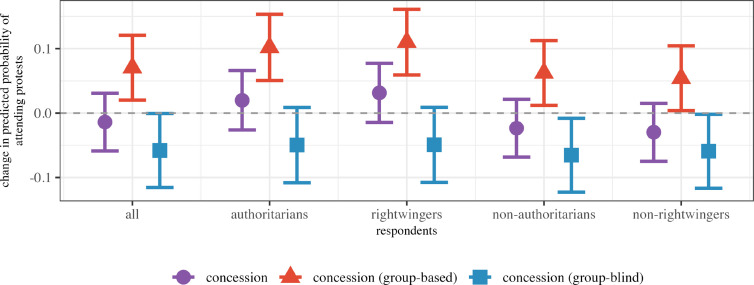
Individual-level analysis results: Past concessions during constitutional system and change in dominant group respondents’ willingness to participate in protests. Partial effects and 90 percent confidence interval of past concessions, group-based concessions, and group-blind concessions during constitutional system on the predicted probability of respondents stating they have attended or would attend protests [based on models 1–6 in ATable 4 (appendix 4.3)].

Using this information, I estimate how past concessions affect dominant group members’ willingness to protest in a set of hierarchical multilevel models (see appendix 4). This allows me to probe whether such concessions increase willingness to protest especially among individuals who are politically right-wing^5^ or hold authoritarian value orientations.^6^ Figure 4 summarizes the main results of these models.

I find that members of dominant groups are more willing to protest if there has been at least one group-based concession during the current constitutional system. Moreover, these tendencies are particularly pronounced for respondents who self-identify as right-wing or hold authoritarian value orientations. This particular finding is in accordance with my hypothesized mechanisms, which posited that these individuals should be especially likely to form grievances and fears in response to concessions. In contrast, it is less compatible with or not predicted by the alternative explanations discussed above. For instance, if elite-level opposition to restrictive power-sharing institutions explained my results, we would not necessarily expect to observe higher willingness to protest among ordinary citizens. Similarly, if my results were driven by broader political shifts, and not concessions specifically, we would not necessarily expect to see an especially pronounced willingness to protest among conservative and authoritarian members of dominant groups.

## Conclusion

In this article, I set out to shed light on a theoretical and empirical puzzle: the variable incidence of a backlash by dominant groups against the accommodation of subordinate groups. I have presented evidence from one of the first comprehensive data sets with temporally fine-grained information on concessions that provide subordinate groups with representation in government, regional autonomy, and recognition of their cultural practices. I have combined this with ethnically-attributed information on the monthly number of anti-government protests and targeted violence involving members of the politically-dominant community. Expanding previous studies’ focus on select, prominent cases, my analysis covers all multiethnic electorally competitive regimes between 1990 and 2018. This enables me to conduct a systematic assessment of the relationship between ethnic accommodation and political mobilization by dominant groups for the full period since the end of the Cold War.

My findings show that dominant group mobilization is especially frequent around times when accommodation is first adopted or expanded. In line with my argument, this association is particularly pronounced if a dominant nationalist party competes for political office. Moreover, I find that it is driven by the introduction of formal, constitutional provisions that explicitly recognize subordinate groups’ collective identities, for instance the provision of ethnic government quotas or veto rights, the designation of specific regions as subordinate groups’ homelands, and the recognition of specific groups’ cultural practices.

These findings underline the need to address the risk of a backlash when states first introduce or expand ethnic accommodation. Of course, the incidence of such a backlash does not mean that multiethnic states should forsake accommodation as a strategy of conflict management, given its important benefits for both peace and post-conflict stability (Cederman et al. 2013; McGarry et al. 2008). However, if it becomes large-scale or turns violent, this backlash can demonstrably threaten foundational public goods, such as democratic stability (Pevnick 2024), bring to power radical right actors who seek to unravel minority rights more broadly (Bustikova 2014, 2019), or even spark large-scale violence against minorities (Gottlieb and Hislope 2017; Straus 2006). For international conflict mediators and moderate domestic actors who rightly promote ethnic accommodation, being aware of this risk can be a first step to mitigating its destabilizing potential. Most importantly, my results point to concrete proposals that are less susceptible to a backlash and hence minimize this risk.

At the most fundamental level, my findings offer good news for advocates of ethnic accommodation. I do not find that accommodation, such as the informal inclusion of minority members in government, generates opposition by dominant groups across the board. This attenuates concerns that dominant groups may be fundamentally opposed to ethnic accommodation, as prominently voiced by both its proponents and skeptics (Horowitz 2014; Lijphart 1996). However, my findings indicate that formal, group-based concessions, such as ethnic government quotas, are contentious and can be subject to a pronounced backlash, especially if dominant nationalist parties are engaged in electoral competition. In such contexts, policy-makers might hence explore the possibility of accommodating subordinate groups through formally group-blind concessions that are less prone to a backlash, for instance informal power-sharing pacts, electoral power-sharing institutions, and the provision of expanded policy and fiscal competencies to subnational regions.

These findings suggest important areas for further research. First, scholars might explore other possibilities to mitigate the risk of a backlash against accommodation, for instance by reassuring members of the dominant group that ethnic accommodation does not threaten their political and economic status. Second, my analysis has exclusively focused on the *short-term* consequences of ethnic accommodation, within 3 month time windows around concessions. This does not rule out that ethnic accommodation can garner increased acceptance over time or could even *decrease* dominant group mobilization in the long run. Future research should examine this possibility. Third, I have focused on the accommodation of “native” ethnic groups. However, dominant groups’ grievances and fears might similarly be directed against the empowerment of other groups that were previously politically subordinate, such as women or international migrants (Sidanius et al. 2017). Fourth, future research might examine in more detail differences between various *types* of dominant groups, for instance those that constitute a demographic plurality and those that are a minority. Finally, interactions between rival nationalist movements that represent subordinate and dominant groups, for and against ethnic accommodation (Basta 2021; Hennayake 1992), should be more explicitly investigated than has been possible in this study.

All in all, this study shows that scholars and decision makers need to take the possibility of a backlash by dominant groups against the accommodation of subordinate groups seriously and explore possibilities to mitigate them, in order to secure the political and democratic benefits of ethnic accommodation.

## Supplemental Material

Supplemental Material - Ethnic Accommodation and the Backlash From Dominant Groups Supplemental Material for Ethnic Accommodation and the Backlash From Dominant Groups by Andreas Juon in Journal of Conflict Resolution

Supplemental Material - Ethnic Accommodation and the Backlash From Dominant Groups Supplemental Material for Ethnic Accommodation and the Backlash From Dominant Groups by Andreas Juon in Journal of Conflict Resolution

Supplemental Material - Ethnic Accommodation and the Backlash From Dominant Groups Supplemental Material for Ethnic Accommodation and the Backlash From Dominant Groups by Andreas Juon in Journal of Conflict Resolution

## Data Availability

All data and R scripts used to conduct this article's analyses is made publicly available for further use to other researchers under https://doi.org/10.7910/DVN/GKULIW(Juon 2025).

## Notes

1. I identify group-based (group-blind) horizontal concessions in months where a group’s corporate (liberal) power-sharing index increases by a value of at least 0.1 (Juon 2023). I also identify group-blind horizontal concessions in months where a group becomes newly included in the executive (Vogt et al. 2015) that were not preceded by corresponding increases of its corporate/liberal power-sharing indices in the preceding 12 months.

2. For this purpose, I sum up the number of concession types given to at least one subordinate group in each given month: corporate power-sharing, liberal power-sharing, informal government representation, increased cultural rights, and increased autonomy.

3. In data supplement S3, I explain these coding rules and provide a complete list of all identified dominant nationalist parties in my sample.

4. Predominantly based on Penn World Tables 7.1 (PWT, Heston et al. 2012).

5. My variable *rightwinger* takes the value of 1 for respondents that classify themselves as 7 or higher on a ten-point left-right scale, and 0 otherwise.

6. My variable *authoritarian* takes the value 1 for respondents who state that maintaining order is one of the most important goals of society and simultaneously opine that having a political system with a strong leader is “very good” or “fairly good”, and 0 otherwise.

## References

[bibr1-00220027251343836] Basta K. 2016. “Imagined Institutions: The Symbolic Power of Formal Rules in Bosnia and Herzegovina.” Slavic Review 75 (4): 944-969.

[bibr2-00220027251343836] Basta K. 2021. The Symbolic State: Minority Recognition, Majority Backlash, and Secession in Multinational Countries. Number 7 in Democracy, Diversity, and Citizen Engagement Series. McGill-Queen’s University Press.

[bibr3-00220027251343836] Benford R. D. Snow D. A. . 2000. “Framing Processes and Social Movements: An Overview and Assessment.” Annual Review of Sociology 26 (1): 611-639.

[bibr4-00220027251343836] Bogaards M. 2019. “Iraq’s Constitution of 2005: The Case Against Consociationalism ‘Light’.” Ethnopolitics 20: 186.

[bibr5-00220027251343836] Bonikowski B. 2016. “Nationalism in Settled Times.” Annual Review of Sociology 42 (1): 427-449.

[bibr6-00220027251343836] Bormann N.-C. Cederman L.-E. Vogt M. . 2017. “Language, Religion, and Ethnic Civil War.” Journal of Conflict Resolution 61 (4): 744-771.

[bibr7-00220027251343836] Brass P. 1991. Ethnicity and Nationalism: Theory and Comparison. Sage.

[bibr8-00220027251343836] Brunnbauer U. 2002. “The Implementation of the Ohrid Agreement: Ethnic Macedonian Resentments.” JEMIE - Journal on Ethnopolitics and Minority Issues in Europe 1: 1-23.

[bibr9-00220027251343836] Bulutgil H. Z. 2016. The Roots of Ethnic Cleansing in Europe. Cambridge University Press.

[bibr10-00220027251343836] Bustikova L. 2014. “Revenge of the Radical Right.” Comparative Political Studies 47 (12): 1738-1765.

[bibr11-00220027251343836] Bustikova L. 2019. Extreme Reactions: Radical Right Mobilization in Eastern Europe. 1st ed. Cambridge University Press.

[bibr12-00220027251343836] Cederman L.-E. Gleditsch K. S. Buhaug H. . 2013. Inequality, Grievances, and Civil War. Cambridge University Press.

[bibr13-00220027251343836] Cederman L.-E. Hug S. Schädel A. Wucherpfennig J. . 2015. “Territorial Autonomy in the Shadow of Conflict: Too Little, Too Late?” American Political Science Review 109 (02): 354-370.

[bibr14-00220027251343836] Cederman L.-E. Gleditsch K. S. Wucherpfennig J. . 2018. “The Diffusion of Inclusion: An Open-Polity Model of Ethnic Power Sharing.” Comparative Political Studies 51 (10): 1279-1313.

[bibr15-00220027251343836] Cinelli C. Hazlett C. . 2020. “Making Sense of Sensitivity: Extending Omitted Variable Bias.” Journal of the Royal Statistical Society Series B: Statistical Methodology 82 (1): 39-67.

[bibr16-00220027251343836] Clark D. Regan P. (2016). “Mass Mobilization Protest Data.

[bibr17-00220027251343836] Clark N. L. Worger W. H. . 2011. South Africa: The Rise and Fall of Apartheid. 2nd ed. Longman.

[bibr18-00220027251343836] Coakley J. 2011. “National Majorities in New States: Managing the Challenge of Diversity.” In Contemporary Majority Nationalism, Studies in Nationalism and Ethnic Conflict, edited by Gagnon A. Lecours A. Nootens G. , 101-126. McGill-Queen’s University Press.

[bibr19-00220027251343836] Coppedge M. Gerring J. Knutsen C. H. , et al. 2020. V-Dem Methodology V10. Varieties of Democracy (V-Dem) Project.

[bibr20-00220027251343836] Davies S. Pettersson T. Öberg M. . 2022. “Organized Violence 1989–2021 and Drone Warfare.” Journal of Peace Research 59 (4): 593-610.

[bibr21-00220027251343836] Desrosiers M.-E. 2012. “Reframing Frame Analysis: Key Contributions to Conflict Studies.” Ethnopolitics 11 (1): 1-23.

[bibr22-00220027251343836] Elkins Z. Ginsburg T. Melton J. . 2014. “The Comparative Constitutions Project: A Cross-National Historical Dataset of Written Constitutions.” https://www.comparativeconstitutionsproject.org/

[bibr23-00220027251343836] Gamson W. A. 2013. “Injustice Frames.” In The Wiley-Blackwell Encyclopedia of Social and Political Movements, edited by Snow D. A. della Porta D. Klandermans B. McAdam D. . Wiley-Blackwell.

[bibr24-00220027251343836] Germann M. Sambanis N. . 2021. “Political Exclusion, Lost Autonomy, and Escalating Conflict over Self-Determination.” International Organization 75 (1): 178-203.

[bibr25-00220027251343836] Ghai Y. P. 2002. “Constitutional Asymmetries: Communal Representation, Federalism, and Cultural Autonomy.” In The Architecture of Democracy: Constitutional Design, Conflict Management, and Democracy, Oxford Studies in Democratization, edited by Reynolds A. , 104-140. Oxford University Press.

[bibr26-00220027251343836] Girvin B. 2020. “From Civic Pluralism to Ethnoreligious Majoritarianism: Majority Nationalism in India.” Nationalism and Ethnic Politics 26 (1): 27-45.

[bibr27-00220027251343836] Gottlieb E. Hislope R. . 2017. “Ethnic Divisions and Riots in Macedonia.” In Identity Conflicts: Can Violence Be Regulated? edited by Jenkins J. C. Gottlieb E. E. . 1st ed., 149-165. Routledge.

[bibr28-00220027251343836] Gurr T. R. 2002. “Attaining Peace in Divided Societies: Five Principles of Emerging Doctrine.” International Journal on World Peace 19: 27-51.

[bibr29-00220027251343836] Hennayake S. K. 1992. “Interactive Ethnonationalism. An Alternative Explanation of Minority Ethnonationalism.” Political Geography 11 (6): 526-549.

[bibr30-00220027251343836] Heston A. Summers R. Aten B. . 2012. “Penn World Table Version 7.1.” https://pwt.sas.upenn.edu/php_site/pwt_index.php

[bibr31-00220027251343836] Hislope R. 1997. “Intra-Ethnic Conflict in Croatia and Serbia: Flanking and the Consequences for Democracy.” East European Quarterly 30: 471-494.

[bibr32-00220027251343836] Horowitz D. L. 1985. Ethnic Groups in Conflict. University of California Press.

[bibr33-00220027251343836] Horowitz D. L. 2014. “Ethnic Power Sharing: Three Big Problems.” Journal of Democracy 25 (2): 5-20.

[bibr34-00220027251343836] Hyde S. D. Marinov N. . 2012. “Which Elections Can Be Lost?” Political Analysis 20 (2): 191-201.

[bibr35-00220027251343836] Inglehart R. Haerpfer C. Moreno A. , et al. 2014. World Values Survey: All Rounds - Country-Pooled Datafile Version. JD Systems Institute.

[bibr36-00220027251343836] Jenne E. K. 2007. Ethnic Bargaining: The Paradox of Minority Empowerment. Cornell University Press.

[bibr37-00220027251343836] Juon A. 2023. “Inclusion, Recognition, and Inter-Group Comparisons: The Effects of Power-Sharing Institutions on Grievances.” Journal of Conflict Resolution 67 (9): 1783-1810.

[bibr68-00220027251343836] Juon A. 2025. “Replication Data for: ‘Ethnic accommodation and the backlash from dominant groups’ published in the Journal of Conflict Resolution (JCR)”. doi:10.7910/DVN/GKULIW PMC12782309 41522262

[bibr38-00220027251343836] Kahneman D. Tversky A. . 1979. “Prospect Theory: An Analysis of Decision Under Risk.” Econometrica 47 (2): 263.

[bibr39-00220027251343836] Lacina B. Gleditsch N. P. . 2005. “Monitoring Trends in Global Combat: A New Dataset of Battle Deaths.” European Journal of Population / Revue européenne de Démographie 21 (2-3): 145-166.

[bibr40-00220027251343836] Lijphart A. 1995. “Self-Determination versus Pre-Determination of Ethnic Minorities in Power-Sharing Systems.” In The Rights of Minority Cultures, edited by Kymlicka W. , 275-287. Oxford University Press.

[bibr41-00220027251343836] Lijphart A. 1996. “The Puzzle of Indian Democracy: A Consociational Interpretation.” American Political Science Review 90 (02): 258-268.

[bibr42-00220027251343836] Lijphart A. 2004. “Constitutional Design for Divided Societies.” Journal of Democracy 15 (2): 96-109.

[bibr43-00220027251343836] Lindberg S. I. Düpont N. Higashijima M. , et al. 2022. Varieties of Party Identity and Organization (V-Party) Dataset V2. Varieties of Democracy (V-Dem) Project.

[bibr44-00220027251343836] Maerz S. F. Edgell A. Wilson M. C. Hellmeier S. Lindberg S. I. (2021). “A Framework for Understanding Regime Transformation: Introducing the ERT Dataset.

[bibr45-00220027251343836] Manekin D. Mitts T. . 2022. “Effective for Whom? Ethnic Identity and Nonviolent Resistance.” American Political Science Review 116 (1): 161-180.

[bibr46-00220027251343836] McAdam D. Tarrow S. . 2000. “Nonviolence as Contentious Interaction.” PS: Political Science & Politics 33 (2): 149-154.

[bibr47-00220027251343836] McCulloch A. 2014. “Consociational Settlements in Deeply Divided Societies: The Liberal-Corporate Distinction.” Democratization 21 (3): 501-518.

[bibr48-00220027251343836] McCulloch A. McEvoy J. . 2018. “The International Mediation of Power-Sharing Settlements.” Cooperation and Conflict 53 (4): 467-485.

[bibr49-00220027251343836] McGarry J. O’Leary B. Simeon R. . 2008. “Integration or Accommodation? The Enduring Debate in Conflict Regulation.” In Constitutional Design for Divided Societies. Integration or Accommodation? edited by Choudhry S. , 41-90. Oxford University Press.

[bibr50-00220027251343836] Mintz A. Valentino N. A. Wayne C. . 2022. Beyond Rationality: Behavioral Political Science in the 21st Century. Cambridge University Press.

[bibr51-00220027251343836] Müller-Crepon C. 2022. “Local Ethno-Political Polarization and Election Violence in Majoritarian vs. Proportional Systems.” Journal of Peace Research 59 (2): 242-258. 35370306 10.1177/0022343320973724 PMC8969075

[bibr52-00220027251343836] Mylonas H. 2012. The Politics of Nation-Building. Making Co-Nationals, Refugees, and Minorities. Cambridge University Press.

[bibr53-00220027251343836] Norris P. Inglehart R. . 2019. Cultural Backlash: Trump, Brexit, and Authoritarian Populism. 1st ed. Cambridge University Press.

[bibr54-00220027251343836] Petersen R. D. 2002. Understanding Ethnic Violence: Fear, Hatred, and Resentment in Twentieth-Century Eastern Europe. Cambridge University Press.

[bibr55-00220027251343836] Pettersson T. Högbladh S. Öberg M. . 2019. “Organized Violence, 1989–2018 and Peace Agreements.” Journal of Peace Research 56 (4): 589-603.

[bibr56-00220027251343836] Pevnick R. 2024. “Immigration, Backlash, and Democracy.” American Political Science Review 118 (1): 332-344.

[bibr57-00220027251343836] Roessler P. Ohls D. . 2018. “Self-Enforcing Power Sharing in Weak States.” International Organization 72 (2): 423-454.

[bibr58-00220027251343836] Sidanius J. Cotterill S. Kteily N. Carvacho H. . 2017. “Social Dominance Theory: Explorations in the Psychology of Oppression.” In The Cambridge Handbook of the Psychology of Prejudice, edited by Sibley C. G. Barlow F. , 149-187. Cambridge University Press.

[bibr59-00220027251343836] Snyder J. 2000. “From Voting to Violence.” In Democratization and Nationalist Conflict. W.W. Norton & Company.

[bibr60-00220027251343836] Straus S. 2006. The Order of Genocide: Race, Power, and War in Rwanda. Cornell University Press.

[bibr61-00220027251343836] Sundberg R. Eck K. Kreutz J. . 2012. “Introducing the UCDP Non-State Conflict Dataset.” Journal of Peace Research 49 (2): 351-362.

[bibr62-00220027251343836] Tamir Y. Y. 2019. “Not So Civic: Is There a Difference Between Ethnic and Civic Nationalism?” Annual Review of Political Science 22 (1): 419-434.

[bibr63-00220027251343836] Vogt M. Bormann N.-C. Rüegger S. Cederman L.-E. Hunziker P. Girardin L. . 2015. “Integrating Data on Ethnicity, Geography, and Conflict: The Ethnic Power Relations Data Set Family.” Journal of Conflict Resolution 59 (7): 1327-1342.

[bibr64-00220027251343836] Vogt M. Gleditsch K. S. Cederman L.-E. . 2021. “From Claims to Violence: Signaling, Outbidding, and Escalation in Ethnic Conflict.” Journal of Conflict Resolution. 65: 1278. 34393264 10.1177/0022002721996436 PMC8358586

[bibr65-00220027251343836] Walter B. F. 2009. Reputation and Civil War: Why Separatist Conflicts are So Violent. Cambridge University Press.

[bibr66-00220027251343836] Wilkinson S. I. 2000. “India, Consociational Theory, and Ethnic Violence.” Asian Survey 40 (5): 767-791.

[bibr67-00220027251343836] Wimmer A. 2015. “Nation Building. A Long-Term Perspective and Global Analysis.” European Sociological Review 31 (1): 30-47.

