# Supplementary material for: Ethnic Accommodation and the Backlash From Dominant Groups
Source: J Conflict Resolut. 2025 May 22;70(2-3):359–86. doi: 10.1177/00220027251343836 (PMC12782309; doi:10.1177/00220027251343836)
Supplement: Supplemental Material - Ethnic Accommodation and the Backlash From Dominant Groups [file sj-pdf-1-jcr-10.1177_00220027251343836.pdf]

Ethnic accommodation and the backlash from dominant groups: Online appendices

Contents

Appendix 1: Reverse causation and endogeneity ... 2
Appendix 1.1: Reverse analysis ... 2
Appendix 1.2: Causal sensitivity analyses ... 4
Appendix 1.3: Difference-in-differences approach ... 6
Appendix 1.4: Subsample analyses ... 7
Appendix 2: Alternative explanations ... 8
Appendix 2.1: Major constitutional and political shifts ... 9
Appendix 2.2: Mobilization unrelated to ethnic issues ... 9
Appendix 2.3: Violence initiated by subordinate groups ... 11
Appendix 2.4: Elite opposition to restrictive, ethnically based power-sharing ... 11
Appendix 3: General robustness checks ... 13
Appendix 3.1: Alterations to independent variables ... 13
Appendix 3.2: Alterations to dependent variable (dominant group mobilization) ... 16
Appendix 3.3: Sample alterations ... 18
Appendix 3.4: Incorporation of additional control variables ... 19
Appendix 3.5: Alternative specifications ... 21
Appendix 4: Individual-level analyses ... 23
Appendix 4.1: Details on survey measures ... 23
Appendix 4.2: Model set-up ... 23
Appendix 4.3: Full model results ... 25
Additional references ... 26

Appendix 1: Reverse causation and endogeneity

In this section, I probe the susceptibility of my findings to reverse causation and endogeneity to omitted confounders. As stated in the main article, I am most concerned about an empirical pattern whereby severe ethnic tensions often precede the provision of group-based accommodation (Cederman et al. 2013, 2015). At the same time, such ethnic tensions may themselves generate dominant group mobilization for reasons unrelated to concessions, for example in the form of a backlash against controversial demands by subordinate groups or against recent civil war-related violence (Basta 2021; Hennayake 1992). As a result, my results might overestimate the true effect of (group-based) concessions on the frequency of dominant group mobilization. In this appendix, I conduct four analytical procedures that jointly address this concern: a reverse analysis that empirically examines the determinants of concessions in my sample (appendix 1.1), a causal sensitivity analysis that examines the susceptibility of my findings to unobserved confounders such as latent ethnic tensions (appendix 1.2), a difference-in-differences analysis that allows me to probe the potential existence of a pretrend in dominant group mobilization before concessions are granted (appendix 1.3), and a split-sample analysis that distinguishes between (more polarized) post-conflict and (less polarized) stable contexts (appendix 1.4). In what follows, I mostly confine myself to reporting the main findings graphically. The supplementary R- and Stata-scripts generate tables that contain the full model results.

Appendix 1.1: Reverse analysis

In a first step, I conduct a reverse analysis that examines the determinants of concessions that provide for ethnic accommodation. As I have argued in the main article text, policymakers will consider the risk of mobilization by both dominant *and* subordinate groups. On the one hand, actual or anticipated dominant group mobilization should constrain policymakers and dissuade them from offering far-reaching concessions to subordinate groups (cf. Basta 2021; Hennayake 1992). Most notably, instead of offering contentious group-based concessions, governing elites might try to substitute less contentious group-blind concessions for them, to minimize the risk of a backlash. On the other hand, representatives of subordinate groups often vehemently demand group-based concessions, particularly in the aftermath of violent conflict or following histories of systematic discrimination (Bogaards 2019; Lijphart 1995; McCulloch 2014). In turn, where subordinate groups successfully organize large-scale protest or violent rebellion to underline such demands, governing elites will face increased pressure to adopt group-based forms of accommodation (Cederman et al. 2013; 2015).

To probe these expectations empirically, I conduct a series of reverse analyses. I run three logistic regressions, whose dependent variables are binary indicators which capture whether there has been a (group-based/group-blind) concession in each country month in my sample. I capture the countervailing influence mobilization by dominant and subordinate groups with three independent variables. These capture the frequency of past (1) *dominant group mobilization events*, (2) *civil violence incidents* involving subordinate groups, and (3) *subordinate group protests*, all constructed using the data I presented in the main article text. All three variables count the logged number of mobilization events in the respective category in the past five years, while excluding the preceding 12 months, during which mobilization dynamics are more likely to be affected by upcoming constitutional amendments, which are often publicly discussed long before they are promulgated (see also appendix 3.1.1). My control variables are analogous to those used in my main analyses.

Together, these results do not suggest a very strong influence for recent mobilization by dominant and subordinate groups on the adoption of concessions; however, they do reveal patterns that are in accordance with the above expectations (see table A1 for full results and figure A1 for corresponding partial effects). First, in line with the notion of a substitution effect towards less contentious concessions, I find that past dominant group mobilization is negatively associated with the probability of group-based concessions. In contrast, it is positively associated with the probability of group-blind concessions, although both associations clearly do not reach conventional levels of statistical significance. Second, in line with the expected role of subordinate group mobilization, I find that past protests by subordinate groups are positively associated with the probability of group-based concessions, a result that reaches conventional levels of statistical significance.

Together, these results suggest, if anything, that anticipated or actual dominant group mobilization are more likely to constrain policymakers and make the provision of group-based concessions *less*, rather than more likely. As a result, all things being equal, this pattern makes it likely that I *under*estimate the true effect of group-based concessions on dominant group mobilization, as I have argued in the main article text. However, the results also indicate that group-based accommodation is more likely to be adopted or expanded following subordinate group mobilization in the form of protests. This underlines concerns that the attained relationship between group-based concessions and dominant group mobilization might be endogenous to latent ethnic tensions that I can only imperfectly capture with my control variables. In the next three sections, I explore this concern further.

Table A1. Reverse analysis: Dominant group mobilization, subordinate group civil violence and protests, and the provision of concessions to subordinate groups.

	**Model 1 Concession**	**Model 2 Concession (group-based)**	**Model 3 Concession (group-blind)**
Dominant group mobilization events (last 5y, log)	0.081	-0.069	0.125
	(0.089)	(0.131)	(0.150)
Subordinate group civil violence incidents (last 5y, log)	0.009	0.005	0.002
	(0.053)	(0.076)	(0.068)
Subordinate group protests (last 5y, log)	0.019	0.322*	-0.026
	(0.088)	(0.140)	(0.150)
DN party	-0.144	0.152	-0.369
	(0.272)	(0.370)	(0.574)
DN party in government	0.043	0.020	-0.542
	(0.259)	(0.385)	(0.397)
Months to next election (log)	-0.212***	-0.191**	-0.224**
	(0.042)	(0.059)	(0.070)
Battle deaths (last 10y, log)	-0.038	-0.167	0.104
	(0.117)	(0.183)	(0.163)
Democracy level	-0.130	-0.733	2.062
	(1.100)	(1.317)	(1.442)
Abs. size (log)	-0.059	-0.020	0.265
	(0.389)	(0.547)	(0.567)
GDP p.c. (log)	0.350	-1.139	-0.318
	(0.486)	(0.874)	(0.569)
GDP growth	-2.604**	-1.690	-2.145**
	(0.806)	(1.090)	(0.815)
Constant	-10.928*	-6.304	-16.874**
	(5.495)	(9.326)	(6.346)
Country-FE	yes	yes	yes
Year-FE	yes	yes	yes
N	32723	32723	32723
Log Likelihood	-3624.979	-1983.553	-1636.398
AIC	7567.958	4285.107	3590.796

† p<0.1; * p<0.05; ** p<0.01; *** p<0.001; country-clustered SE's in parentheses; cubic terms for country-wise years without (group-based/group-blind) concessions included but not reported.

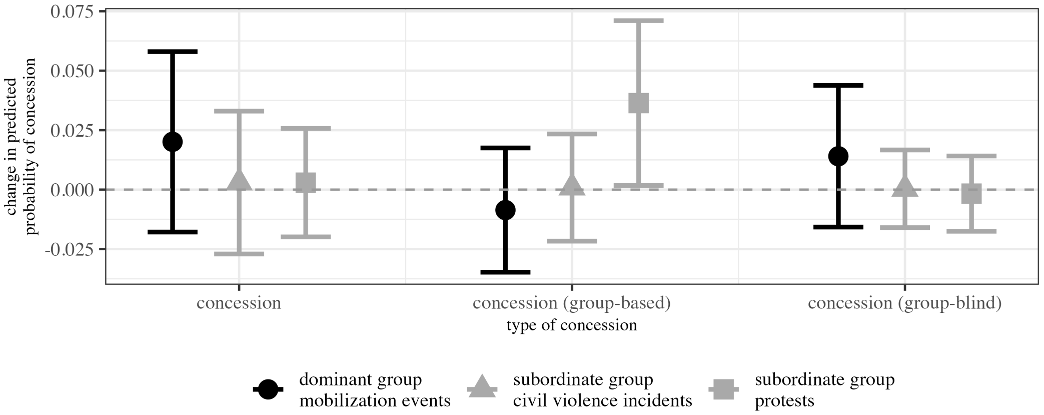

Figure A1. Partial effects and 90% confidence interval of *dominant group mobilization events (last 5y, log)*, *subordinate group civil violence incidents (last 5y, log)*, and *subordinate group protests (last 5y, log)* on the monthly probability of concessions, group-based concessions, and group-blind concessions.

Appendix 1.2: Causal sensitivity analyses

In this and the next two sections, I conduct three analytical procedures that probe the potential of my main finding—the relationship between (group-based) concessions and the number of dominant group mobilization events—to be endogenous to omitted variables or influenced by reverse causation.

In a first step, I conduct a causal sensitivity analysis. This assesses how hypothetical violations of the exogeneity assumption would bias my findings on the effect of *concessions* (model 1) and *group-based concessions* (model 3) on the number of dominant group mobilization events. To assess the sensitivity of my estimates, I follow the procedure developed by Cinelli and Hazlett (2020), using the R-package *sensemakr*. This procedure does not allow the inclusion of interactive terms and requires a linear specification. Hence, I focus exclusively on the main terms of interest—*concessions* and *group-based concessions*—and replace the negative binomial specifications used in my main models with a linear model.

Relying on the linear equivalents to models 1 and 3, I estimate changes in the statistical significance of *(group-based) concessions*, depending on the presence of hypothetical confounders that are associated to varying degrees with both these independent variables and my outcome variable, the *number of dominant group mobilization events*. As a benchmark confounder, I employ *recent subordinate group protest*, which is positively associated both with the probability of (*group-based) concessions* and the *number of dominant group mobilization events* in each month (see also results in appendix 1.1 above).

Figure A2 visualizes the results of this procedure. Its horizontal axis captures the residual variation in *(group-based) concessions* that is explained by the hypothetical confounder; its vertical axis captures the residual variation of the *number of dominant group mobilization events* explained by it. The contour curves show the adjusted t-value that would be obtained in the presence of an unobserved confounder, depending on the hypothetical values of the sensitivity parameters. The red dots highlight scenarios in which there is a confounder that is 1x, 2x, and 3x as strongly correlated with the independent and outcome variables, as compared to the benchmark confounder, *recent minority protest*.

The results indicate that my findings are remarkably robust to omitted variable bias. The coefficient of *concessions* (model 1) remains positive and statistically significant at the 90%-level in the presence of a hypothetical confounder that is at least as strong as my indicator for *recent subordinate group protest*. The coefficient of *group-based concessions* (model 3) remains positive and statistically significant at the 90%-level even in the presence of a hypothetical confounder that is twice as strong as *recent subordinate protest*. Overall, this indicates that my results are not overly sensitive to violations of the exogeneity assumption. Specifically, an omitted confounder that is more (for *concessions*) or 2x (for *group-based concessions*) as strongly correlated with these independent variables and the outcome as *recent subordinate group protest* would be required to render my results non-significant at conventional statistical levels.

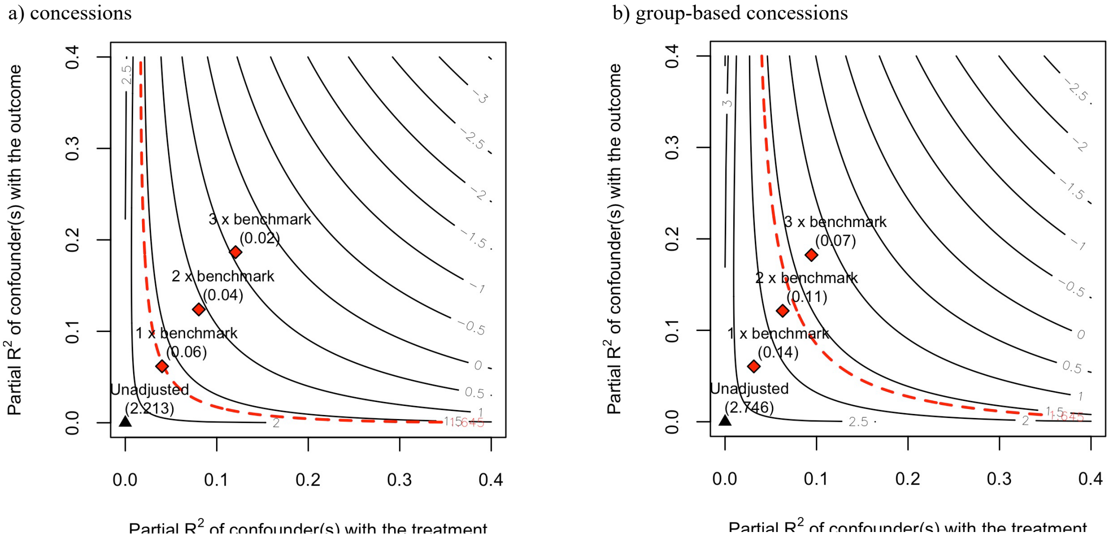

Figure A2. Causal sensitivity analyses (models 1 and 3). Benchmark confounder: *recent subordinate group protest*.

Appendix 1.3: Difference-in-differences approach

In a second step, I conduct a difference-in-differences estimation. This serves to probe whether I can replicate my findings using one of the most widely used and stringent quantitative approaches for approximating causal effects in observational settings; moreover, this approach allows me to more directly probe whether there is evidence for a pretrend, whereby patterns in dominant group mobilization might already shift before the three-month period around concessions which I examine.

Similar to my main analyses, this approach focuses squarely on within-country comparison between pre- and post-treatment periods, in my case corresponding to the three-month period around which concessions are granted. The identifying assumption is that, in the absence of treatment, treated groups (with concessions) and control groups (without concessions) would follow parallel trends over time. However, complicating the applicability of the standard two way fixed effects approach, recent work has shown that conventional estimators are potentially problematic under staggered treatment adaption, especially if the treatment can switch on and off (see Roth et al. 2023 for a review). This is a potential concern for my analysis, as many countries see repeated concessions to politically subordinate groups, and as I conceive of each concession as a time-limited treatment that will only affect dominant group mobilization during three-month time windows. To address these issues, I rely on the new fixed effects counterfactual (FECT) estimator, as proposed by Liu and colleagues (2024). The core idea is to use the control group, including not-yet treated units, to impute counterfactual outcomes for the treated units. Differences between observed and counterfactual outcomes can then be ascribed to the treatment(s), in this case to concessions.

As FECT requires a dichotomous treatment indicator, I dichotomize my independent variables, which take the value 1 if a concession of the respective type has been granted in each time period analyzed, regardless of the total number of such concessions. Figure A3 summarizes the results of this approach. Reassuringly, these results directly mirror my main findings: treated units (with concessions) see a substantively higher monthly number of dominant group mobilization events (+0.14 on average), especially if concessions are group-based (+0.24). In contrast, I find no statistically significant shift in dominant group mobilization around times when formally group-blind concessions are introduced. Moreover, though there are over-time shifts in dominant group mobilization, there is no visible pretrend towards more or less dominant group mobilization before the adoption of concessions, mirroring the results of appendix 1.1.

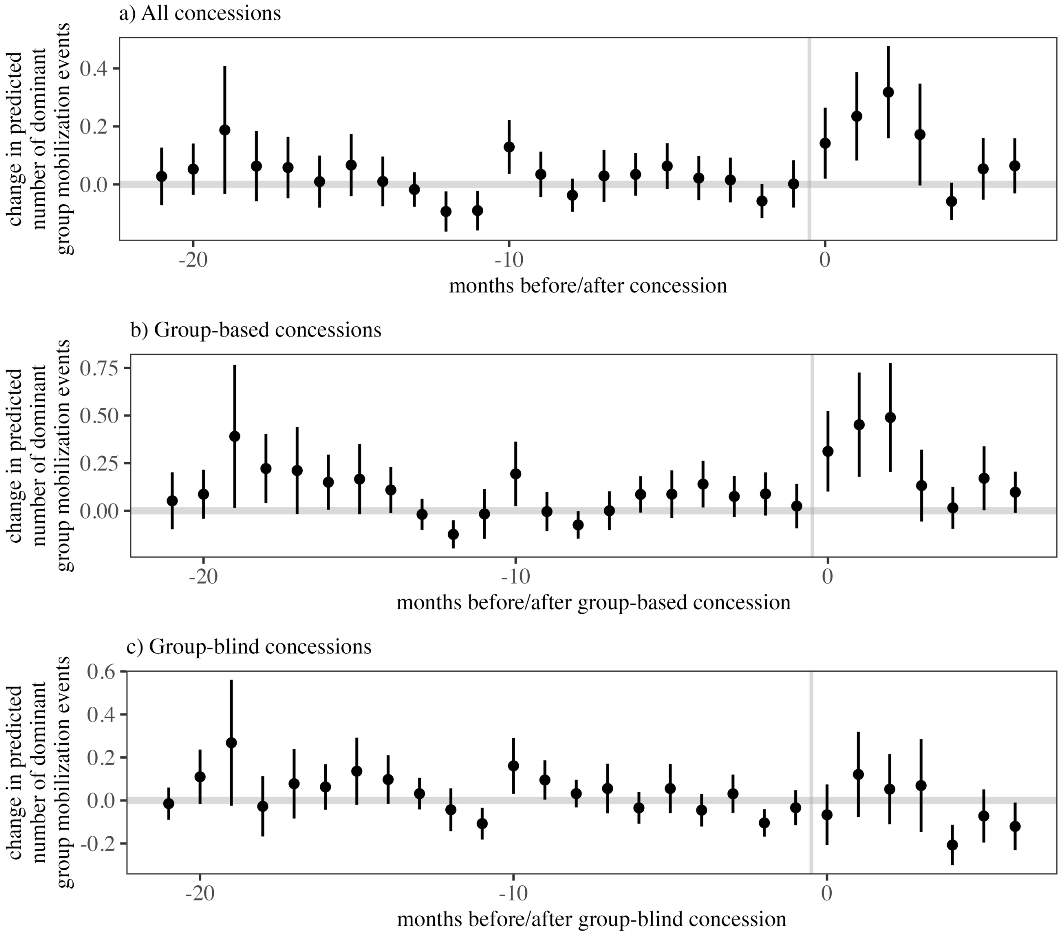

Figure A3. Fixed effects counterfactual (FECT) estimates: concessions and the number of dominant group mobilization events.

Appendix 1.4: Subsample analyses

In a third step, I re-run models 1 and 3 for split samples, distinguishing between contexts that have experienced ethnically based civil wars in the last ten years and those that have not, according to UCDP (Sundberg et al. 2012) and ACD2EPR (Vogt et al. 2015). Reassuringly, my main findings are replicated, with the estimated partial effects of concessions appearing comparable in both sub-samples, although the smaller sample diminishes the statistical significance levels in the post-conflict sample (see figure A4). This indicates that (group-based) concessions can also trigger dominant group mobilization in stable contexts without major preceding ethnic conflict, in line with my argument.

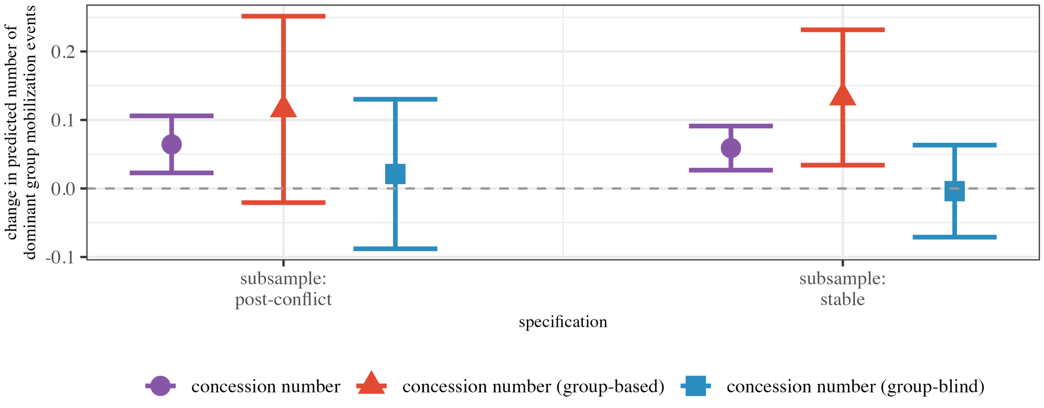

Figure A4. Partial effects and 90% confidence interval of *concession number*, *concession number (group-based)*, and *concession number (group-blind)* on the number of dominant group mobilization events for observed values in my sample [based on specifications that incorporate country year-fixed effects and subsample analyses focusing on post-conflict contexts (civil violence in the last 10 years) and stable contexts (no civil violence in the last 10 years)].

Appendix 2: Alternative explanations

Even if the attained relationships between ethnic accommodation and dominant group mobilization are robust to omitted variable bias, not subject to a detectable pretrend, and replicable in both conflict and stable contexts, they could be accounted for by alternative mechanisms. In this section, I summarize the results of a series of additional analyses that probe the potential of four alternative explanations to account for the observed empirical patterns:

- **Major constitutional and political shifts**: First, in line with hypothesis 1, I have found that months around (group-based) concessions are associated with a higher frequency of dominant group mobilization. However, rather than capturing a backlash against accommodation specifically, these results might reflect higher popular mobilization during periods of major constitutional and political shifts more generally. For instance, ethnic accommodation is often introduced or expanded following the signing of peace agreements, which might be contentious for reasons unrelated to accommodation. Similarly, shifts in the government's composition and constitutional amendments might increase mobilization, regardless of whether they empower ethnic subordinate groups. To address this possibility, I re-run my analyses while controlling for the promulgation of new constitutions, national-level elections, successful coups, peace agreements or cease fires, and changes in governing parties' social support base (appendix 2.1). Reassuringly, my results remain almost unchanged. This indicates that they are unlikely driven by general constitutional or political instability, rather than ethnic accommodation specifically.
- **Mobilization events unrelated to ethnic claims and opposition to accommodation**: Second, my dependent variables are coded based on the *ascriptive ethnic identities* of participants in the respective mobilization events, rather than their *public claims* to represent the dominant group and stated opposition to ethnic accommodation. This is in part because systematic information on such claims is hard to come by, even more difficult to code systematically, and was not systematically available for the underlying datasets on which the coding of my dependent variable is based (see data supplement for more details). However, focusing on ascriptive identities, instead of stated intent, also reflects the fact that members of dominant groups can conceivably mobilize in their own group's interest and oppose ethnic accommodation, but do so in the name of *individual* equality, as opposed to explicitly making ethno-centric claims. For instance, Bosniak nationalists have rallied against Bosnia's consociational system by referring to the limitations its ethnic quota and veto rights place on individual equality (Basta 2016). Nevertheless, for my analysis this coding entails the potential risk that my findings are strongly influenced by mobilization events that might have little connection to the grievances and fears of dominant groups that are at the center of my theoretical argument. This concern looms particularly large for anti-government protests that make up a large share of mobilization events in my sample (see figure 2) and contain a substantial number of protests that are unlikely to be directly related to ethnic accommodation. For instance, this applies to labor or environmental protests involving members of dominant groups. I probe whether this issue affects my findings by rerunning my analyses with an alternative dependent variable. This alternative dependent variable much more narrowly only incorporates anti-government protests that revolve around issues of institutional design and minority rights, while discarding the substantial number of protests that cannot be directly connected to such issues (appendix 2.2). Reassuringly, the attained empirical patterns are almost unchanged, reassuring me that my results are not primarily driven by mobilization events that have no direct relation to dominant groups' concerns about ethnic relations.
- **Violence initiated by subordinate groups:** Third, I have interpreted my results as evidence that concessions lend themselves to the articulation of injustice and fear frames, which in turn support mobilization efforts by majority nationalist parties. However, an important component of my dependent variable captures non-state violence between the ethnic majority and ethnic subordinate groups. Problematically, its underlying data does not enable me to identify which side initiated these violent incidents. Previous research suggests that ethnic subordinate groups are more likely to engage in civil violence to obtain political concessions, rather than communal violence against the dominant group (Hillesund 2019). Nevertheless, I cannot rule out that aspects of my dependent variable capture concession-seeking violence by ethnic subordinate groups. To address this concern, I rerun my models while exclusively focusing on anti-government protests (appendix 2.3). Reassuringly, my findings remain similar (though with reduced statistical significance), indicating that they are not driven by violence initiated by subordinate groups.
- **Elite opposition to restrictive ethnically based power-sharing:** Fourth, I have found that group-based concessions are associated with a disproportionate degree of dominant group mobilization. These findings are based on a purposefully broad operationalization of concessions, which encompasses consociationalism, power-sharing, regional autonomy, and multiculturalism (cf. McGarry, O'Leary & Simeon 2008). While this broad operationalization best captures my argument, it entails challenges for the interpretation of my results. An important concern is that dominant group mobilization might be driven by elite opposition against rigid, ethnically based power-sharing institutions---which circumscribe elite dominance at the center---, rather than by mass grievances and fears. For example, facing the new constraints of the Arusha power-sharing agreement, Hutu hardliners created militias, took over power by force, and engaged in systematic killings of Hutu moderates

and the Tutsi minority (Straus 2006: 44). To examine this alternative explanation, I rerun separate models that distinguish between horizontal and vertical concessions (appendix 2.3). Although these models provide further nuance, I attain similar patterns for both dimensions. This suggests that even overwhelmingly symbolic concessions that do not restrict dominant group elites' ability to dominate central government policy, such as the recognition of subordinate groups' cultural practices, can generate dominant group mobilization, in line with my argument.

In the following pages, I explain these procedures in more detail and report the average partial effects attained in them, derived for the observed values in the sample. The supplementary R-script generates tables that contain the full model results.

Appendix 2.1: Major constitutional and political shifts

In a first step, I examine whether my findings could be explained by major constitutional and political shifts that often precede or accompany the provision of ethnic accommodation. For this purpose, I identify months during which:

- new constitutional systems are adopted, based on the Comparative Constitutions Project (CCP, Elkins et al. 2014);[1]
- national executive or legislative elections are held, based on NELDA (Hyde & Marinov 2012);
- successful coups occur (Marshall & Ramsey Marshall 2022);
- peace agreements or ceasefires are signed, based on the UCDP Conflict Termination dataset (Kreutz 2010);
- the government's social support groups are subject to change, based on the V-Parties dataset (Lindberg et al. 2022).[2]

I control for these major political and constitutional shifts by incorporating five separate control variables. I construct these analogously to my main independent variables (group-based/group-blind concessions) as dichotomous terms that take the value 1 in the three-month windows before and after these shifts, and 0 otherwise. Reassuringly, my results remain almost unchanged when controlling for this expansive set of major constitutional and political shifts that often coincide with concessions (see figure A5). This indicates that my results are unlikely driven by general constitutional or political instability.

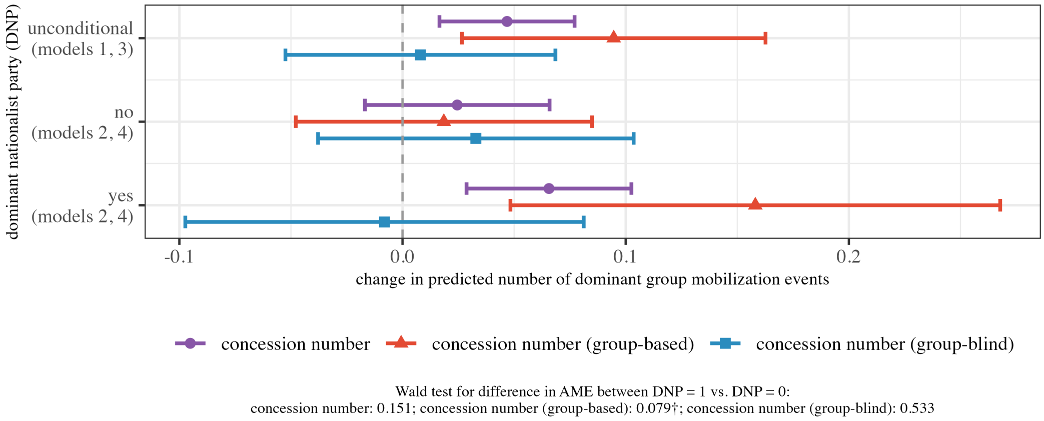

Figure A5. Partial effects and 90% confidence interval of *concession number*, *concession number (group-based)*, and *concession number (group-blind)* on the number of dominant group mobilization events for observed values in my sample [based on specifications that control for three-month windows around the adoption of new constitutions, national-level elections, coups, the signing of peace treaties and cease-fires, and changes in the government's support group].

Appendix 2.2: Mobilization unrelated to ethnic issues

In a second step, I probe whether mobilization unrelated to ethnic issues may affect my findings. A particular concern are anti-government protests involving dominant group members, many of which may have little relation to dominant groups' grievances and fears sparked by ethnic accommodation. For instance, this applies to labor or environmental protests involving members of dominant groups. To address this issue, I identify protests that are related to questions of institutional design and minority rights. To identify such protests, a research assistant systematically screened the text-based information provided by MMD (Clark and Regan 2016), in particular each protester group's coded identity and the

[1] For each new constitutional system coded by CCP for my sampled country months, I identify the year, month and day that it was first introduced.

[2] Using the V-Parties dataset (Lindberg et al. 2022), I identify the support group(s) of all governing parties. I code changes in the government's social support group in each month where the composition of any governing party's support groups changes. The underlying variable from this dataset, *v2pagroup*, provides information on whether each party's support groups include the aristocracy, agrarian elites, business elites, the military, an ethnic or racial group, a religious group, local elites, urban working classes, urban middle classes, rural working classes, rural middle classes, regional groups, or women.

short newspaper article extracts, both of which MMD provides for each protest event. Using this information, the research assistant identified all protests involving members of dominant groups that were related to:

- **Government composition:** The protesters demand changes in government composition or the institutions that determine this. This could refer to demands that the government should be exclusively or dominantly composed of dominant group members, that specific subordinate groups be excluded, or that a power-sharing agreement be overturned. Conversely, this does *not* include the simple expression of a political party's intention to achieve its inclusion into the executive; rather, a specific ethnic claim is required. For example, in Macedonia in 2001 there was a significant protest movement against the inclusion of an Albanian minority party into government, with nationalist parties demanding the restoration of ethnic Macedonian dominance in the cabinet.
- **Regional government:** The protesters demand changes in regional government status, including its authority or boundaries. This could refer to demands that an autonomous region for the dominant group be established, that the existing autonomy of a subordinate group be withdrawn, or that the empowerment of subordinate groups through federalization or decentralization should be prevented or revoked. In the latter case, an explicitly ethnic or cultural link is required; for example, demands by small US American organizations for more state autonomy or, respectively, against federalism are not considered. Conversely, the 2004 Macedonian protests against decentralization are considered, as these were based on explicitly-formulated fears that decentralization would empower the Albanian minority and enable secessionism.
- **Culture:** The protesters demand changes in state-endorsed or allowed cultural rights or practices. This could refer to demands for the constitutional recognition of the dominant group's culture, including religion or language, demands against the recognition of subordinate groups' culture, or against specific subordinate groups' cultural practices. For example, the Indian Hindu nationalist movement has made several such claims: For the constitutional entrenchment of India as a Hindu nation, against formal Muslim cultural rights, against movies which violate Hindu morals, and against cattle slaughter, which is predominantly practiced by Indian Muslims.
- **War termination:** The protesters demand that the settlement of a previous armed conflict be overturned or a proposed settlement be prevented. For example, this might refer to mobilization against peace agreements, as occurred in Macedonia (2001) and Rwanda (1994). Mobilization against the specific terms of a peace agreement is additionally coded in the more specific categories provided above (e.g. in the regional government variable, if an EMM is directed against ethnic minority autonomy foreseen in a peace agreement).

Using this coding, I construct an alternative dependent variable that omits all protests which are not related to one of these issues above. Reassuringly, my main findings remain replicable when employing this alternative dependent variable (see figure A6) which substantially reduces the average number of dominant group mobilization events (from a total of 12'371 events using the original measure to 2237 events using this more restrictive measure). This indicates that my results are not primarily driven by mobilization events that have no relation to dominant groups' grievances and fears over ethnic accommodation.

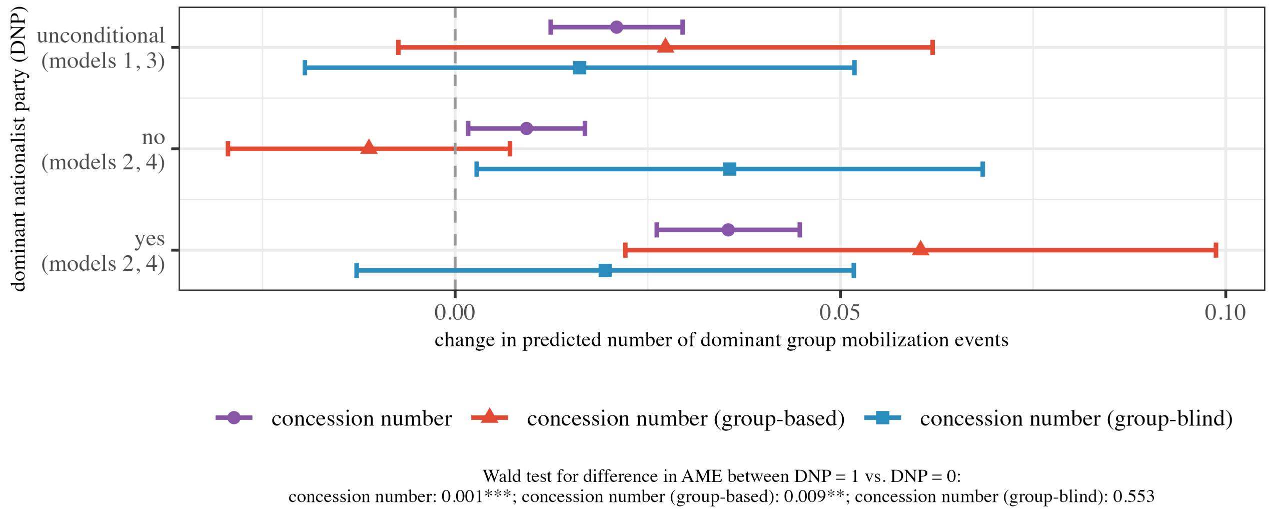

Figure A6. Partial effects and 90% confidence interval of *concession number*, *concession number (group-based)*, and *concession number (group-blind)* on the number of dominant group mobilization events for observed values in my sample [alternative dependent variable that only counts protest events related to the state's institutional design and minority rights].

Appendix 2.3: Violence initiated by subordinate groups

In a third step, I probe whether concession-seeking, minority-initiated violence might explain my findings. For this purpose, I use a narrower dependent variable that exclusively counts the number of anti-government protests in each month. Conversely, I omit my original dependent variable's targeted violence component, for which I am unable to ascertain which side initiated the violence. Reassuringly, my findings remain similar, although, owing to the much reduced variance in the dependent variable, with lower statistical significance for group-based concessions, indicating that they are not predominantly driven by violence initiated by subordinate groups (see figure A7).

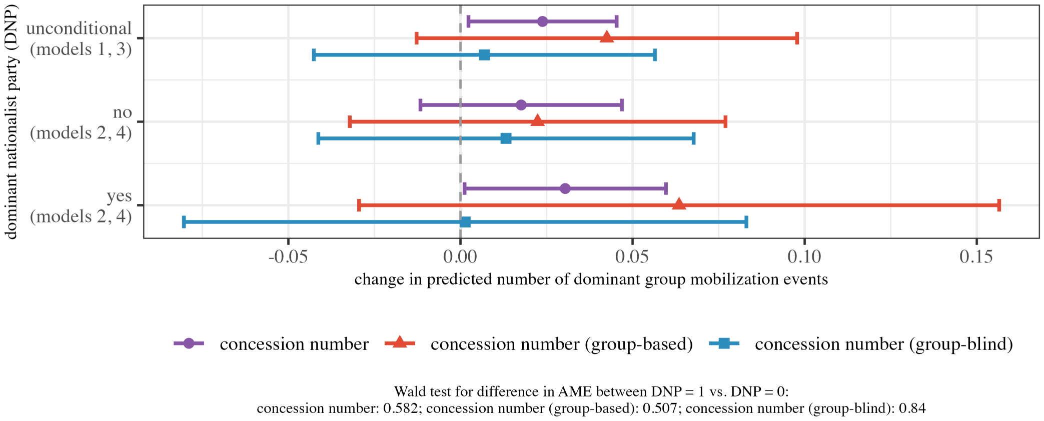

Figure A7. Partial effects and 90% confidence interval of concession number, concession number (group-based), and concession number (group-blind) on the number of dominant group protests for observed values in my sample.

Appendix 2.4: Elite opposition to restrictive, ethnically based power-sharing

Finally, in a fourth step, I probe whether elite opposition to restrictive, ethnically based power-sharing might explain the attained empirical patterns, as opposed to grievances and fears among the wider majority community. For this purpose, I rerun my analyses while splitting up my independent variables: in a first specification, I exclusively focus on horizontal concessions (power-sharing and consociationalism); in a second, I conversely only focus on vertical concessions (regional autonomy and recognition of minority cultural practices).

If elite opposition, rather than changes in mass attitudes, explained the attained relationship between concessions and dominant group mobilization, I would expect to find increased dominant group mobilization around times when ethnically-based power-sharing provisions are introduced (the horizontal component of my original concessions variable). Conversely, I would expect less pronounced patterns following more limited, symbolic measures that do not directly constrain governing elites, such as the recognition of subordinate groups' cultural practices (the vertical component of my original concessions variable). Reassuringly, while these separate specifications provide further nuance (see figures A8 and A9), I attain similar patterns for both dimensions; indeed if anything, the empirical patterns are more pronounced for the "soft" concessions entailed by the recognition of subordinate groups' collective identities and their cultural characteristics, which make up my group-based vertical concessions variable. This suggests that even overwhelmingly symbolic concessions may generate dominant group mobilization, in line with my argument. Conversely, it makes it less likely that the attained empirical patterns can be fully explained by the instrumental actions of nationalist or opportunistic elites.

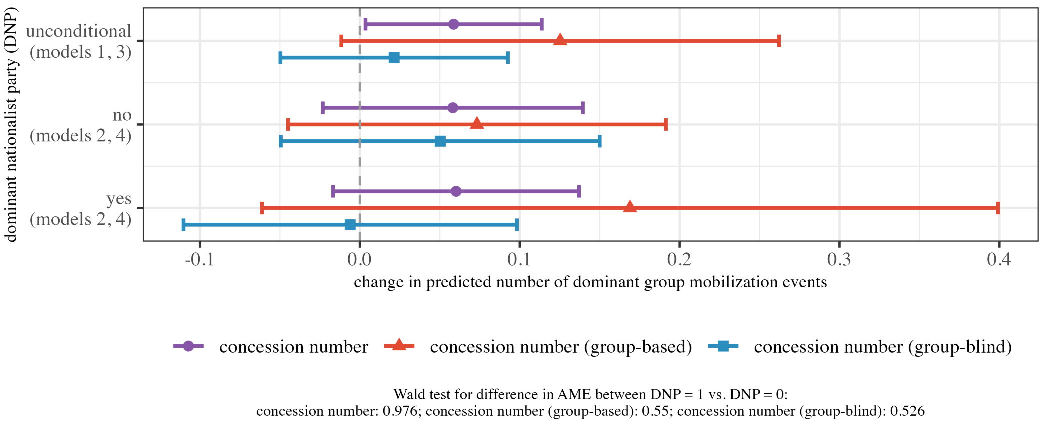

Figure A8. Partial effects and 90% confidence interval of *horizontal concessions*, *horizontal group-based concessions*, and *horizontal group-blind concessions* on the number of dominant group mobilization events for observed values in my sample.

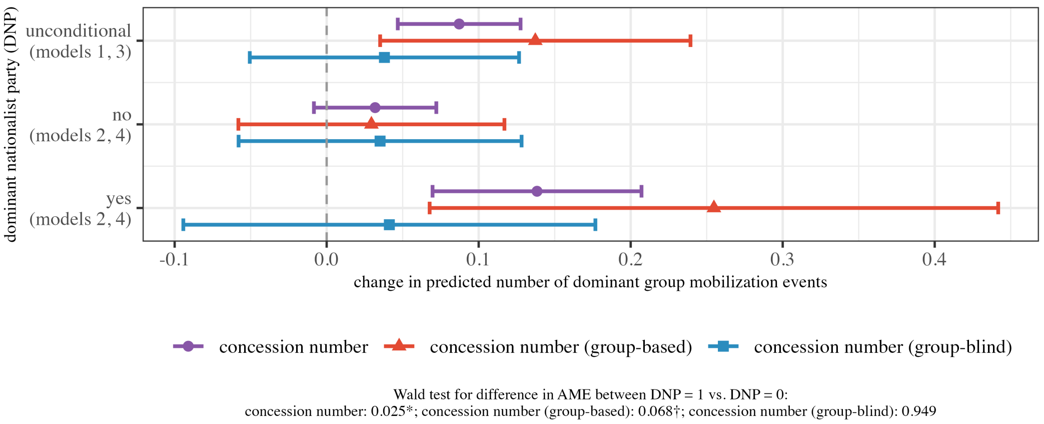

Figure A9. Partial effects and 90% confidence interval of *vertical concessions*, *vertical group-based concessions*, and *vertical group-blind concessions* on the number of dominant group mobilization events for observed values in my sample.

Appendix 3: General robustness checks

Finally, I conduct a series of general robustness checks. I sequentially examine how my results are affected by alterations to the independent variables (appendix 3.1), dependent variable (appendix 3.2), sample (appendix 3.3), the selection of control variables (appendix 3.4), and specification (appendix 3.5). Again, I report the average partial effects attained in these procedures, derived for the observed values in the sample, while the supplementary R-script generates tables that contain the full model results.

Appendix 3.1: Alterations to independent variables

In a first step, I probe how alternative operationalizations of my independent variables, *concession number*, *concession number (group-based)*, and *concession number (group-blind),* affect my results. I first recode these variables, while replacing the 3-month time window used in my main models with alternative time windows of 1, 2, 6, and 12 months (appendix 3.1.1). The more fine-grained time windows (1 and 2 months) only pick up short-term variation immediately before and after the introduction of concessions. Conversely, the wider time windows (6 and 12 months) account for the fact that constitutional amendments, on which a large share of identified concessions are based, are often introduced into the political process, and hence become subject to debate, well before they are promulgated. Second, instead of counting the number of concessions within a three-month time window, I consider whether any concession of the respective type has been given in the respective time window. For this purpose, I employ a dichotomous variable that indicates whether this is the case (appendix 3.1.2). Third, I implement an alternative operationalization of my original concession variables that only counts concessions in the preceding three months (appendix 3.1.3). Thereby, this operationalization does not pick up dominant group mobilization in the lead-up to concessions, which might influence their eventual adoption (see main article text and appendix 1.1). Reassuringly, my results remain almost unchanged in any of these procedures. This indicates that they are not sensitive to the specific way in which I operationalize concessions that provide for ethnic accommodation.

Finally (appendix 3.1.4), I probe the robustness of my interacted variable, the existence of a *dominant nationalist party* (DNP). Instead of employing a dichotomous indicator, I use a continuous variable that counts the (logged) number of dominant nationalist parties. Again, I provide Wald tests that calculate the significance in differences in marginal effects, depending on the number of dominant nationalist parties.[3] This points to significant differences between constellations where no dominant nationalist party exists versus constellations where at least one or multiple such parties exist. In contrast, higher-order differences (e.g. between three and four) such parties are not significant. This bolsters the approach chosen in my main analyses, which differentiates between constellations with and without dominant nationalist parties in a dichotomous manner.

Appendix 3.1.1: Different time windows

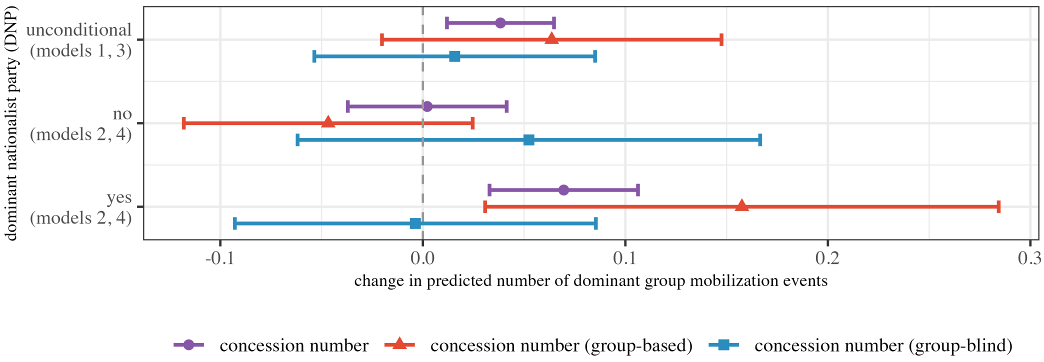

Wald test for difference in AME between DNP = 1 vs. DNP = 0:
concession number: 0.044*; concession number (group-based): 0.02*; concession number (group-blind): 0.534

Figure A10. Partial effects and 90% confidence interval of concession number, concession number (group-based), and concession number (group-blind) on the number of dominant group mobilization events for observed values in my sample [based on specifications that capture 1-month time windows around the provision of concessions].

[3] The maximum number is 11; For simplicity's sake, I only report results for the existence of 0 to 5 parties, omitting the small number of cases where more such parties concurrently exist and compete for office.

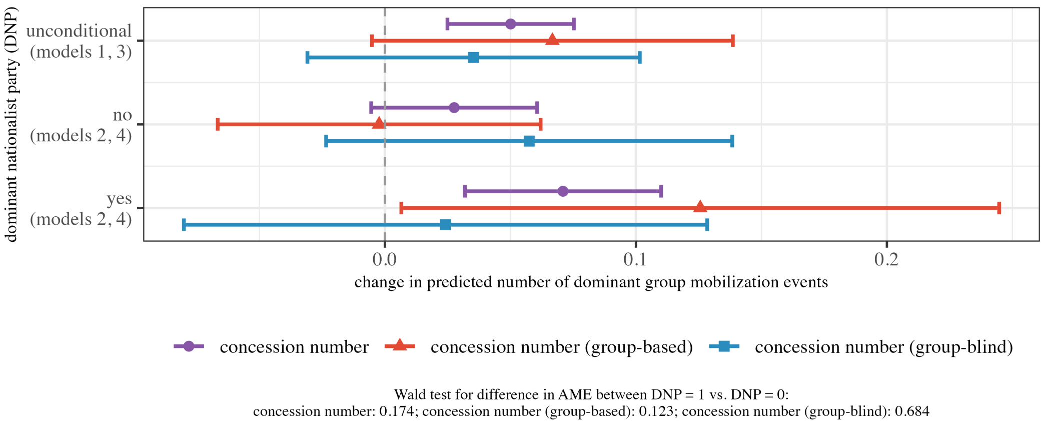

Figure A11. Partial effects and 90% confidence interval of concession number, concession number (group-based), and concession number (group-blind) on the number of dominant group mobilization events for observed values in my sample [based on specifications that capture 2-month time windows around the provision of concessions].

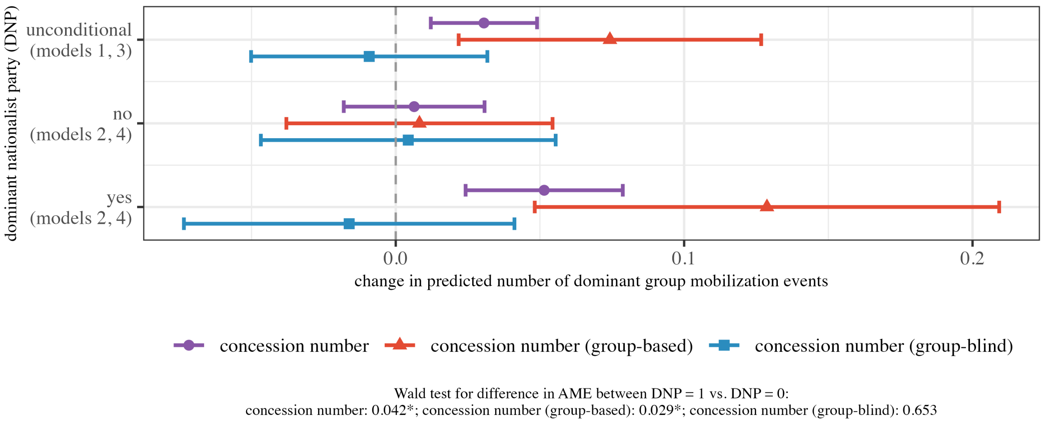

Figure A12. Partial effects and 90% confidence interval of concession number, concession number (group-based), and concession number (group-blind) on the number of dominant group mobilization events for observed values in my sample [based on specifications that capture 6-month time windows around the provision of concessions].

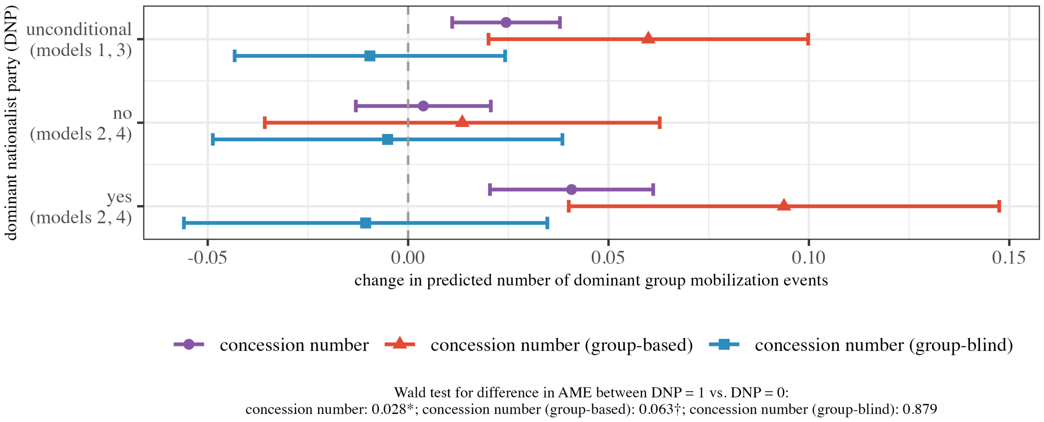

Figure A13. Partial effects and 90% confidence interval of concession number, concession number (group-based), and concession number (group-blind) on the number of dominant group mobilization events for observed values in my sample [based on specifications that capture 12-month time windows around the provision of concessions].

Appendix 3.1.2: Dichotomous concession indicator

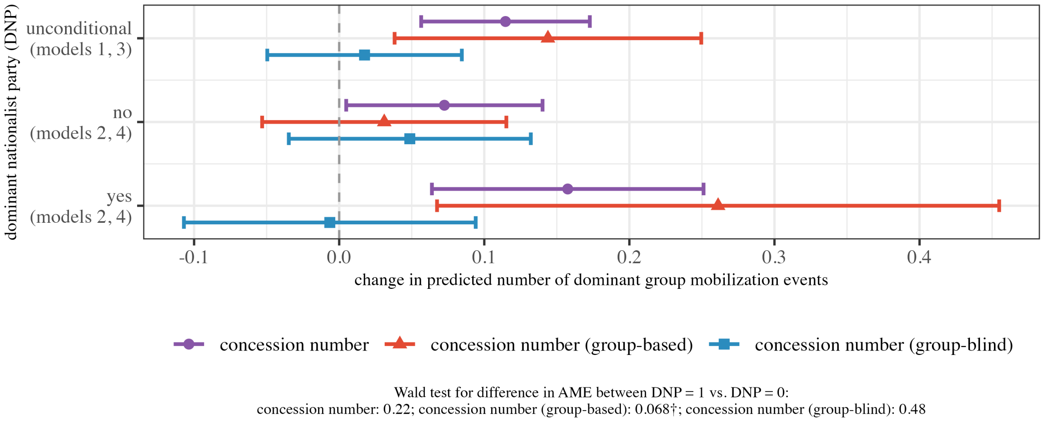

Figure A14. Partial effects and 90% confidence interval of concession number, concession number (group-based), and concession number (group-blind) on the number of dominant group mobilization events for observed values in my sample [based on specifications that operationalize concessions as a dichotomous variable].

Appendix 3.1.3: Only concessions in the past three months

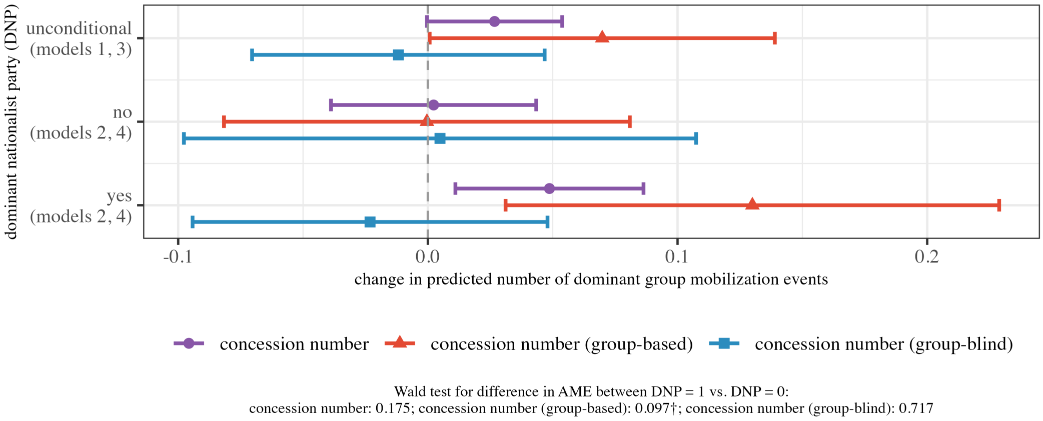

Figure A15. Partial effects and 90% confidence interval of concession number, concession number (group-based), and concession number (group-blind) on the number of dominant group mobilization events for observed values in my sample [based on specifications that capture a 3-month time window after the provision of concessions].

Appendix 3.1.4: Continuous measure for the logged number of dominant nationalist parties

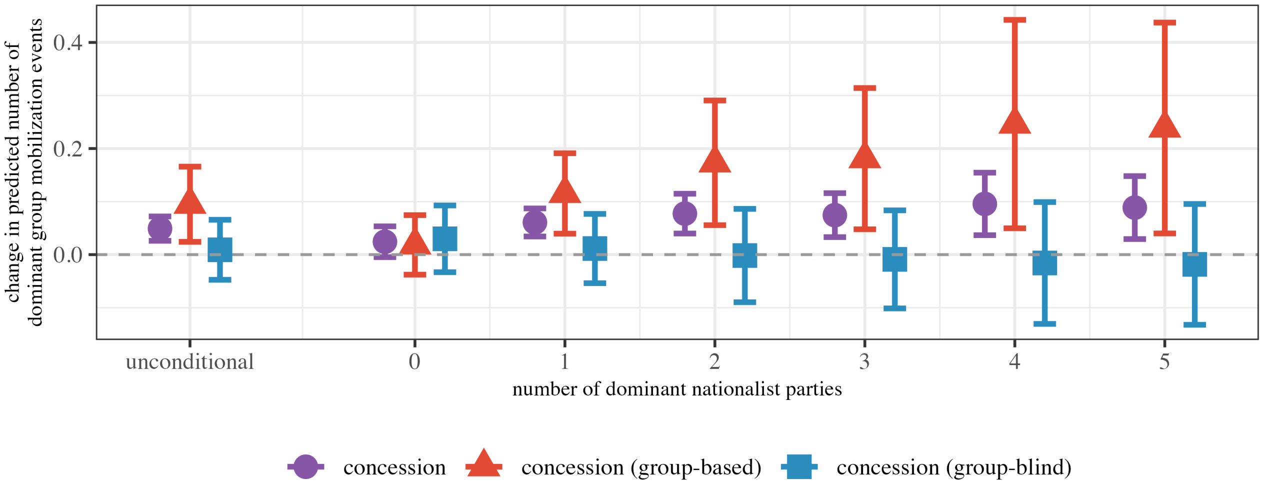

Figure A16. Partial effects and 90% confidence interval of concession number, concession number (group-based), and concession number (group-blind) on the number of dominant group mobilization events for observed values in my sample [interaction with the logged number of dominant nationalist parties].

Table A2. Wald tests for statistical significance in difference of partial effects of concessions, group-based concessions, and group-blind concessions, depending on the logged number of dominant nationalist parties (DNPs).

Comparison (number of DNPs)	Concession number	Concession number (group-based)	Concession number (group-blind)
0-1	-0.036 (0.018)*	-0.097 (0.043)*	0.018 (0.042)
0-2	-0.053 (0.03)†	-0.155 (0.077)*	0.032 (0.065)
0-3	-0.05 (0.034)	-0.163 (0.09)†	0.039 (0.072)
0-4	-0.071 (0.045)	-0.228 (0.13)†	0.046 (0.086)
0-5	-0.064 (0.046)	-0.22 (0.132)†	0.048 (0.087)
0-6	-0.14 (0.078)†	-0.434 (0.254)†	0.067 (0.141)
0-7	-0.162 (0.084)†	-0.494 (0.28)†	0.072 (0.151)
0-8	-0.095 (0.063)	-0.341 (0.222)	0.06 (0.11)
0-9	-0.061 (0.051)	-0.245 (0.176)	0.053 (0.086)
0-10	-0.123 (0.085)	-0.462 (0.337)	0.073 (0.132)
1-2	-0.017 (0.012)	-0.058 (0.034)†	0.013 (0.023)
1-3	-0.014 (0.017)	-0.066 (0.048)	0.02 (0.031)
1-4	-0.035 (0.028)	-0.131 (0.087)	0.027 (0.045)
1-5	-0.028 (0.029)	-0.123 (0.09)	0.03 (0.046)
1-6	-0.104 (0.061)†	-0.337 (0.211)	0.048 (0.099)
1-7	-0.126 (0.067)†	-0.397 (0.237)†	0.054 (0.11)
1-8	-0.059 (0.046)	-0.244 (0.179)	0.042 (0.069)
1-9	-0.024 (0.035)	-0.149 (0.134)	0.034 (0.047)
1-10	-0.086 (0.068)	-0.365 (0.295)	0.054 (0.091)
2-3	0.003 (0.005)	-0.008 (0.015)	0.007 (0.009)
2-4	-0.018 (0.016)	-0.073 (0.053)	0.014 (0.022)
2-5	-0.011 (0.017)	-0.066 (0.057)	0.017 (0.023)
2-6	-0.087 (0.05)†	-0.279 (0.178)	0.035 (0.076)
2-7	-0.109 (0.055)*	-0.339 (0.204)†	0.041 (0.087)
2-8	-0.042 (0.034)	-0.186 (0.146)	0.029 (0.046)
2-9	-0.008 (0.025)	-0.091 (0.102)	0.021 (0.025)
2-10	-0.07 (0.057)	-0.307 (0.262)	0.041 (0.068)
3-4	-0.021 (0.012)†	-0.065 (0.04)	0.007 (0.014)
3-5	-0.014 (0.013)	-0.058 (0.043)	0.009 (0.015)
3-6	-0.09 (0.046)*	-0.272 (0.166)	0.028 (0.071)
3-7	-0.112 (0.051)*	-0.331 (0.191)†	0.034 (0.081)
3-8	-0.045 (0.03)	-0.178 (0.133)	0.021 (0.038)
3-9	-0.011 (0.021)	-0.083 (0.09)	0.014 (0.016)
3-10	-0.072 (0.052)	-0.299 (0.249)	0.034 (0.061)
4-5	0.007 (0.004)	0.007 (0.013)	0.003 (0.003)
4-6	-0.069 (0.035)*	-0.206 (0.128)	0.021 (0.056)
4-7	-0.091 (0.041)*	-0.266 (0.152)†	0.027 (0.067)
4-8	-0.024 (0.019)	-0.113 (0.095)	0.015 (0.024)
4-9	0.011 (0.014)	-0.018 (0.056)	0.007 (0.01)
4-10	-0.051 (0.042)	-0.234 (0.211)	0.027 (0.046)
5-6	-0.076 (0.035)*	-0.214 (0.126)†	0.019 (0.057)
5-7	-0.098 (0.041)*	-0.273 (0.152)†	0.024 (0.067)
5-8	-0.031 (0.02)	-0.12 (0.095)	0.012 (0.023)
5-9	0.004 (0.015)	-0.025 (0.058)	0.004 (0.007)
5-10	-0.058 (0.042)	-0.241 (0.211)	0.024 (0.046)
6-7	-0.022 (0.014)	-0.06 (0.044)	0.006 (0.011)
6-8	0.045 (0.021)*	0.093 (0.051)†	-0.006 (0.034)
6-9	0.079 (0.033)*	0.189 (0.093)*	-0.014 (0.06)
6-10	0.018 (0.02)	-0.028 (0.101)	0.006 (0.017)
7-8	0.067 (0.024)**	0.153 (0.063)*	-0.012 (0.044)
7-9	0.101 (0.038)**	0.248 (0.112)*	-0.02 (0.069)
7-10	0.04 (0.018)*	0.032 (0.074)	0 (0.024)
8-9	0.035 (0.018)†	0.095 (0.061)	-0.008 (0.026)
8-10	-0.027 (0.026)	-0.121 (0.122)	0.012 (0.023)
9-10	-0.062 (0.037)†	-0.216 (0.168)	0.02 (0.047)

Appendix 3.2: Alterations to dependent variable (dominant group mobilization)

In a second step, I probe how alternative operationalizations of my dependent variable, the *number of dominant group mobilization events*, affect my findings. I first employ a narrower operationalization thereof that exclusively focuses on

major mobilization events: protests that involve at least 100 participants and violent incidents that individually result in at least 5 casualties (appendix 3.2.1). This serves to account for possible media reporting bias, whereby small-scale protests or less fatal violent incidents might not be consistently reported in the media sources underlying my data. In contrast, the data quality should be more consistent for these larger-scale events.

Second and third, I run separate specifications for violent (violent anti-government protests and violence directed against subordinate groups) and non-violent dominant group mobilization events (non-violent anti-government protests) (appendices 3.2.2 and 3.2.3). This serves to probe where the attained empirical patterns "come from" and ascertain that they apply to both violent and non-violent forms of mobilization, both of which are covered by my argument.

By focusing on subsets of my original dependent variable, all these alternative operationalizations substantially reduce the variance available for my analyses. Though this reduces the statistical significance of the partial effects attained in these procedures (see figures A17-A19), the attained empirical patterns remain very similar to those of my main analyses. All in all, this reassures me that the specific way I have operationalized my dependent variable does not critically affect my findings.

Appendix 3.2.1: Only major dominant group mobilization events

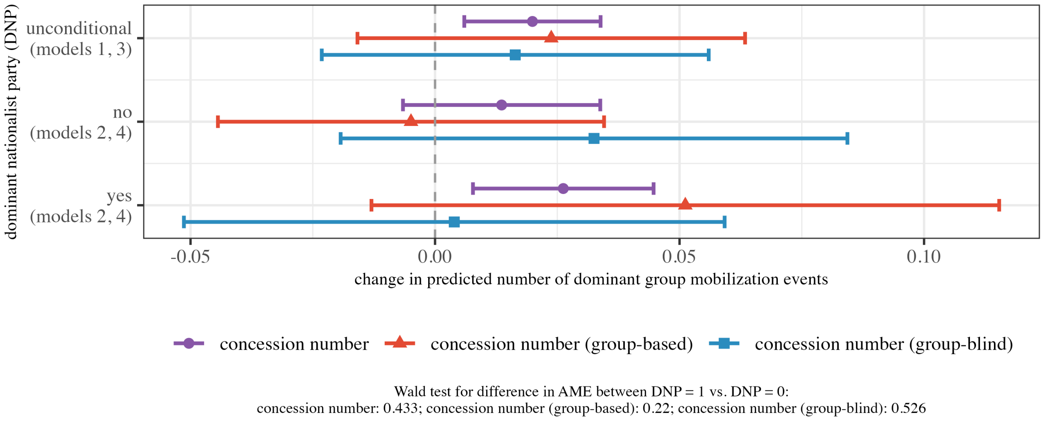

Figure A17. Partial effects and 90% confidence interval of concession number, concession number (group-based), and concession number (group-blind) on the number of major dominant group mobilization events for observed values in my sample [based on specifications that exclusively capture anti-government protests with at least 100 participants and anti-minority violent incidents with at least 5 fatalities].

Appendix 3.2.2: Only violent dominant group mobilization events

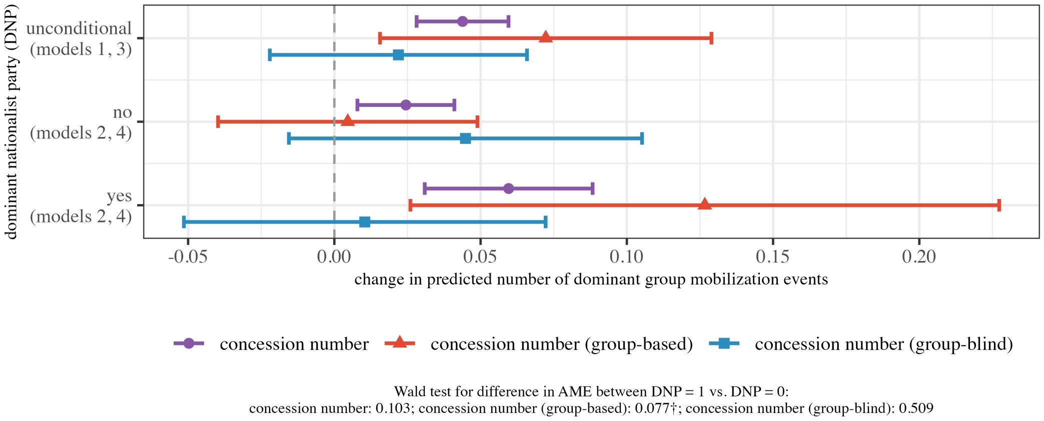

Figure A18. Partial effects and 90% confidence interval of concession number, concession number (group-based), and concession number (group-blind) on the number of violent dominant group mobilization events for observed values in my sample [based on specifications that exclusively capture violent anti-government protests and anti-minority violence].

Appendix 3.2.3: Only non-violent group mobilization events

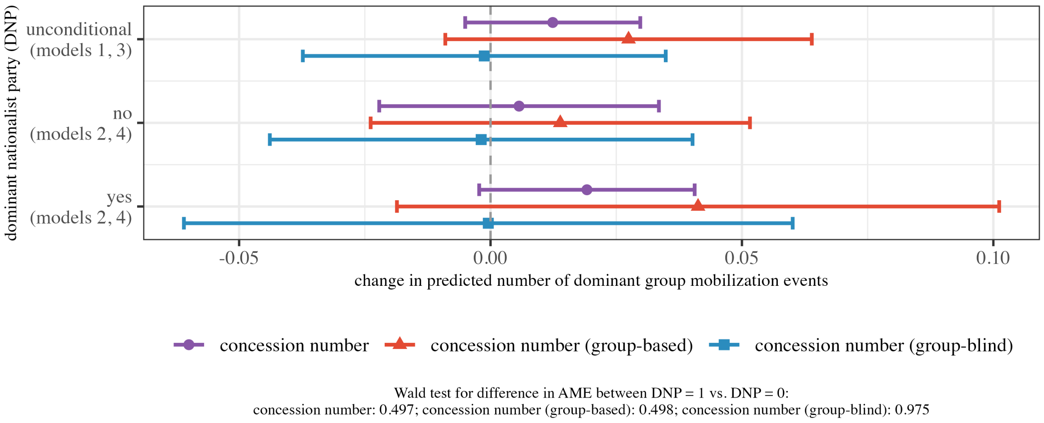

Figure A19. Partial effects and 90% confidence interval of concession number, concession number (group-based), and concession number (group-blind) on the number of non-violent dominant group mobilization events for observed values in my sample [based on specifications that exclusively capture non-violent anti-government protests].

Appendix 3.3: Sample alterations

In a third step, I examine the robustness of my findings to alterations of my sample. I start by probing whether the way I identify politically dominant groups influences my results. First, instead of limiting my sample to those ethnic groups that are *currently* politically dominant, I include all groups that have held the politically most influential position in a country at any point in the last 12 months (appendix 3.3.1). This enables me to incorporate groups who became downgraded from political dominance as a *result* of concessions that provide for ethnic accommodation. For instance, this applies to the Afrikaners in South Africa following the end of Apartheid in 1993-1994, who do not classify as the politically dominant group thereafter, yet still protested against the new power-sharing constitution. Second, I adopt an alternative definition of the ethnic dominant group that is based exclusively on demographic criteria, by limiting the sample to those groups that make up a plurality of the population (appendix 3.3.2). Reassuringly, neither of these alterations affect my main findings.

Next, I probe whether the way I identify electoral regimes shapes my findings. In my main analyses I had not only included democracies, but also electoral autocracies and cases that are undergoing democratic transitions, according to the V-Dem Episodes of Regime Transition dataset (Maerz et al. 2021). To examine whether the inclusion of electoral autocracies and democratizing countries, which might see a disproportionate amount of nationalist mobilization (Snyder 2000), drives my findings, I exclude these from my sample and focus only on electoral and liberal democracies (appendix 3.3.3). Reassuringly, my findings remain almost identical in this more narrowly defined sample.

Appendix 3.3.1: Any group that has been politically-dominant in the last 12 months

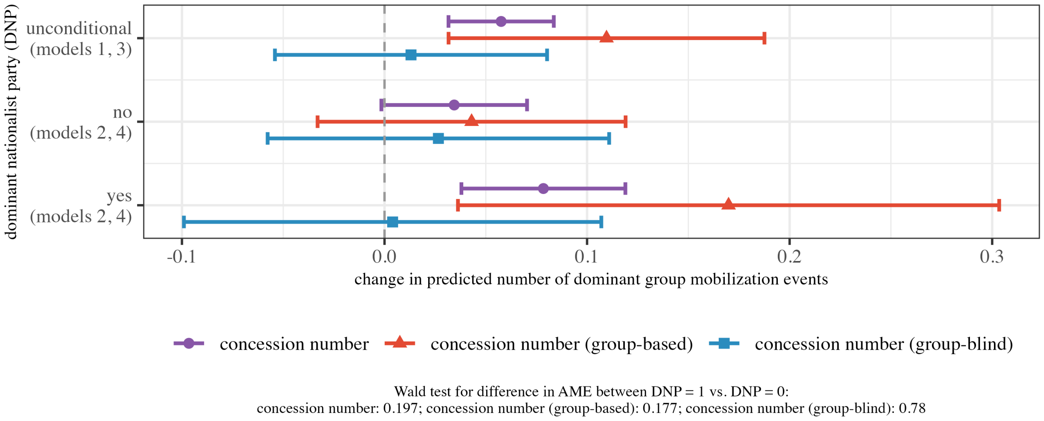

Figure A20. Partial effects and 90% confidence interval of concession number, concession number (group-based), and concession number (group-blind) on the number of dominant group mobilization events for observed values in my sample [sample: any ethnic group that has been politically-dominant in the past 12 months].

Appendix 3.3.2: Only demographic plurality groups

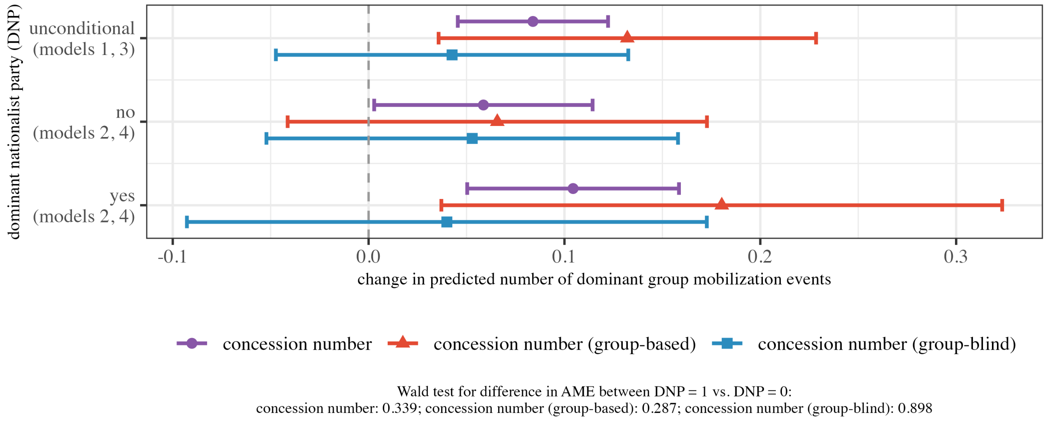

Figure A21. Partial effects and 90% confidence interval of concession number, concession number (group-based), and concession number (group-blind) on the number of dominant group mobilization events for observed values in my sample [sample: only ethnic groups that form a demographic plurality of the population].

Appendix 3.3.3: Only electoral and liberal democracies

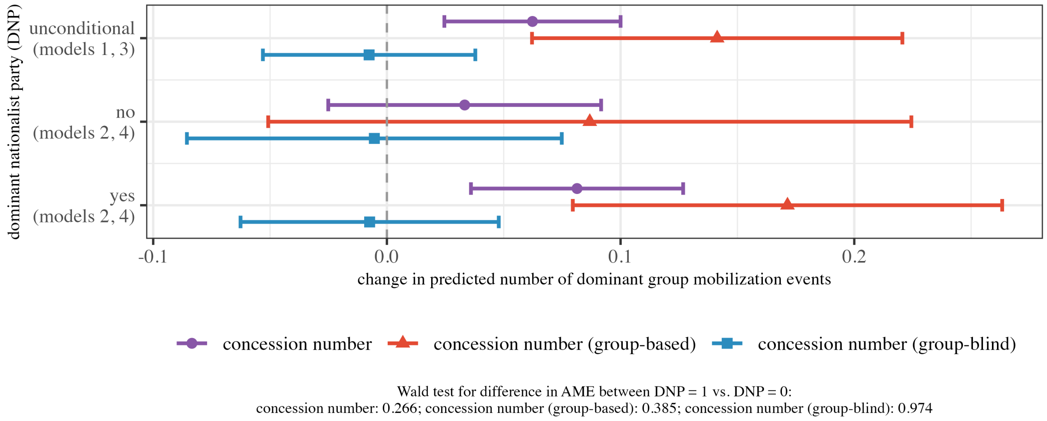

Figure A22. Partial effects and 90% confidence interval of concession number, concession number (group-based), and concession number (group-blind) on the number of dominant group mobilization events for observed values in my sample [sample: only electoral and liberal democracies, excluding electoral autocracies and democratizing states].

Appendix 3.4: Incorporation of additional control variables

In a fourth step, I probe whether the incorporation of additional control variables affects my results. In a first specification (appendix 3.4.1), I control for additional structural characteristics which might facilitate dominant group mobilization by increasing its supply among the dominant group membership: years during which a country is undergoing democratizing and autocratizing regime transitions (Maerz et al. 2021), each country's annual unemployment rate, and each country's annual GINI index (both from World Bank 2020).

In another specification (appendix 3.4.2), I instead control for a series of factors that may critically constrain bargaining between the government and subordinate groups and that might simultaneously shape the incidence of a backlash against ethnic accommodation:

- First, I include a dichotomous variable indicating whether any subordinate group in the state is subject to irredentist claims from a kin state government (Cederman et al., 2022).
- Second, I control for the (logged) number of self-determination movements involving subordinate groups in a given country year, using data from the SDM dataset (Sambanis, Germann & Schädel, 2018), updated with Cederman et al. (2022). Both these factors might increase the probability that concessions will be provided to subordinate groups; however, at the same time, they might also *themselves* engender a backlash (e.g., against contentious demands by subordinate groups or against third-state "meddling"), irrespective of whether they result in concessions.
- Third, I include a control that captures the difference in relative size between the dominant and the largest subordinate group, calculated with the EPR *size* variable (Vogt et al. 2015). Higher positive values of this variable designate situations in which the dominant group is clearly dominant in demographic terms. Conversely, negative values capture the inverse situation in which the dominant group is demographically

smaller than subordinate groups (e.g., the Afrikaners in apartheid-South Africa). Demographically larger dominant groups are less likely to come under pressure to make substantial concessions to subordinate groups; however, their membership may also be more generous and less likely to feel threatened by concessions, owing to their group's assured role as the numerically dominant "Staatsvolk" (o'Leary 2002).

- Fourth, I control for the (logged) number of years that the dominant group has been politically dominant according to EPR (Vogt et al. 2015). Following long periods of political dominance, dominant group elites may be able to successfully fend off subordinate groups' demands for concessions. At the same time, dominant groups that are used to dominating the political system may take a "proprietary view" of their overrepresentation and be more likely to rally against concessions that circumscribe their customary dominance (Rothchild 1981: 217; cf. Cederman et al. 2013: 182).

In a final check (appendix 3.4.3), I control for factors that may shape incentives of dominant nationalist parties to organize anti-government protests or incite anti-minority violence, irrespective of concessions. For this purpose I interact my term for the existence of a *dominant nationalist party* with three additional terms: (1) *months to next election (log)*, already used as a control in my main models, (2) a dichotomous variable designating democratic and autocratic *regime transitions*, and (3) a dichotomous variable capturing three-month time windows around the signing of new constitutions.

Reassuringly, none of these additional control variables substantively change my results.

Appendix 3.4.1: Controlling for additional structural characteristics

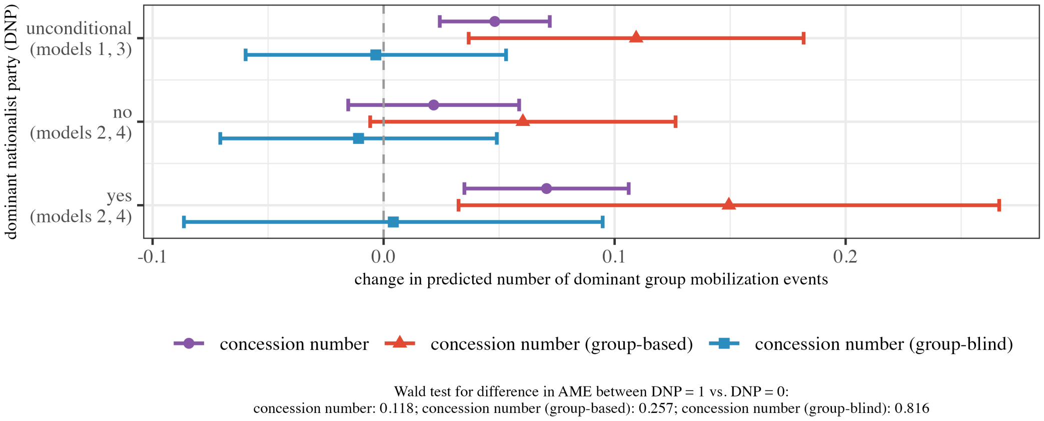

Figure A23. Partial effects and 90% confidence interval of concession number, concession number (group-based), and concession number (group-blind) on the number of dominant group mobilization events for observed values in my sample [additional controls: democratizing regime transition, autocratizing regime transition, unemployment rate, and GINI index].

Appendix 3.4.2: Accounting for the wider bargaining environment

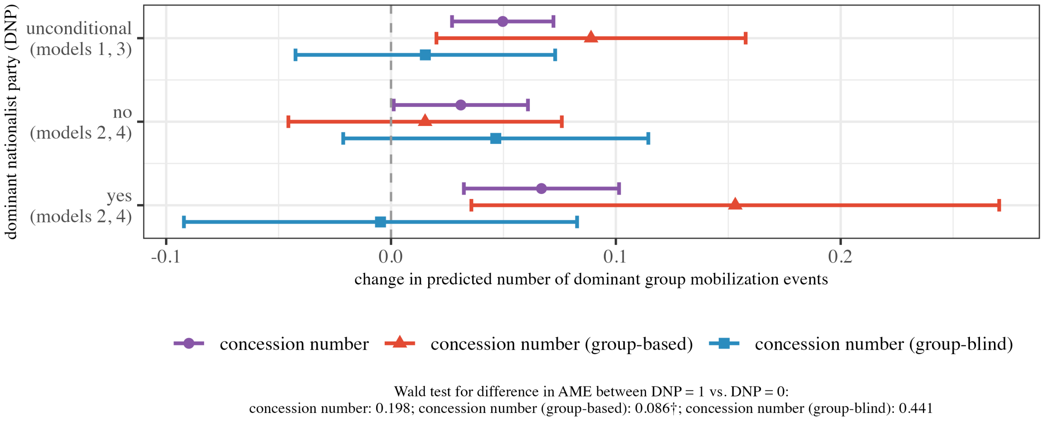

Figure A24. Partial effects and 90% confidence interval of concession number, concession number (group-based), and concession number (group-blind) on the number of dominant group mobilization events for observed values in my sample [additional controls: irredentist claims, self-determination movements, size difference majority-minority, years of political dominance].

Appendix 3.4.3: Controlling for additional interactions with number of dominant nationalist parties

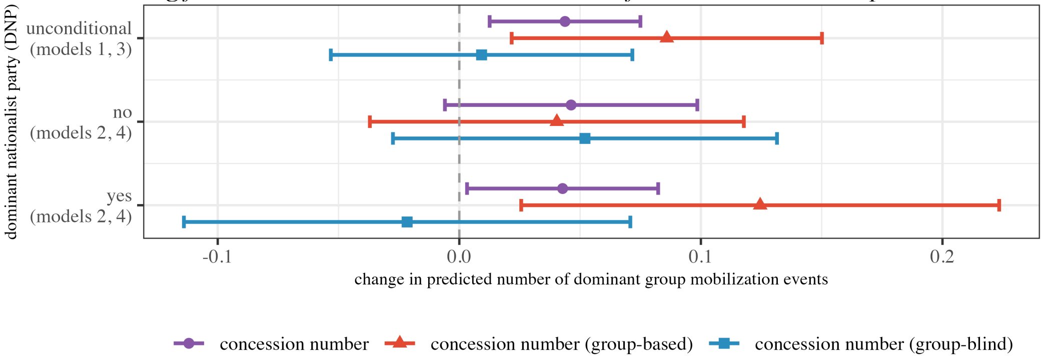

Wald test for difference in AME between DNP = 1 vs. DNP = 0:
concession number: 0.93; concession number (group-based): 0.275; concession number (group-blind): 0.316

Figure A25. Partial effects and 90% confidence interval of concession number, concession number (group-based), and concession number (group-blind) on the number of dominant group mobilization events for observed values in my sample [additional controls: interaction between *dominant nationalist party* and (logged) months to next election, transition periods, and new constitutional systems].

Appendix 3.5: Alternative specifications

In a fifth and final step, I show that my results remain similar when replacing my country month-level, negative binomial models with three alternative specifications: first, a linear count model (appendix 3.5.1) second, a logistic regression that takes the incidence of at least one dominant group mobilization event as the dependent variable (appendix 3.5.2); and third an alternative specification at the country year-level.[4] Again, my findings remain comparable to those attained in my main models, indicating that they are not sensitive to the specification I employ.

Appendix 3.5.1: Linear count model (logged number of dominant group mobilization events)

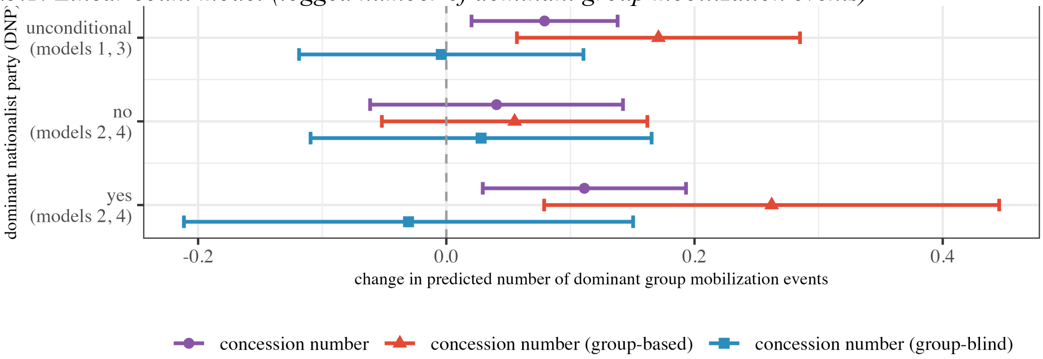

Wald test for difference in AME between DNP = 1 vs. DNP = 0:
concession number: 0.403; concession number (group-based): 0.108; concession number (group-blind): 0.679

Figure A26. Partial effects and 90% confidence interval of concession number, concession number (group-based), and concession number (group-blind) on the number of dominant group mobilization events for observed values in my sample [linear specification].

[4] For this purpose, I summed up all concessions and mobilization events in the same calendar year.

Appendix 3.5.2: Logistic model (incidence of at least one dominant group mobilization event)

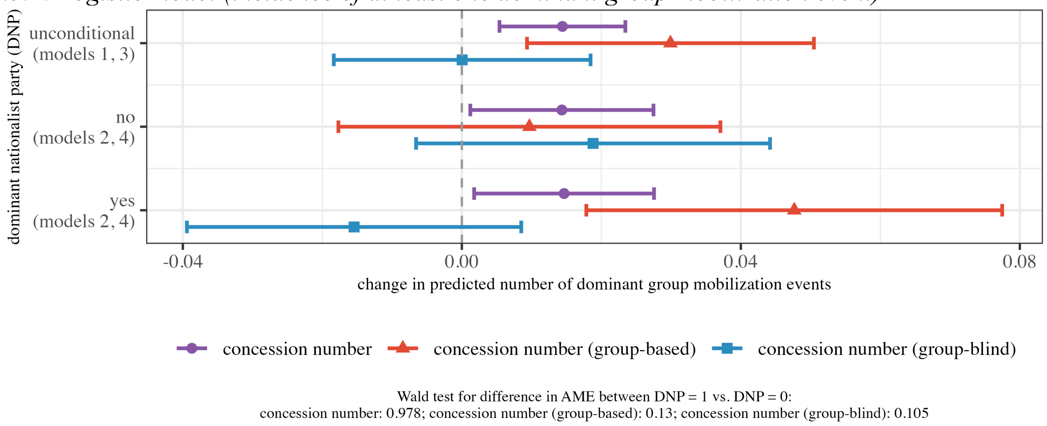

Figure A27. Partial effects and 90% confidence interval of concession number, concession number (group-based), and concession number (group-blind) on the incidence of dominant group mobilization events for observed values in my sample [logistic specification].

Appendix 3.5.3: Country year-level specification

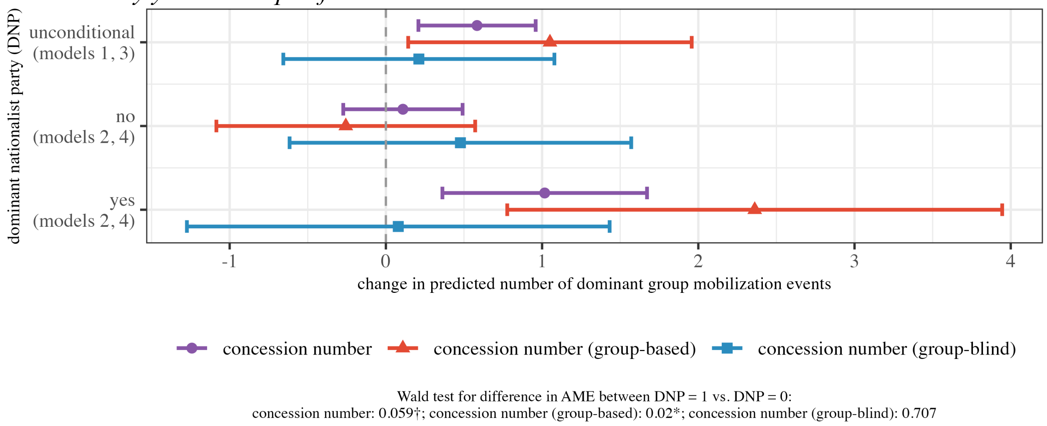

Figure A28. Partial effects and 90% confidence interval of concession number, concession number (group-based), and concession number (group-blind) on the incidence of dominant group mobilization events for observed values in my sample [country year-level specification].

Appendix 4: Individual-level analyses

In a final step, I provide more detail on the individual-level, survey-based analyses that focus on ordinary majority members' willingness to participate in protests. As argued in the main article text, if my postulated mechanisms apply, concessions that provide for ethnic accommodation should not only be associated with a higher frequency of actual dominant group mobilization, but also with a higher willingness to engage in non-institutional mobilization among ordinary members of the majority community. Moreover, this relationship should be particularly pronounced for conservative and authoritarian majority members, who I argued are particularly likely to form grievances and fears over the accommodation of ethnic subordinate groups. In what follows, I conduct a series of individual-level, survey-based analyses to probe these implications. I first provide more detail on the construction of individual-level dependent variables and controls (appendix 4.1). Second, I explain the multilevel specification used (appendix 4.2). And finally, I report the full model results (appendix 4.3), whose results I summarized visually using partial effects in the main article text (figure 4).

Appendix 4.1: Details on survey measures

For my individual-level analyses, I rely on the Integrated Values Surveys (IVS), itself a combination of World Values Surveys rounds 1-6 (Inglehart 2014) and European Values Surveys rounds 1-4 (EVS 2020). The Integrated Values Surveys contain several standardized survey items, which enable me to capture the individual-level implications of my theoretical argument reasonably well. Moreover, these items are available for a geographically diverse and temporally extensive sample, which is crucial for probing my argument, given the pronounced differences between different multi-ethnic countries' propensity to employ group-based or formally group-blind accommodation.

I start by identifying respondents belonging to the dominant group in the month each survey wave was administered. For this purpose, I combine monthly data on the identity of each sampled country's politically- dominant group, as derived in the main article text, with information on survey respondent ethnicity compiled by Juon (2023).

Using standardized survey items from multiple IVS rounds, I construct 7 dichotomous variables which I use in the individual-level analyses below: *willingness to attend protests* (my dependent variable),[5] *authoritarian*, *rightwinger* (both as individual-level control variables and as moderators), *age*, *female*, and *high education* (as individual-level control variables). Table A3 lists these variables and their underlying survey items used to code them.

Appendix 4.2: Model set-up

My unit of analysis is individual respondent *i* nested in country c and country-year *y*. Analogously to my main analyses, my sample encompasses all survey waves administered in countries with a V-Dem Polyarchy index score that is equal or higher than 0.4 (Coppedge et al. 2020) or in countries that are undergoing a democratic transition, according to the V-Dem Episodes of Regime Transition dataset (Maerz et al. 2021). I further follow Juon (2023) and only include country survey waves in which at least 50 dominant group members could be identified with reasonable probability (≥ 80%), to avoid a potentially biased sample composition, whereby my results could be disproportionately driven by a small number of imperfectly-identified survey respondents. Overall, my sample encompasses 176'635 respondents, clustered in 73 countries and 191 country years.[6]

I conduct my analysis with a set of logistic hierarchical multilevel models. These are suitable, as respondents are clustered into subgroups and therefore affected by common context. To account for this clustering, I include random intercepts at the levels of the country and country year. To account for different baselines of willingness to protest, I incorporate fixed effects at the level of the world region, as defined by the United Nations geoscheme.[7] Moreover, I include survey wave-fixed effects to account for survey item wordings that may differ slightly between various waves. In addition, I include all country- and group-level controls as in my main models, including year-fixed effects. I complement these by a series of individual-level controls for respondents' age, gender, high education levels, political interest, self-classification as politically rightwing, and authoritarian value orientation, as derived above (see table A3).

Table A4 reports the main results from six models. In models 1-3, I examine how individual willingness to protest is affected by past concessions, irrespective of their type (hypothesis 1). After introducing my term for past concessions in

[5] This dependent variable captures both instances where respondents indicated they participated in a demonstration in the past and instances where they might do so in the future. In an alternative specification (not reported), I exclude respondents that have already participated in demonstrations in the past. My results remain comparable.

[6] The included countries are Albania, Algeria, Argentina, Armenia, Australia, Austria, Azerbaijan, Bangladesh, Belarus, Belgium, Bosnia and Herzegovina, Brazil, Bulgaria, Canada, Chile, Colombia, Croatia, Cyprus, Ecuador, Egypt, El Salvador, Estonia, Ethiopia, Finland, France, Georgia, Ghana, Greece, Guatemala, Hungary, India, Indonesia, Iraq, Italy, Japan, Kazakhstan, Kosovo, Kyrgyzstan, Latvia, Lithuania, Macedonia, Malaysia, Mali, Mexico, Moldova, Montenegro, New Zealand, Nigeria, Pakistan, Peru, Philippines, Poland, Romania, Russia, Serbia, Slovakia, Slovenia, South Africa, Spain, Switzerland, Taiwan, Tanzania, Thailand, Trinidad and Tobago, Turkey, Uganda, Ukraine, United Kingdom, United States, Uruguay, Venezuela, Zambia, and Zimbabwe.

[7] In alternative specifications (not reported), I include country-fixed effects instead. However, as several countries are only included in one survey wave, this considerably reduces the variation available for my analyses and entails the risk of multicollinearity. Nevertheless, in these alternative specifications, my results remain comparable.

model 1, I proceed to interact this term with my individual-level moderating variables: *authoritarian* (model 2) and *rightwinger* (model 3). In models 4-6, I repeat this procedure, but introduce the distinction between past group-based and formally group-blind concessions (hypothesis 3). Figure 4 in the main article text visualizes the average marginal effects of these terms on the probability that an individual respondent states they are willing to protest.

Table A3. Survey items.

variable	question item	answer categories
willingness to attend protests	Now I'd like you to look at this card. I'm going to read out some different forms of political action that people can take, and I'd like you to tell me, for each one, whether you have actually done any of these things, whether you might do it or would never, under any circumstances, do it: attending lawful/peaceful demonstrations.	have done / might do / would never do
authoritarian*	(A) I'm going to describe various types of political systems and ask what you think about each as a way of governing this country. For each one, would you say it is a very good, fairly good, fairly bad or very bad way of governing this country? Having a strong leader	very good / fairly good / bad / very bad
	(B1) I will read you some goals which different people consider more or less important for this country. Could you please tell me how important you consider each one of these goals to be: would you say it is very important, important, not very important or not at all important for this country: maintaining order in the nation.	very important / important / not very important / not at all important
	(B2) If you had to choose, which one of the things on this card would you say is most important? And which would be the next most important?	maintaining order in the nation / giving people more say in important government decisions / fighting rising prices / protecting freedom of speech
	(B3) If you had to choose, which would you say is the most important responsibility of government?	maintain order in society / respect freedom of the individual / other answer
rightwinger	In political matters, people talk of "the left" and "the right." How would you place your views on this scale, generally speaking?	left / 2 / 3 / 4 / 5 / 6 / 7 / 8 / 9 / right
age	Can you tell me your year of birth, please?	NA
female	Sex	male / female
high education	highest educational level attained respondent	no formal education / incomplete/complete primary school / incomplete/complete secondary school: technical/vocational type / incomplete/complete secondary: university-preparatory type / some university without degree / university with degree
political interest	How interested would you say you are in politics?	very interested / somewhat interested / not very interested / not at all interested

Note: underlined answer category coded as 1, other answer categories coded as 0.
* *authoritarian* coded as 1 if respondents think having a strong leader is very good or fairly good (component A) and if they view maintaining order in the nation/society as (very) important or as one of the two main goals (components B1-B3, available for different survey waves).

Appendix 4.3: Full model results

Table A4. Individual-level results.

	Model 1	Model 2	Model 3	Model 4	Model 5	Model 6
Concession	-0.068	-0.114	-0.145			
	(0.111)	(0.111)	(0.112)			
Concession x authoritarian		0.210^{***}				
		(0.033)				
Concession x rightwinger			0.298^{***}			
			(0.027)			
Group-based concession				0.342^{**}	0.302^{*}	0.263^{*}
				(0.125)	(0.124)	(0.125)
Group-based concession x authoritarian					0.190^{***}	
					(0.032)	
Group-based concession x rightwinger						0.270^{***}
						(0.028)
Group-blind concession				-0.282^{*}	-0.317^{*}	-0.288^{*}
				(0.142)	(0.142)	(0.143)
Group-blind concession x authoritarian					0.077^{*}	
					(0.033)	
Group-blind concession x rightwinger						$0.050^{\dagger}$
						(0.028)
DN party	0.049	0.049	0.045	0.066	0.064	0.062
	(0.080)	(0.080)	(0.080)	(0.079)	(0.078)	(0.079)
DN party in government	-0.110	-0.108	-0.109	-0.139	-0.144	-0.135
	(0.149)	(0.149)	(0.149)	(0.145)	(0.145)	(0.145)
Months to next election (log)	-0.093	-0.099	-0.090	-0.075	-0.082	-0.073
	(0.081)	(0.081)	(0.082)	(0.081)	(0.080)	(0.081)
Recent subordinate group protest	0.692	0.691	0.661	0.965^{*}	0.944^{*}	0.948^{*}
	(0.437)	(0.436)	(0.438)	(0.438)	(0.436)	(0.437)
Recent civil violence	$-1.089^{\dagger}$	$-1.088^{\dagger}$	$-1.072^{\dagger}$	$-1.191^{\dagger}$	-1.200^{*}	$-1.184^{\dagger}$
	(0.621)	(0.620)	(0.622)	(0.608)	(0.605)	(0.608)
Battle deaths (last 10y, log)	-1.171^{***}	-1.166^{***}	-1.167^{***}	-1.060^{***}	-1.054^{***}	-1.056^{***}
	(0.233)	(0.233)	(0.234)	(0.230)	(0.229)	(0.230)
Democracy level	-1.781^{*}	-1.738^{*}	-1.827^{*}	-1.682^{*}	-1.604^{*}	-1.746^{*}
	(0.798)	(0.797)	(0.800)	(0.778)	(0.775)	(0.778)
Abs. size (log)	0.093^{**}	0.094^{**}	0.094^{**}	0.093^{**}	0.094^{**}	0.095^{**}
	(0.030)	(0.030)	(0.030)	(0.030)	(0.030)	(0.030)
GDP p.c. (log)	-0.227^{**}	-0.228^{**}	-0.230^{**}	-0.220^{**}	-0.221^{**}	-0.221^{**}
	(0.083)	(0.083)	(0.084)	(0.083)	(0.082)	(0.083)
GDP growth	0.021	0.021	0.017	-0.020	-0.027	-0.020
	(0.135)	(0.135)	(0.136)	(0.134)	(0.133)	(0.134)
Age	-0.018^{***}	-0.018^{***}	-0.018^{***}	-0.018^{***}	-0.018^{***}	-0.018^{***}
	(0.0003)	(0.0003)	(0.0003)	(0.0003)	(0.0003)	(0.0003)
Female	-0.302^{***}	-0.303^{***}	-0.304^{***}	-0.302^{***}	-0.302^{***}	-0.303^{***}
	(0.011)	(0.011)	(0.011)	(0.011)	(0.011)	(0.011)
High education	0.570^{***}	0.569^{***}	0.568^{***}	0.570^{***}	0.569^{***}	0.570^{***}
	(0.015)	(0.015)	(0.015)	(0.015)	(0.015)	(0.015)
Political interest	0.868^{***}	0.868^{***}	0.866^{***}	0.868^{***}	0.868^{***}	0.867^{***}
	(0.011)	(0.011)	(0.011)	(0.011)	(0.011)	(0.011)
Authoritarian	-0.067^{***}	-0.067^{***}	-0.281^{***}	-0.067^{***}	-0.066^{***}	-0.245^{***}
	(0.012)	(0.012)	(0.023)	(0.012)	(0.012)	(0.021)
Rightwinger	-0.184^{***}	-0.343^{***}	-0.184^{***}	-0.183^{***}	-0.352^{***}	-0.183^{***}
	(0.014)	(0.029)	(0.014)	(0.014)	(0.026)	(0.014)
Constant	13.667^{***}	13.616^{***}	13.637^{***}	12.964^{***}	12.972^{***}	12.937^{***}
	(3.650)	(3.644)	(3.659)	(3.581)	(3.565)	(3.579)
Country-FE	yes	yes	yes	yes	yes	yes
Year-FE	yes	yes	yes	yes	yes	yes
Survey wave-FE	yes	yes	yes	yes	yes	yes
N	175635	175635	175635	175635	175635	175635
Log Likelihood	-105058.300	-105037.600	-104997.300	-105054.300	-105021.000	-104980.400
AIC	210348.700	210309.300	210228.500	210342.600	210280.100	210198.800
BIC	211517.500	211488.200	211407.400	211521.600	211479.200	211397.800

$^{***}p < .01$; $^{**}p < .05$; $^{*}p < .1$

Additional references

Bormann, Nils-Christian, and Burcu Savun. 2018. "Reputation, Concessions, and Territorial Civil War: Do Ethnic Dominoes Fall, or Don't They?" *Journal of Peace Research* 55 (5): 671–86. https://doi.org/10.1177/0022343318767499.

Cammett, M., and E. Malesky. 2012. "Power Sharing in Postconflict Societies: Implications for Peace and Governance." *Journal of Conflict Resolution* 56 (6): 982–1016. https://doi.org/10.1177/0022002711421593.

Cederman, Lars-Erik, Kristian Skrede Gleditsch, and Julian Wucherpfennig. 2018. "The Diffusion of Inclusion: An Open-Polity Model of Ethnic Power Sharing." *Comparative Political Studies* 51 (10): 1279–1313. https://doi.org/10.1177/0010414017740602.

Cederman, Lars-Erik, Seraina Rüegger, and Guy Schvitz. 2022. "Redemption through Rebellion: Border Change, Lost Unity, and Nationalist Conflict." *American Journal of Political Science* 66 (1): 24–42. https://doi.org/10.1111/ajps.12634.

Cinelli, Carlos, and Chad Hazlett. 2020. "Making Sense of Sensitivity: Extending Omitted Variable Bias." *Journal of the Royal Statistical Society: Series B (Statistical Methodology)* 82 (1): 39–67. https://doi.org/10.1111/rssb.12348.

DeRouen, Karl, Jacob Bercovitch, and Paulina Pospieszna. 2011. "Introducing the Civil Wars Mediation (CWM) Dataset." *Journal of Peace Research* 48 (5): 663–72.

EVS. 2020. "European Values Study Longitudinal Data File 1981-2008 (EVS 1981-2008). ZA4804 Datenfile Version 3.1.0." GESIS Datenarchiv, Köln. https://doi.org/10.4232/1.13486.

Hillesund, Solveig (2019) Choosing Whom to Target: Horizontal Inequality and the Risk of Civil and Communal Violence. *Journal of Conflict Resolution* 63(2): 528–554.

Hyde, Susan D & Nikolay Marinov (2012) Which Elections Can Be Lost? *Political Analysis* 20(2): 191–201.

Juon, Andreas. 2020. "Subordinate groups Overlooked: Group-Based Power-Sharing and the Exclusion-amid-Inclusion Dilemma." *International Political Science Review* 41 (1): 89–107. https://doi.org/10.1177/0192512119859206.

Kreutz, Joakim (2010) How and when armed conflicts end: Introducing the UCDP Conflict Termination dataset. *Journal of Peace Research* 47(2): 243–250.

Lindberg, Staffan I., Nils Düpont, Masaaki Higashijima, Yaman Berker Kavasoglu, Kyle L. Marquardt, Michael Bernhard, Holger Döring, et al. 2022. *Varieties of Party Identity and Organization (V-Party) Dataset V2.* Varieties of Democracy (V-Dem) Project. https://doi.org/10.23696/vpartydsv2

Liu, Licheng, Ye Wang, and Yiqing Xu. 2024. "A Practical Guide to Counterfactual Estimators for Causal Inference with Time-Series Cross-Sectional Data." *American Journal of Political Science* 68 (1): 160–76. https://doi.org/10.1111/ajps.12723.

Mehler, Andreas. 2013. "Consociationalism for Weaklings, Autocracy for Muscle Men? Determinants of Constitutional Reform in Divided Societies." *Civil Wars* 15 (sup1): 21–43. https://doi.org/10.1080/13698249.2013.850874.

Melander, Erik, Frida Möller, and Magnus Öberg. 2009. "Managing Intrastate Low-Intensity Armed Conflict 1993–2004: A New Dataset." *International Interactions* 35 (1): 58–85. https://doi.org/10.1080/03050620902743887.

Pearson, Frederic S., and Robert A. Baumann. 1993. *International Military Intervention, 1946-1988*. Ann Arbor: University of Michigan.

Pickering, Jeffrey, and Emizet F. Kisangani. 2009. "The International Military Intervention Dataset: An Updated Resource for Conflict Scholars." *Journal of Peace Research* 46 (4): 589–99. https://doi.org/10.1177/0022343309334634.

Marshall, Monty G & Donna Ramsey Marshall (2022) *Coup d'État Events, 1946-2021. Codebook.* Center for Systemic Peace.

Roth, Jonathan, Pedro H.C. Sant'Anna, Alyssa Bilinski, and John Poe. 2023. "What's Trending in Difference-in-Differences? A Synthesis of the Recent Econometrics Literature." *Journal of Econometrics* 235 (2): 2218–44. https://doi.org/10.1016/j.jeconom.2023.03.008.

Rothchild, Joseph. 1981. *Ethnopolitics*. New York: Columbia University Press.

Sambanis, Nicholas, Micha Germann, and Andreas Schädel. 2018. "SDM: A New Data Set on Self-Determination Movements with an Application to the Reputational Theory of Conflict." *Journal of Conflict Resolution* 62 (3): 656–86. https://doi.org/10.1177/0022002717735364.

Samii, Cyrus. 2013. "Perils or Promise of Ethnic Integration? Evidence from a Hard Case in Burundi." *American Political Science Review* 107 (03): 558–73. https://doi.org/10.1017/S0003055413000282.

Tepšic, Goran, and Nemanja Džuverovic. 2018. "Bosnia and Herzegovina." In *The Elgar Companion to Post-Conflict Transition*, edited by Hans-Joachim Giessmann, Roger Mac Ginty, Beatrix Austin, and Christine Seifert, 27–48.

World Bank. 2020. "World Bank Development Indicators." World Development Indicators. 2020. https://data.worldbank.org/data-catalog/world-development-indicators.
