# Supplementary material for: Ethnic Accommodation and the Backlash From Dominant Groups
Source: J Conflict Resolut. 2025 May 22;70(2-3):359–86. doi: 10.1177/00220027251343836 (PMC12782309; doi:10.1177/00220027251343836)
Supplement: Supplemental Material - Ethnic Accommodation and the Backlash From Dominant Groups [file sj-pdf-2-jcr-10.1177_00220027251343836.pdf]

Ethnic accommodation and the backlash from dominant groups: Data supplement

Contents

S1: Concessions that provide for ethnic accommodation ... 2
S1.1: Coding rules: Horizontal concessions ... 2
S1.2: Coding rules: Vertical group-based concessions ... 2
S1.3: Coding rules: Vertical group-blind concessions ... 4
S1.4: Complete list of concessions ... 6
S2: Dominant group anti-government protest and violence directed against subordinate groups ... 19
S2.1 Ethnic attribution: general principles and sources ... 19
S2.2: Identification of ethnic actors ... 19
S2.3: Validation (non-state violence) ... 20
S3: Dominant nationalist parties ... 21
S3.1: Identification of dominant nationalist parties ... 21
S3.2: Complete list of dominant nationalist parties ... 22

S1: Concessions that provide for ethnic accommodation

S1.1: Coding rules: Horizontal concessions

To identify horizontal concessions, I combine existing data on de-facto power-sharing from the Ethnic Power Relations Dataset (EPR, Vogt et al. 2015) and constitutionally-enshrined power-sharing from the Constitutional Power-Sharing Dataset (CPSD, Juon 2023). I use these sources to identify horizontal concessions as follows:

- I identify ***group-based* horizontal concessions** by relying on the group-wise corporate power-sharing indices provided by CPSD. For each EPR group, these indices cover ethnically-based, constitutionally-entrenched provisions that formally mandate *grand coalitions or executive power-sharing* (most notably in the form of executive quotas for specific ethnic groups or ethnically-designated regions), *proportional representation* (most notably in the form of quotas for the lower or upper house provided to specific ethnic groups), and *mutual veto* rights (most notably in the form of rights provided to the representatives of specific ethnic groups to block certain types of constitutional amendments or legislation). I identify a group-based horizontal concession in any month where at least one subordinate group's corporate power-sharing index, which ranges from 0 to 1, increases by a value of at least 0.1. A prototypical example is the 2004 constitution in Burundi, which created two Vice Presidential positions, which had to be filled by members of two different ethnic groups (arts. 122-124). Simultaneously, it mandated that 40% of cabinet and National Assembly seats are reserved for the Tutsi minority (arts. 104, 108, 118, 129, 147, 164-169, 203).
- I identify ***group-blind* horizontal concessions** in any of the two following instances:
    - First, I capture constitutional amendments that introduce **non-ethnic, electorally-based power-sharing**, using the group-wise liberal power-sharing indices from CPSD. For each EPR group, these indices cover electorally-based constitutional provisions that mandate *grand coalitions or executive power-sharing* (most notably in the form of provisions that enable each party above a specific vote share to attain ministerial seats or that mandate supermajority requirements for the election of important executive positions), *proportional representation* (most notably in the form of proportional electoral systems), and *mutual veto* rights (most importantly, supermajority requirements for the promulgation of constitutional amendments or general legislation). I identify a group-blind horizontal concession in any month where at least one subordinate group's liberal power-sharing index, which ranges from 0 to 1, increases by a value of at least 0.1. An example is South Africa's 1993 constitution, which came into force in April 1994. This allowed each party holding at least 80 seats in the National Assembly to attain an Executive Deputy-President position (arts. 84, 94), gave each party with at least 20 seats in the Assembly the right to be proportionally represented in the cabinet (arts. 88-93), introduced a highly proportional electoral system with a district magnitude of 200 and no legal threshold (arts 36-40, 50, 73, 93, schedule 2), and required a two-thirds supermajority for the promulgation of constitutional amendments (arts. 62-64).
    - Second, I capture **informal, de-facto power-sharing**, using information from EPR on the de-facto representation of subordinate groups in the executive. I identify a group-blind horizontal concession in each month in which at least one subordinate group becomes newly included in the executive whose timing was not preceded by corresponding increases of said group's corporate/liberal power-sharing indices (see above) in the preceding 12 months. An example is the (renewed) inclusion of ethnic Hungarian representatives into Slovakia's government in 1999, 2010, and 2016, which was due to informal electoral coalitions that were not constitutionally mandated.

For more details, I refer to the respective articles that describe the underlying data sources in more detail (Vogt et al. 2015; Juon 2020).

S1.2: Coding rules: Vertical group-based concessions

As stated in the main article text, I operationalize vertical group-based concessions as constitutional changes that expand the recognition of subordinate groups' identities, their cultural rights, and customs. To identify such concessions, I screened all constitutions that were operational in the studied time period in my sampled countries. I checked all constitutional events coded by the Comparative Constitutions Project (Elkins, Ginsburg & Melton 2014). I obtained original texts for each of these constitutional events, predominantly relying on Hein Online's World Constitutions Illustrated database[1] and the Constitute Database.[2] Using these sources, I identify the following aspects of group-based vertical accommodation for each constitutional text.

- **Recognition of subordinate groups' parties:** The constitution recognizes political parties that represent specific subordinate groups. For example, Sudan's 2005 constitution referred to the Sudan People's Liberation

[1] Available at <https://home.heinonline.org/content/world-constitutions-illustrated/>.
[2] Available at <https://www.constituteproject.org/>.

Movement as the representative of South Sudan (art. 68), a party that represented the Beja, Dinka, Nuer, Shilluk, and Other Southern Groups according to ACD2EPR (Vogt et al. 2015).

- **Recognition of subordinate groups' ethnic identities:** The constitution explicitly recognizes specific subordinate groups as constituent parts of the nation. For example, Ecuador's 1998 constitution explicitly recognized indigenous communities and Afro-Ecuadorians as national subordinate groups (art. 83).
- **Recognition subordinate groups' religions**: The constitution explicitly recognizes the religions practiced by subordinate groups. For example, North Macedonia's 2001 constitution recognized not only the Macedonian Orthodox Church, but also the Islamic Religious Community, the Catholic Church, the Evangelical Methodist Church, and the Jewish Community (arts. 19, 48, amendment VII, amendment VIII).
- **Recognition of subordinate groups' languages**: The constitution explicitly recognizes the languages spoken by subordinate groups. For example, South Africa's 1993 constitution recognized Afrikaans, English, isiNdebele, Sesotho sa Leboa, Sesotho, siSwati, Xitsonga, Setswana, Tshivenda, isiXhosa, and isiZulu as official languages (arts. 3, 31).
- **Recognition of ethnic organizations**: The constitution recognizes specific subordinate groups' (non-partisan) organizations or their right to establish such organizations. For example, Bolivia's 1994 constitution recognized the "legal personality" of indigenous and peasant communities and their associations (art. 171).
- **Recognition of religious organizations:** The constitution recognizes the organizations belonging to religions practiced by subordinate groups or their right to establish such organizations. For example, North Macedonia's 2001 constitution explicitly states that the Islamic Religious Community, the Catholic Church, the Evangelical Methodist Church, the Jewish Community, and other religious communities are free to establish their own social and charitable institutions (art. 19, amendment VII).
- **Recognition of linguistic organizations:** The constitution recognizes organizations that promote or are based on languages spoken by subordinate groups or establishes their to establish such organizations. For example, Albania's 1998 constitution stipulated that national minorities have the right to unite in organizations and associations for the protection of their interests, language, and identity (art. 20).
- **Recognition of cultural customs:** The constitution recognizes subordinate groups' cultural customs or their right to uphold such customs. For example, Mexico's 2001 constitution recognized the customs of indigenous tribes, along with the right to "enrich" their culture and identity (art. 2).
- **Recognition of religious practices:** The constitution guarantees the right to practice religions that subordinate groups' members adhere to. For example, North Macedonia's 2001 constitution provides the right to express one's faith publicly (art. 19) and simultaneously recognizes not only the Macedonian Orthodox Church, but also the Islamic Religious Community, the Catholic Church, the Evangelical Methodist Church, the Jewish Community, and other religious.
- **Recognition of land rights:** The constitution recognizes subordinate groups' rights to their ancestral land. For example, Sudan's 2015 constitution recognized the "historical land rights" in Darfur (art. 226; Doha Document for Peace in Darfur, art. 123).
- **Recognition of culturally-distinct education systems:** The constitution recognizes subordinate groups' rights to education curricula that take into account or follow their cultural customs. For instance, Ecuador's 1998 constitution stipulated the right of indigenous peoples and Afro-Ecuadorians to intercultural education (arts. 63-69).
- **Recognition of right to separate religious schooling:** The constitution recognizes the right to education in religions practiced by subordinate groups or to education curricula that follow the religion of subordinate groups. For example, Malaysia's 1957 constitution awards every religious group the right to establish institution for the education of children in their own religion (arts. 11, 12).
- **Recognition of right to native language education:** The constitution recognizes the right to education in languages spoken by subordinate groups. For example, Estonia's 1992 constitution stipulated that, in national minority educational institutions, the language is chosen by the specific educational institution (art. 37). Simultaneously, it explicitly recognized the German, Russian, Swedish, and Jewish national minorities, to whom these rights applied (preamble, art. 50, national minorities cultural autonomy act, art. 2).
- **Recognition of right to separate national media:** The constitution recognizes the rights of specific subordinate groups to establish their own media or stipulates state support for such media. For example, Mexico's 2001 constitution stipulated that media platforms will be established specifically for the indigenous peoples (art. 2).
- **Recognition of right to native language media:** The constitution recognizes the rights to establish media in languages spoken by subordinate groups or stipulates state support for such media. For example, Ecuador's 2008 constitution awarded indigenous minorities the right to receive media in their own languages (art. 57).
- **Recognition of right to culturally-distinct judiciary systems:** The constitution recognizes the rights of subordinate groups to their culturally-distinct or customary judiciary systems. For example, the Democratic Republic Congo's 2005 constitution explicitly recognized customary law (art. 204). Simultaneously, it

recognized various ethnic groups and nationalities making up the DRC, to whom these provisions applied (art. 10).

- **Recognition of subordinate groups' religious law:** The constitution recognizes legal sources or legal bodies connected to the religions practiced by subordinate groups. For example, Indonesia's 2001 constitution recognized religious affairs courts below the national Supreme Court (art. 24.2).
- **Recognition of right to use native language in court:** The constitution provides the right to use languages spoken by subordinate groups in court. For example, South Africa's 1993 constitution established the right to use a "South African language of choice" in front of the courts (art. 107). Simultaneously, it recognized Afrikaans, English, isiNdebele, Sesotho sa Leboa, Sesotho, siSwati, Xitsonga, Setswana, Tshivenda, isiXhosa, and isiZulu as official South African languages (arts. 30-31).
- **Recognition of subordinate groups' ethnic institutions:** The constitution recognizes institutions that are separate from the government which are dedicated to upholding the rights of or that are directly connected to subordinate groups. For example, Nepal's 2015 constitution established a National Dalit commission to carry out research on Dalit matters and a National Inclusion commission to do the same for Khash Arya (arts. 256, 259).
- **Recognition of subordinate groups' religious authorities:** The constitution recognizes institutions connected to the religions practiced by subordinate groups or stipulates state policy (nationally or in a given region) should follow the religious values of subordinate groups. For example, Sudan's 2005 constitution stipulated that national legislation for the Northern States has Sharia law as the basis, while national legislation for Southern Sudan has as source the customs and values of the local people and their religious beliefs. It also stipulated that, in the capital, there is a commission for the rights of Non-Muslims (arts. 5, 157).
- **Recognition of right to use native language in communication with government:** The constitution provides the right to use the languages spoken by subordinate groups in communication with or in consultations by the national or regional government. For example, Iraq's 2005 constitution mandated that national laws be published in both Arabic and Kurdish and that government institutions work in both languages. It also mandated that Turkmen and Syriac are official languages in the administrative units where they constitute a certain "density of population". Additionally, it allowed each region or governorate to adopt any other local language as an additional official language by referendum (art. 4).
- **Recognition of subordinate groups' national symbols:** The constitution recognizes or refers to the national or cultural symbols of subordinate groups. For example, Niger's 1992 constitution stipulated that the arms of the republic contain two Tuareg spades (art. 1).
- **Recognition of subordinate groups' religious symbols:** The constitution recognizes or refers to the symbols of a religion practiced by subordinate groups. For example, Benin's 1990 constitution stipulated that the presidential oath contain a reference to God and to the Manes (spirits) of the ancestors (art. 53).
- **Recognition of subordinate groups' linguistic symbols:** The constitution refers to the symbolic, public use of languages spoken by subordinate groups, for instance in terms of alphabets or street signs. For example, Montenegro's 2007 constitution awarded minority nationalities the right to use their own language and alphabet on official documents and the right to use their traditional local terms and names of streets and settlements (art. 79). It explicitly recognized as applicable national minorities Serbs, Bosniacs, Albanians, Muslims, Croats, and the "others" (preamble).

For each of the provisions so identified, I noted down its specific identity basis (in terms of ethnicity, religion, language). I then connected each provision to the ethnic groups in my sample, either directly by ethnic name or indirectly by religion practiced or language spoken by each group, according to the EPR-Ethnic Dimensions dataset (EPR-ED, Vogt et al. 2015). For each subordinate group I then summed up the number of applicable provisions in each constitutional period. I use this cumulative measure to identify a group-based vertical concession in any month during which this measure increases by at least one for at least one subordinate group in my sample.

In the supplementary material, I provide full group-level data for each of these indicators, covering all constitutional events across all countries in my sample between 1990 and 2018. Moreover, I also provide short text-based justifications and references to the relevant constitutional articles for each constitutional text I used to code these indicators.

S1.3: Coding rules: Vertical group-blind concessions

As stated in the main article text, I operationalize vertical group-blind concessions in months where institutions were introduced or expanded which provide for autonomous self-rule in regions inhabited by subordinate groups. I start by identifying "candidate" autonomy arrangements. For this purpose, I again assessed all constitutional texts that were operative at any time point for my sample (Elkins, Ginsburg & Melton 2014), using the original texts for each of these constitutional events, as obtained from Hein Online's World Constitutions Illustrated database[3] and the Constitute

[3] Available at <https://home.heinonline.org/content/world-constitutions-illustrated/>.

Database.[4] In these texts, I identified arrangements that the constitution calls "confederal", "federal", "decentralized", or "autonomous".

I then assessed, based on the obtained **constitutions and any autonomy statutes** referenced by it, each "candidate" unit's degree of autonomy. Similar to the Regional Authority Index (RAI) (Hooghe et al. 2016), I coded six region-level indicators, which are grouped along three components: policy autonomy (*institutional depth* and *policy scope*), fiscal autonomy (*taxing competencies* and *financial guarantees*), and political autonomy (*independent legislature* and *independent executive*). Each of these indicators is an ordinal measure that increases with each region's degree of autonomy:

- **Institutional depth** refers to the degree to which a region's decisions are independent from central government interference. This indicator is an ordinal variable with four levels {0,3}: 0 = no autonomous decision-making (e.g., no institutionalized autonomy or deconcentrated tier that implements central government decisions); 1 = decision-making subject to ex-ante central government approval; 2 = decision-making subject to ex-post central government veto; 3 = decision-making not subject to either central government approval or veto.
- **Policy scope** captures the number and relative importance of issue areas that each region may legislate on. This indicator is a weighted sum {0,20} of 10 competencies coded as binary sub-indicators: matters related to economy (weight = 1), welfare (1), culture (1), military (2), police (2), judiciary (2), institutional set-up (2), residual powers (3), community (3), and secession rights (3).
- **Taxing competencies** capture the region's influence over the rate and base of minor and major taxes. This indicator is an ordinal variable with five levels {0,4}: 0 = no taxing competencies; 1 = competency to set the rate of minor taxes; 2 = competency to set base and rate of minor taxes; 3 = competency to set the rate of major taxes; 4 = competency to set base and rate of major taxes. Major taxes include personal income, corporate, value added, and sales taxes.
- **Borrowing competencies** refer to the degree that a region can borrow money independently from central government authorization and restrictions. This indicator is an ordinal variable with four levels {0,3}: 0 = no borrowing competencies; 1 = borrowing possible under ex-ante authorization by the central government; 2 = borrowing possible without ex-ante authorization, but with restrictions; 3 = borrowing possible without authorization or restrictions.
- **Independent legislature** captures each region's ability to select its own legislative institutions independently from central government interference. This indicator is an ordinal variable with three levels {0,2}: 0 = subnational legislature does not exist or is fully appointed by the central government; 1 = subnational legislature is partly selected by the local population or local elites, partly appointed by the central government; 2 = subnational legislature is fully selected by the local population or local elites.
- **Independent executive** captures each region's ability to select its own executive institutions independently from central government interference. This indicator is an ordinal variable with three levels {0,2}: 0 = subnational executive does not exist or is fully appointed by the central government; 1 = subnational executive is partly selected by the local population or local elites, partly appointed by the central government; 2 = subnational executive is fully selected by the local population or local elites.

I sum up these indicators to obtain an overall autonomy score for each region in each constitutional period, ranging from 0 to 34. To identify each ethnic subordinate group's degree of autonomy, I spatially intersect the boundaries of each coded region, predominantly taken from the Database of Global Administrative Areas (GADM),[5] with each ethnic group's settlement patterns, as provided by GeoEPR (Vogt et al. 2015). Based on these intersections, I calculate the degree of autonomy in regions settled by the average member of each group, again ranging from 0 to 34. I identify group-blind vertical concessions in months where the degree of autonomy by at least one subordinate group increases by at least 0.1. In the vast majority of cases, this is due to changes in the underlying constitutional texts and autonomy statutes, for instance where the policy scope of regions inhabited by subordinate groups is expanded. In a small number of cases, this is due to changes in administrative boundaries which substantially increase the autonomy enjoyed by the average member of at least one subordinate group.

In the supplementary material, I provide both region- and group-level data for each of these indicators, covering all countries in my sample between 1990 and 2018. Moreover, I also provide short text-based justifications and references to the relevant constitutional articles for each constitutional text used to code these indicators.

[4] Available at <https://www.constituteproject.org/>.
[5] Available online under <http://gadm.org/data.html>.

S1.4: Complete list of concessions

Table S1. Complete list of concessions**.**

country	year	month	type of concession
Canada	1991	12	Group-blind (v): policy scope (community)
Mexico	1996	12	Group-blind (v): policy scope (welfare)
Mexico	2001	9	Group-based (v): recognition of subordinate groups' ethnic identity; language; cultural customs; land rights; culturally-distinct education; right to native language education; separate national media; culturally-distinct judiciary systems; native language use in courts; ethnic institutions; national symbols
El Salvador	2014	12	Group-based (v): recognition of subordinate groups' ethnic identity; cultural customs
Costa Rica	1999	12	Group-based (v): recognition of subordinate groups' language
Panama	1996	12	Group-blind (v): institutional depth
Panama	1997	4	Group-blind (v): institutional depth; policy scope (economy, culture, welfare, police, judiciary, institutions); regional legislature; regional executive
Panama	2000	12	Group-blind (v): change in administrative boundaries
Panama	2009	12	Group-blind (v): change in administrative boundaries
Colombia	1991	8	Group-based (h): proportional representation<br>Group-based (v): recognition of subordinate groups' ethnic identity; language; cultural customs; land rights; culturally-distinct education; right to native language education; religious authorities; native language use in government; national symbols; religious symbols<br>Group-blind (v): institutional depth; policy scope (economy, culture, welfare, police, institutions); taxing autonomy; borrowing autonomy; regional legislature; regional executive
Colombia	1999	12	Group-based (v): recognition of subordinate groups' ethnic identity
Venezuela	1991	12	Group-blind (v): change in administrative boundaries
Venezuela	1992	12	Group-blind (v): institutional depth; policy scope (police, institutions, residual powers); borrowing autonomy; regional executive
Venezuela	1994	12	Group-blind (v): policy scope (welfare)
Venezuela	1998	12	Group-blind (v): change in administrative boundaries
Venezuela	2000	1	Group-based (v): recognition of subordinate groups' religion; language; cultural customs; religious practices; land rights; culturally-distinct education; right to native language education; culturally-distinct judiciary systems; native language use in courts; religious authorities; native language use in government<br>Group-blind (v): policy scope (economy, culture); taxing autonomy
Guyana	2001	8	Group-based (h): grand coalition<br>Group-based (v): recognition of subordinate groups' ethnic identity; language; ethnic NGOs; cultural customs; land rights; ethnic institutions
Ecuador	1993	6	Group-based (v): recognition of subordinate groups' religious authorities
Ecuador	1996	8	Group-based (h): proportional representation
Ecuador	1998	7	Group-based (v): recognition of subordinate groups' ethnic identity; cultural customs; land rights; culturally-distinct education; culturally-distinct judiciary systems; ethnic institutions; native language use in government; national symbols<br>Group-blind (v): policy scope (culture); taxing autonomy
Ecuador	2002	12	Group-based (h): proportional representation<br>Group-blind (h): proportional representation
Ecuador	2008	10	Group-based (v): recognition of subordinate groups' religion; language; ethnic NGOs; separate national media; right to native language media; religious authorities<br>Group-blind (v): policy scope (judiciary); regional legislature
Peru	1994	1	Group-based (v): recognition of subordinate groups' culturally-distinct judiciary systems<br>Group-blind (v): institutional depth; policy scope (economy, institutions); taxing autonomy; regional legislature; regional executive
Brazil	2002	11	Group-blind (h): informal inclusion
Bolivia	1993	1	Group-blind (h): informal inclusion

Table S1. Complete list of concessions**.**

country	year	month	type of concession
Bolivia	1994	9	Group-based (v): recognition of subordinate groups' ethnic identity; language; ethnic NGOs; cultural customs; land rights; culturally-distinct judiciary systems; ethnic institutions<br>Group-blind (v): regional legislature
Bolivia	2006	1	Group-blind (h): informal inclusion
Bolivia	2009	3	Group-based (h): grand coalition; proportional representation<br>Group-based (v): recognition of subordinate groups' ethnic identity; religion; language; cultural customs; land rights; culturally-distinct education; right to native language education; ethnic institutions; religious authorities; native language use in government; national symbols<br>Group-blind (v): institutional depth; policy scope (economy, culture, welfare, police, judiciary, institutions); taxing autonomy; borrowing autonomy; regional executive
Paraguay	1992	7	Group-based (v): recognition of subordinate groups' ethnic identity; religion; language; cultural customs; land rights; culturally-distinct education; right to native language education; culturally-distinct judiciary systems; ethnic institutions<br>Group-blind (v): policy scope (economy); borrowing autonomy; regional legislature; regional executive
Chile	1991	12	Group-blind (v): regional legislature
Chile	2005	9	Group-blind (h): proportional representation<br>Group-blind (v): policy scope (economy, culture)
Chile	2009	12	Group-blind (v): regional executive
Argentina	1995	1	Group-based (h): proportional representation<br>Group-based (v): recognition of subordinate groups' ethnic identity; ethnic NGOs; cultural customs; land rights; culturally-distinct education
United Kingdom	1998	12	Group-blind (v): institutional depth; policy scope (economy, culture, welfare, police, judiciary, institutions, residual powers); taxing autonomy; borrowing autonomy; regional legislature; regional executive
United Kingdom	2011	8	Group-blind (v): policy scope (welfare, institutions); regional executive
Belgium	1993	8	Group-based (v): recognition of subordinate groups' ethnic institutions<br>Group-blind (v): institutional depth; policy scope (economy); taxing autonomy; borrowing autonomy; regional legislature; regional executive
Belgium	1995	1	Group-based (h): proportional representation
Belgium	2001	4	Group-blind (v): policy scope (institutions)
Belgium	2005	12	Group-blind (v): policy scope (institutions)
Belgium	2017	12	Group-blind (v): change in administrative boundaries
France	1992	7	Group-based (v): recognition of subordinate groups' language
Switzerland	1999	5	Group-based (h): grand coalition
Spain	1996	12	Group-blind (v): taxing autonomy
Poland	1992	11	Group-based (v): recognition of subordinate groups' religious symbols<br>Group-blind (h): proportional representation
Poland	1997	11	Group-based (v): recognition of subordinate groups' ethnic identity; language; ethnic NGOs; religious NGOs; cultural customs; culturally-distinct education; ethnic institutions; religious authorities<br>Group-blind (v): institutional depth; policy scope (residual powers); taxing autonomy; regional legislature; regional executive
Austria	2000	9	Group-based (v): recognition of subordinate groups' ethnic identity; language; cultural customs
Hungary	1993	7	Group-based (v): recognition of subordinate groups' cultural customs; culturally-distinct education; national symbols<br>Group-blind (v): policy scope (culture)
Hungary	2011	5	Group-based (v): recognition of subordinate groups' religion
Hungary	2013	12	Group-based (v): recognition of subordinate groups' religious NGOs
Czechoslovakia	1990	12	Group-blind (v): institutional depth; policy scope (military, police); taxing autonomy

Table S1. Complete list of concessions**.**

country	year	month	type of concession
Slovakia	1999	1	Group-blind (h): informal inclusion
Slovakia	2010	7	Group-blind (h): informal inclusion
Slovakia	2016	4	Group-blind (h): informal inclusion
Italy/Sardinia	1990	12	Group-blind (v): taxing autonomy
Italy/Sardinia	1997	12	Group-blind (v): policy scope (residual powers)
Italy/Sardinia	1998	12	Group-blind (v): taxing autonomy
Italy/Sardinia	2001	12	Group-blind (v): institutional depth; policy scope (culture)
Albania	1991	6	Group-blind (h): mutual veto
Albania	1992	5	Group-blind (h): proportional representation
Albania	1998	11	Group-based (v): recognition of subordinate groups' religion; language; linguistic NGOs; cultural customs; right to native language education; native language use in courts; native language use in government; religious symbols<br>Group-blind (v): institutional depth; taxing autonomy; regional legislature
Albania	2011	12	Group-based (v): recognition of subordinate groups' ethnic identity; cultural customs
Montenegro	2007	11	Group-based (v): recognition of subordinate groups' culturally-distinct judiciary systems; linguistic symbols
Montenegro	2016	11	Group-blind (h): informal inclusion
Macedonia (Former Yugoslav Republic Of)	1998	7	Group-blind (h): proportional representation
Macedonia (Former Yugoslav Republic Of)	2001	12	Group-based (h): mutual veto<br>Group-based (v): recognition of subordinate groups' ethnic identity; religion; language; ethnic NGOs; religious NGOs; cultural customs; religious practices; culturally-distinct education; right to native language education; native language use in government; national symbols
Macedonia (Former Yugoslav Republic Of)	2004	9	Group-blind (v): institutional depth; policy scope (economy, culture, welfare); taxing autonomy; regional legislature; regional executive
Macedonia (Former Yugoslav Republic Of)	2005	12	Group-blind (v): borrowing autonomy
Croatia	1993	1	Group-blind (v): change in administrative boundaries
Croatia	1995	10	Group-based (h): proportional representation
Serbia / Serbia and Montenegro	1992	5	Group-based (v): recognition of subordinate groups' religion; ethnic NGOs; religious practices; culturally-distinct education; separate national media; right to native language media; ethnic institutions; national symbols<br>Group-blind (h): proportional representation; mutual veto<br>Group-blind (v): institutional depth; borrowing autonomy
Serbia / Serbia and Montenegro	2003	3	Group-blind (v): institutional depth; policy scope (economy, culture, welfare, police, judiciary, institutions, community, secession); taxing autonomy; borrowing autonomy; regional legislature; regional executive
Serbia / Serbia and Montenegro	2005	12	Group-blind (h): proportional representation
Serbia / Serbia and Montenegro	2009	8	Group-blind (v): policy scope (culture)
Bosnia and Herzegovina	1999	12	Group-blind (v): change in administrative boundaries
Bosnia and Herzegovina	2008	1	Group-based (v): recognition of subordinate groups' religion; religious practices; religious law
Greece	2011	1	Group-blind (v): institutional depth; policy scope (economy, culture, welfare); borrowing autonomy; regional legislature; regional executive
Bulgaria	1991	1	Group-based (v): recognition of subordinate groups' language; linguistic symbols<br>Group-blind (h): grand coalition; mutual veto

Table S1. Complete list of concessions**.**

country	year	month	type of concession
Bulgaria	1991	8	Group-based (v): recognition of subordinate groups' religion<br>Group-blind (h): proportional representation; mutual veto
Bulgaria	2001	7	Group-blind (h): informal inclusion
Bulgaria	2013	6	Group-blind (h): informal inclusion
Moldova	1994	8	Group-based (v): recognition of subordinate groups' language; right to native language education; native language use in courts<br>Group-blind (h): mutual veto
Moldova	1995	8	Group-based (v): recognition of subordinate groups' ethnic identity<br>Group-blind (v): institutional depth; policy scope (economy, culture, welfare, institutions); taxing autonomy; regional legislature; regional executive
Moldova	1999	1	Group-blind (h): proportional representation<br>Group-blind (v): change in administrative boundaries
Moldova	2001	3	Group-blind (h): informal inclusion
Moldova	2005	12	Group-blind (v): institutional depth; policy scope (economy, culture, welfare); regional legislature; regional executive
Romania	1991	12	Group-based (h): proportional representation<br>Group-based (v): recognition of subordinate groups' religion; cultural customs; religious symbols<br>Group-blind (h): proportional representation; mutual veto<br>Group-blind (v): institutional depth; policy scope (economy); taxing autonomy; regional legislature; regional executive
Romania	1996	1	Group-blind (h): informal inclusion
Romania	2003	11	Group-based (h): proportional representation<br>Group-based (v): recognition of subordinate groups' native language use in government
Romania	2009	12	Group-blind (h): informal inclusion
Romania	2014	12	Group-blind (h): informal inclusion
Russia	1992	1	Group-based (h): grand coalition; proportional representation<br>Group-based (v): recognition of subordinate groups' ethnic identity; cultural customs; land rights; ethnic institutions<br>Group-blind (h): grand coalition; proportional representation; mutual veto<br>Group-blind (v): institutional depth; policy scope (economy, culture, welfare, police, judiciary, institutions, residual powers); taxing autonomy; regional legislature; regional executive
Russia	1992	5	Group-based (h): grand coalition; proportional representation<br>Group-blind (v): institutional depth; policy scope (economy, culture, welfare, police, judiciary, institutions, residual powers); taxing autonomy; regional legislature; regional executive
Russia	1994	1	Group-based (h): proportional representation; mutual veto<br>Group-based (v): recognition of subordinate groups' ethnic identity; language; native language use in government<br>Group-blind (h): proportional representation; mutual veto<br>Group-blind (v): institutional depth; policy scope (police, judiciary, institutions, residual powers); taxing autonomy; borrowing autonomy; regional executive
Russia	1994	12	Group-blind (v): policy scope (community)
Russia	1995	12	Group-blind (h): proportional representation<br>Group-blind (v): institutional depth
Russia	1997	12	Group-blind (v): institutional depth; borrowing autonomy
Russia	1998	1	Group-blind (v): institutional depth; borrowing autonomy
Russia	1999	12	Group-blind (v): change in administrative boundaries
Russia	2000	12	Group-blind (v): change in administrative boundaries
Russia	2007	12	Group-blind (v): institutional depth; policy scope (economy, culture, welfare, police, judiciary, institutions, residual powers); taxing autonomy; borrowing autonomy; regional legislature; regional executive

Table S1. Complete list of concessions**.**

country	year	month	type of concession
Russia	2008	12	Group-blind (v): institutional depth; taxing autonomy; borrowing autonomy; regional legislature
Russia	2013	1	Group-based (h): proportional representation<br>Group-blind (v): change in administrative boundaries
Russia	2014	4	Group-blind (h): proportional representation; mutual veto
Russia	2017	1	Group-blind (v): institutional depth; policy scope (economy, culture, welfare, police, judiciary, institutions, residual powers); taxing autonomy; borrowing autonomy; regional legislature; regional executive
Estonia	1992	7	Group-based (v): recognition of subordinate groups' language; right to native language education; native language use in courts; native language use in government<br>Group-blind (h): proportional representation; mutual veto
Estonia	1993	11	Group-based (v): recognition of subordinate groups' cultural customs; culturally-distinct education; national symbols<br>Group-blind (v): policy scope (culture)
Latvia	1998	11	Group-based (v): recognition of subordinate groups' language; native language use in government; linguistic symbols
Latvia	2014	8	Group-based (v): recognition of subordinate groups' religion; religious NGOs; religious practices
Lithuania	1992	11	Group-based (v): recognition of subordinate groups' ethnic identity; religion; religious NGOs; religious practices
Ukraine	1996	7	Group-based (v): recognition of subordinate groups' religion; language; ethnic NGOs; cultural customs; culturally-distinct education; right to native language education
Ukraine	1997	1	Group-based (v): recognition of subordinate groups' ethnic identity; ethnic NGOs; cultural customs; culturally-distinct education<br>Group-blind (h): proportional representation
Ukraine	1998	1	Group-based (v): recognition of subordinate groups' ethnic identity; ethnic NGOs; cultural customs; culturally-distinct education<br>Group-blind (v): taxing autonomy
Ukraine	2014	3	Group-based (v): recognition of subordinate groups' ethnic NGOs; cultural customs; culturally-distinct education<br>Group-blind (v): institutional depth; policy scope (economy, culture, welfare, institutions); taxing autonomy; regional legislature; regional executive
Belarus	1994	4	Group-blind (h): mutual veto
Belarus	1996	12	Group-based (v): recognition of subordinate groups' language
Armenia	1995	8	Group-based (v): recognition of subordinate groups' cultural customs<br>Group-blind (h): proportional representation; mutual veto
Armenia	2005	12	Group-based (v): recognition of subordinate groups' religion; religious authorities
Armenia	2016	1	Group-based (h): proportional representation<br>Group-based (v): recognition of subordinate groups' religion; language
Georgia	1992	11	Group-based (v): recognition of subordinate groups' language; cultural customs; culturally-distinct education; right to native language education; native language use in government<br>Group-blind (h): mutual veto<br>Group-blind (v): policy scope (military, police)
Georgia	1995	9	Group-based (v): recognition of subordinate groups' religion; language; native language use in courts; religious authorities<br>Group-blind (h): proportional representation<br>Group-blind (v): institutional depth; taxing autonomy
Georgia	2017	11	Group-blind (v): institutional depth; policy scope (economy, culture, welfare); taxing autonomy; regional legislature; regional executive
Finland	1996	1	Group-based (v): recognition of subordinate groups' ethnic identity; language; cultural customs; land rights; culturally-distinct education; native language use in

Table S1. Complete list of concessions.

country	year	month	type of concession
			government; national symbols<br>Group-blind (v): policy scope (culture)
Finland	1997	12	Group-blind (v): change in administrative boundaries
Guinea-Bissau	1992	1	Group-blind (h): mutual veto
Guinea-Bissau	2005	7	Group-blind (h): informal inclusion
Equatorial Guinea	2012	3	Group-based (h): proportional representation<br>Group-blind (h): mutual veto
The Gambia	1996	9	Group-based (v): recognition of subordinate groups' religion; cultural customs; religious law; religious authorities<br>Group-blind (h): mutual veto<br>Group-blind (v): institutional depth; policy scope (economy, culture); regional legislature; regional executive
Mali	1992	1	Group-blind (h): informal inclusion
Mali	1992	2	Group-based (h): proportional representation<br>Group-based (v): recognition of subordinate groups' religious symbols<br>Group-blind (h): proportional representation; mutual veto
Mali	1996	1	Group-based (v): recognition of subordinate groups' language
Senegal	2001	2	Group-based (v): recognition of subordinate groups' language; right to native language education; religious symbols
Benin	1990	3	Group-based (v): recognition of subordinate groups' ethnic identity; language; cultural customs; culturally-distinct judiciary systems; religious symbols<br>Group-blind (h): proportional representation; mutual veto
Benin	2006	4	Group-blind (h): informal inclusion
Mauritania	1991	8	Group-based (h): proportional representation<br>Group-based (v): recognition of subordinate groups' ethnic identity; language; religious NGOs; religious symbols<br>Group-blind (h): proportional representation; mutual veto
Niger	1991	2	Group-blind (h): informal inclusion
Niger	1993	1	Group-based (v): recognition of subordinate groups' ethnic identity; religion; language; ethnic NGOs; right to native language media; national symbols; religious symbols<br>Group-blind (h): proportional representation; mutual veto
Niger	1999	9	Group-based (v): recognition of subordinate groups' ethnic identity; ethnic NGOs; national symbols<br>Group-blind (h): informal inclusion
Niger	2009	9	Group-based (v): recognition of subordinate groups' cultural customs
Niger	2011	2	Group-blind (h): informal inclusion
Cote d'Ivoire	2000	1	Group-blind (h): informal inclusion
Cote d'Ivoire	2000	8	Group-blind (h): mutual veto
Cote d'Ivoire	2002	4	Group-blind (h): informal inclusion
Cote d'Ivoire	2016	12	Group-based (h): proportional representation<br>Group-based (v): recognition of subordinate groups' ethnic NGOs; cultural customs; ethnic institutions<br>Group-blind (h): proportional representation
Guinea	1991	1	Group-based (v): recognition of subordinate groups' language<br>Group-blind (h): proportional representation; mutual veto
Ghana	1992	5	Group-based (h): proportional representation<br>Group-based (v): recognition of subordinate groups' ethnic identity; cultural customs; land rights; culturally-distinct judiciary systems; ethnic institutions; religious authorities; native language use in government; religious symbols<br>Group-blind (h): proportional representation; mutual veto<br>Group-blind (v): institutional depth; regional legislature
Togo	1990	4	Group-blind (h): informal inclusion
Togo	1992	11	Group-based (v): recognition of subordinate groups' cultural customs; ethnic institutions; religious authorities; religious symbols

Table S1. Complete list of concessions**.**

country	year	month	type of concession
Togo	2005	3	Group-blind (h): mutual veto<br>Group-blind (v): institutional depth; regional legislature<br>Group-blind (h): informal inclusion
Cameroon	1996	2	Group-based (h): proportional representation<br>Group-based (v): recognition of subordinate groups' ethnic identity; ethnic institutions<br>Group-blind (h): proportional representation<br>Group-blind (v): institutional depth; policy scope (economy, culture, welfare); regional legislature; regional executive
Nigeria	1998	5	Group-blind (h): informal inclusion
Nigeria	1999	6	Group-based (h): grand coalition; proportional representation; mutual veto<br>Group-based (v): recognition of subordinate groups' ethnic NGOs<br>Group-blind (h): grand coalition; proportional representation; mutual veto<br>Group-blind (v): institutional depth; policy scope (economy, culture, welfare, judiciary, institutions, residual powers); regional legislature; regional executive
Nigeria	2007	5	Group-blind (h): informal inclusion
Gabon	1991	3	Group-based (h): proportional representation<br>Group-blind (h): proportional representation; mutual veto
Central African Republic	1993	1	Group-based (h): proportional representation<br>Group-blind (h): proportional representation
Central African Republic	1993	2	Group-blind (h): informal inclusion
Central African Republic	1994	5	Group-blind (h): mutual veto
Central African Republic	2003	4	Group-blind (h): informal inclusion
Central African Republic	2005	1	Group-based (v): recognition of subordinate groups' religious symbols<br>Group-blind (h): mutual veto
Central African Republic	2009	1	Group-blind (h): informal inclusion
Central African Republic	2013	4	Group-based (h): grand coalition<br>Group-based (v): recognition of subordinate groups' ethnic party
Central African Republic	2015	11	Group-based (h): grand coalition; proportional representation<br>Group-based (v): recognition of subordinate groups' ethnic identity; cultural customs; ethnic institutions<br>Group-blind (h): grand coalition; proportional representation; mutual veto<br>Group-blind (v): institutional depth; regional legislature
Congo	1991	1	Group-blind (h): informal inclusion
Congo	1991	7	Group-based (v): recognition of subordinate groups' ethnic identity; language; cultural customs; culturally-distinct judiciary systems
Congo	1992	4	Group-based (h): proportional representation<br>Group-blind (h): proportional representation; mutual veto
Congo	1995	1	Group-blind (h): informal inclusion
Congo	2015	12	Group-based (v): recognition of subordinate groups' ethnic identity; ethnic institutions<br>Group-blind (h): mutual veto
Congo, DRC	1999	1	Group-blind (h): informal inclusion
Congo, DRC	2003	5	Group-based (h): grand coalition; proportional representation; mutual veto<br>Group-based (v): recognition of subordinate groups' ethnic party; ethnic identity; language<br>Group-blind (h): proportional representation; mutual veto<br>Group-blind (v): institutional depth
Congo, DRC	2006	1	Group-based (h): grand coalition<br>Group-based (v): recognition of subordinate groups' cultural customs; culturally-distinct judiciary systems; ethnic institutions; religious authorities; religious

Table S1. Complete list of concessions.

country	year	month	type of concession
			symbols<br>Group-blind (h): grand coalition; proportional representation; mutual veto<br>Group-blind (v): policy scope (economy, culture, welfare, police, judiciary); taxing autonomy; borrowing autonomy; regional legislature; regional executive
Congo, DRC	2015	1	Group-blind (h): informal inclusion
Congo, DRC	2015	2	Group-based (h): grand coalition; proportional representation<br>Group-blind (h): grand coalition<br>Group-blind (v): institutional depth; policy scope (economy, culture, welfare, police, judiciary); taxing autonomy; borrowing autonomy; regional legislature; regional executive
Congo, DRC	2017	1	Group-blind (h): informal inclusion
Uganda	2006	1	Group-based (v): recognition of subordinate groups' cultural customs; culturally-distinct judiciary systems; ethnic institutions
Kenya	1992	1	Group-blind (h): mutual veto
Kenya	1992	9	Group-based (h): grand coalition<br>Group-blind (h): grand coalition
Kenya	2003	1	Group-blind (h): informal inclusion
Kenya	2008	1	Group-blind (h): informal inclusion
Kenya	2008	3	Group-based (h): grand coalition
Kenya	2010	9	Group-based (h): grand coalition; proportional representation<br>Group-based (v): recognition of subordinate groups' cultural customs; religious authorities; religious symbols<br>Group-blind (h): grand coalition<br>Group-blind (v): institutional depth; policy scope (economy, culture, welfare); taxing autonomy; borrowing autonomy; regional legislature; regional executive
Tanzania	1992	6	Group-based (v): recognition of subordinate groups' native language use in government<br>Group-blind (h): mutual veto<br>Group-blind (v): institutional depth; regional legislature; regional executive
Burundi	1992	3	Group-based (h): grand coalition; proportional representation; mutual veto<br>Group-based (v): recognition of subordinate groups' language<br>Group-blind (h): proportional representation; mutual veto
Burundi	2004	11	Group-based (h): grand coalition; proportional representation; mutual veto<br>Group-based (v): recognition of subordinate groups' ethnic identity; native language use in government<br>Group-blind (h): grand coalition; proportional representation
Burundi	2018	7	Group-based (h): grand coalition; mutual veto
Rwanda	2003	7	Group-based (h): proportional representation<br>Group-blind (h): grand coalition; proportional representation; mutual veto<br>Group-blind (v): institutional depth
Rwanda	2010	7	Group-blind (h): grand coalition
Djibouti	1992	10	Group-based (v): recognition of subordinate groups' religion; religious authorities<br>Group-blind (h): mutual veto
Djibouti	1995	1	Group-blind (h): informal inclusion
Djibouti	2013	12	Group-blind (h): proportional representation
Ethiopia	1991	6	Group-blind (h): informal inclusion
Ethiopia	1991	8	Group-based (h): grand coalition; proportional representation<br>Group-based (v): recognition of subordinate groups' ethnic party; ethnic identity; cultural customs; culturally-distinct education; national symbols<br>Group-blind (v): policy scope (culture, secession)
Ethiopia	1995	1	Group-based (h): proportional representation; mutual veto<br>Group-based (v): recognition of subordinate groups' ethnic identity; language; cultural customs; religious law; ethnic institutions; native language use in government<br>Group-blind (h): grand coalition; mutual veto

Table S1. Complete list of concessions.

country	year	month	type of concession
Ethiopia	2013	1	Group-blind (v): institutional depth; policy scope (economy, culture, welfare, police, judiciary, institutions, residual powers, secession); taxing autonomy; borrowing autonomy; regional legislature; regional executive<br>Group-blind (h): informal inclusion
Angola	1992	10	Group-based (h): proportional representation<br>Group-based (v): recognition of subordinate groups' ethnic institutions<br>Group-blind (h): proportional representation<br>Group-blind (v): institutional depth; policy scope (economy); regional legislature
Angola	2010	2	Group-based (v): recognition of subordinate groups' culturally-distinct judiciary systems<br>Group-blind (v): taxing autonomy
Mozambique	1990	11	Group-based (v): recognition of subordinate groups' culturally-distinct judiciary systems
Mozambique	2005	1	Group-based (v): recognition of subordinate groups' ethnic institutions<br>Group-blind (h): proportional representation; mutual veto
Zambia	1991	9	Group-based (h): proportional representation<br>Group-based (v): recognition of subordinate groups' religion<br>Group-blind (h): proportional representation; mutual veto
Zimbabwe (Rhodesia)	1991	1	Group-based (v): recognition of subordinate groups' ethnic identity; religious symbols
Zimbabwe (Rhodesia)	2005	10	Group-based (h): proportional representation<br>Group-blind (h): proportional representation
Zimbabwe (Rhodesia)	2008	4	Group-blind (h): informal inclusion
Zimbabwe (Rhodesia)	2013	6	Group-based (h): grand coalition<br>Group-based (v): recognition of subordinate groups' religion; language; native language use in government<br>Group-blind (h): proportional representation<br>Group-blind (v): institutional depth; policy scope (economy); regional legislature
Malawi	1994	6	Group-based (h): proportional representation<br>Group-blind (h): proportional representation; mutual veto
South Africa	1994	1	Group-based (h): proportional representation<br>Group-based (v): recognition of subordinate groups' language; ethnic NGOs; culturally-distinct education; right to native language education; native language use in courts; native language use in government; national symbols<br>Group-blind (h): grand coalition; proportional representation<br>Group-blind (v): institutional depth; policy scope (economy, culture, welfare, police, institutions); taxing autonomy; borrowing autonomy; regional legislature; regional executive
South Africa	1994	5	Group-based (h): proportional representation<br>Group-based (v): recognition of subordinate groups' ethnic identity; religion; language; ethnic NGOs; cultural customs; right to native language education; native language use in courts; ethnic institutions; religious authorities; native language use in government
South Africa	1997	1	Group-based (v): recognition of subordinate groups' language; right to native language education; religious symbols<br>Group-blind (h): mutual veto<br>Group-blind (v): taxing autonomy; borrowing autonomy
South Africa	2007	12	Group-blind (v): change in administrative boundaries
South Africa	2013	12	Group-blind (v): change in administrative boundaries
Namibia	2014	12	Group-based (h): grand coalition<br>Group-blind (h): grand coalition
Madagascar	1992	9	Group-based (h): proportional representation<br>Group-based (v): recognition of subordinate groups' land rights; ethnic institutions<br>Group-blind (h): proportional representation; mutual veto<br>Group-blind (v): policy scope (police); taxing autonomy; borrowing autonomy

Table S1. Complete list of concessions.

country	year	month	type of concession
Madagascar	1995	12	Group-blind (v): regional executive
Madagascar	1998	5	Group-based (v): recognition of subordinate groups' religious authorities<br>Group-blind (h): proportional representation<br>Group-blind (v): policy scope (institutions, residual powers)
Madagascar	2009	9	Group-based (h): grand coalition; proportional representation<br>Group-based (v): recognition of subordinate groups' ethnic party
Comoros	1992	7	Group-based (h): proportional representation<br>Group-based (v): recognition of subordinate groups' language; separate religious schooling; religious authorities; religious symbols<br>Group-blind (v): policy scope (police); regional legislature
Comoros	2002	1	Group-based (h): grand coalition; proportional representation; mutual veto<br>Group-blind (v): institutional depth; policy scope (welfare, police, judiciary, institutions, residual powers); regional legislature; regional executive
Comoros	2009	12	Group-based (v): recognition of subordinate groups' religion
Comoros	2018	8	Group-based (v): recognition of subordinate groups' religious practices; separate religious schooling<br>Group-blind (h): mutual veto
Mauritius	1991	10	Group-blind (h): informal inclusion
Algeria	1990	12	Group-blind (h): proportional representation
Algeria	1997	1	Group-based (v): recognition of subordinate groups' ethnic identity; religious law<br>Group-blind (h): proportional representation
Algeria	2016	4	Group-based (v): recognition of subordinate groups' language; native language use in government
Libya	2011	3	Group-based (h): proportional representation<br>Group-based (v): recognition of subordinate groups' ethnic identity; language; cultural customs<br>Group-blind (h): proportional representation; mutual veto
Sudan	1998	7	Group-based (v): recognition of subordinate groups' ethnic identity; religion; language; religious practices; religious authorities; religious symbols<br>Group-blind (h): mutual veto<br>Group-blind (v): institutional depth; policy scope (economy, culture, welfare); borrowing autonomy; regional legislature; regional executive
Sudan	2005	8	Group-based (h): grand coalition; proportional representation; mutual veto<br>Group-based (v): recognition of subordinate groups' ethnic party; ethnic identity; cultural customs; religious practices; right to native language education; culturally-distinct judiciary systems; religious authorities; native language use in government<br>Group-blind (h): proportional representation<br>Group-blind (v): institutional depth; policy scope (military, police, judiciary, institutions, residual powers, secession); taxing autonomy; borrowing autonomy
Sudan	2011	8	Group-based (h): proportional representation<br>Group-blind (h): proportional representation<br>Group-blind (v): regional legislature; regional executive
Sudan	2012	1	Group-blind (h): proportional representation; mutual veto
Sudan	2015	2	Group-based (h): grand coalition; proportional representation<br>Group-based (v): recognition of subordinate groups' ethnic party; land rights<br>Group-blind (h): grand coalition<br>Group-blind (v): institutional depth; policy scope (economy, culture, welfare, institutions); taxing autonomy; borrowing autonomy; regional legislature; regional executive
Sudan	2016	12	Group-blind (v): institutional depth; policy scope (police, judiciary)
Iraq	2003	12	Group-based (v): recognition of subordinate groups' ethnic institutions
Iraq	2004	1	Group-blind (h): informal inclusion
Iraq	2004	4	Group-based (h): proportional representation; mutual veto<br>Group-based (v): recognition of subordinate groups' ethnic identity; language; cultural customs; right to native language education; religious authorities; native

Table S1. Complete list of concessions**.**

country	year	month	type of concession
			language use in government; linguistic symbols<br>Group-blind (h): grand coalition; mutual veto<br>Group-blind (v): institutional depth; policy scope (police, judiciary, residual powers, community); taxing autonomy; borrowing autonomy; regional legislature; regional executive
Iraq	2005	10	Group-based (v): recognition of subordinate groups' religion; religious practices; ethnic institutions; native language use in government; religious symbols<br>Group-blind (h): proportional representation<br>Group-blind (v): institutional depth; policy scope (culture, welfare)
Egypt	2007	4	Group-blind (v): institutional depth; regional legislature; regional executive
Egypt	2014	2	Group-based (h): proportional representation<br>Group-based (v): recognition of subordinate groups' religion<br>Group-blind (h): mutual veto
Syria	2012	3	Group-blind (h): mutual veto<br>Group-blind (v): institutional depth; regional legislature
Yemen	1991	6	Group-based (h): grand coalition<br>Group-based (v): recognition of subordinate groups' separate religious schooling; religious symbols<br>Group-blind (h): mutual veto
Yemen	1994	10	Group-based (v): recognition of subordinate groups' religious law
Kuwait	1992	1	Group-based (v): recognition of subordinate groups' religion; language; religious authorities; religious symbols<br>Group-blind (h): proportional representation; mutual veto
Bahrain	2002	3	Group-blind (h): mutual veto
Tajikistan	1994	12	Group-blind (h): mutual veto<br>Group-blind (v): taxing autonomy; borrowing autonomy
Tajikistan	1999	10	Group-based (h): proportional representation<br>Group-blind (h): proportional representation
Tajikistan	2000	12	Group-blind (v): change in administrative boundaries
Tajikistan	2010	12	Group-blind (v): change in administrative boundaries
Kyrgyzstan	1996	12	Group-based (v): recognition of subordinate groups' language<br>Group-blind (h): mutual veto
Kyrgyzstan	2000	12	Group-blind (h): proportional representation
Kyrgyzstan	2003	12	Group-based (v): recognition of subordinate groups' native language use in government
Kyrgyzstan	2007	11	Group-based (v): recognition of subordinate groups' ethnic identity; language<br>Group-blind (h): proportional representation; mutual veto
Kyrgyzstan	2010	7	Group-based (v): recognition of subordinate groups' right to native language education
Uzbekistan	2003	5	Group-based (h): proportional representation<br>Group-blind (h): proportional representation
Kazakhstan	1993	2	Group-blind (h): mutual veto
Kazakhstan	1994	1	Group-based (v): recognition of subordinate groups' language
Kazakhstan	1995	9	Group-based (h): proportional representation<br>Group-based (v): recognition of subordinate groups' native language use in government<br>Group-blind (h): proportional representation
Kazakhstan	2007	12	Group-blind (h): proportional representation
Mongolia	1990	3	Group-blind (h): mutual veto
Mongolia	1992	2	Group-based (v): recognition of subordinate groups' ethnic identity; language; religious symbols<br>Group-blind (h): mutual veto<br>Group-blind (v): institutional depth; policy scope (economy, judiciary); regional legislature; regional executive

Table S1. Complete list of concessions**.**

country	year	month	type of concession
Taiwan	1991	5	Group-based (h): proportional representation; mutual veto<br>Group-based (v): recognition of subordinate groups' ethnic identity<br>Group-blind (h): proportional representation; mutual veto
Taiwan	1992	6	Group-based (v): recognition of subordinate groups' ethnic NGOs; cultural customs; culturally-distinct education; ethnic institutions
Taiwan	1997	12	Group-based (v): recognition of subordinate groups' language
India	1992	12	Group-based (v): recognition of subordinate groups' language; right to native language education
India	2003	9	Group-based (v): recognition of subordinate groups' ethnic identity<br>Group-blind (v): institutional depth; policy scope (economy, culture, welfare, police, institutions); taxing autonomy; regional legislature
India	2011	12	Group-based (v): recognition of subordinate groups' ethnic identity
India	2017	12	Group-blind (v): change in administrative boundaries
Pakistan	1992	12	Group-blind (v): institutional depth; policy scope (economy, culture, welfare, police, residual powers); taxing autonomy; borrowing autonomy; regional legislature
Pakistan	2002	8	Group-based (h): proportional representation<br>Group-based (v): recognition of subordinate groups' ethnic identity; religion; language; religious NGOs; cultural customs; religious practices; separate religious schooling; religious law; religious authorities; native language use in government; religious symbols<br>Group-blind (h): proportional representation; mutual veto<br>Group-blind (v): institutional depth; policy scope (economy, culture, welfare, police, judiciary, institutions, residual powers, community); taxing autonomy; borrowing autonomy; regional legislature; regional executive
Pakistan	2008	3	Group-blind (h): informal inclusion
Pakistan	2010	5	Group-based (h): proportional representation
Bangladesh	2011	7	Group-based (v): recognition of subordinate groups' ethnic identity; cultural customs
Myanmar	2011	1	Group-based (h): proportional representation<br>Group-based (v): recognition of subordinate groups' ethnic identity<br>Group-blind (v): institutional depth; policy scope (welfare); regional legislature; regional executive
Nepal	1990	12	Group-based (v): recognition of subordinate groups' ethnic identity; cultural customs; culturally-distinct education; right to native language education<br>Group-blind (h): grand coalition; mutual veto
Nepal	2001	12	Group-based (v): recognition of subordinate groups' ethnic identity
Nepal	2008	5	Group-blind (h): informal inclusion
Nepal	2009	1	Group-based (h): mutual veto<br>Group-based (v): recognition of subordinate groups' linguistic symbols<br>Group-blind (h): proportional representation; mutual veto
Nepal	2015	10	Group-based (h): grand coalition; proportional representation; mutual veto<br>Group-based (v): recognition of subordinate groups' land rights; culturally-distinct education; ethnic institutions<br>Group-blind (v): institutional depth; policy scope (economy, culture, welfare, police); taxing autonomy; borrowing autonomy; regional legislature
Thailand	1992	1	Group-blind (v): institutional depth; taxing autonomy
Thailand	1997	11	Group-based (h): proportional representation<br>Group-based (v): recognition of subordinate groups' religious authorities<br>Group-blind (h): proportional representation<br>Group-blind (v): policy scope (economy, culture); regional legislature; regional executive
Cambodia	1993	6	Group-blind (h): informal inclusion
Cambodia	1993	10	Group-based (h): proportional representation<br>Group-based (v): recognition of subordinate groups' separate religious schooling;

Table S1. Complete list of concessions.

country	year	month	type of concession
			religious authorities<br>Group-blind (h): proportional representation; mutual veto
Malaysia	2001	2	Group-based (h): grand coalition; proportional representation<br>Group-blind (v): change in administrative boundaries
Philippines	1991	12	Group-blind (v): borrowing autonomy
Philippines	1997	1	Group-blind (h): proportional representation
Indonesia	1993	12	Group-based (v): recognition of subordinate groups' culturally-distinct judiciary systems
Indonesia	1999	11	Group-based (h): mutual veto
Indonesia	2000	9	Group-based (h): mutual veto<br>Group-based (v): recognition of subordinate groups' ethnic identity; culturally-distinct judiciary systems
Indonesia	2001	12	Group-based (h): proportional representation<br>Group-based (v): recognition of subordinate groups' religious law<br>Group-blind (v): institutional depth; policy scope (culture, police, judiciary); taxing autonomy; borrowing autonomy; regional legislature; regional executive
Indonesia	2002	9	Group-based (h): grand coalition<br>Group-based (v): recognition of subordinate groups' ethnic identity<br>Group-blind (h): grand coalition
Indonesia	2004	5	Group-blind (h): informal inclusion
Indonesia	2004	12	Group-blind (v): policy scope (economy, welfare, residual powers)
Indonesia	2005	12	Group-based (h): proportional representation<br>Group-blind (h): proportional representation
Indonesia	2006	12	Group-blind (v): institutional depth
Indonesia	2014	5	Group-blind (h): informal inclusion
Indonesia	2016	1	Group-blind (h): informal inclusion
Papua New Guinea	1996	12	Group-based (v): recognition of subordinate groups' ethnic identity
Papua New Guinea	2001	9	Group-blind (v): policy scope (welfare, police, judiciary, institutions, community, secession); taxing autonomy; borrowing autonomy
Papua New Guinea	2004	12	Group-blind (v): institutional depth

S2: Dominant group anti-government protest and violence directed against subordinate groups

S2.1 Ethnic attribution: general principles and sources

To identity instances of anti-government protests and anti-minority violence involving members of the politically-dominant community, I combined two datasets: First, the Mass Mobilization Data Project (MMD) (Clark and Regan 2016), which covers anti-government protests involving at least 50 people in 162 countries around the world since 1990. Second, the UCDP Non-State Conflict Dataset (version 20.1, cf. Pettersson et al. 2019; Sundberg et al. 2012), which covers instances of non-state violence which resulted in at least 25 casualties in at least one year since 1989. To set up my analyses, I connected the events covered by both datasets with the Ethnic Power Relations Dataset (EPR, Vogt et al. 2015).

In both cases, I predominantly focused on the **ethnic identity of the actors or their members** to link them to the EPR dataset, **rather than on official statements** by involved actors, as is commonly done for datasets linking ethnic mobilization, such as civil violence, to ethnic groups (Vogt et al. 2015). This is for two reasons: First, information on official claims was not systematically available for either dataset. Second, participants in a significant share of protest and communal conflict events, especially those initiated by informally organized identity groups, rarely make such official statements in the first place. Indeed, as discussed in the main article text above, they may express the dominant group's interests and oppose ethnic accommodation while publicly using the language of individual equality. Hence, I preferred to err on the side of inclusivity and adopted an encompassing coding that is based on ascriptive actor identities, as opposed to public ideological claims and statements.

I relied on three sources to ascertain the ethnic identity of actors in both datasets: First, wherever possible, I used the ACD2EPR dataset, which identifies ethnically based organizations, based on leadership claims and patterns in member recruitment (Vogt et al. 2015). I predominantly used this dataset for connecting formally-organized actors in the UCDP Non-State Conflict Dataset (NSV, Pettersson et al. 2019; Sundberg et al. 2012) with EPR. Second, for the attribution of other conflict actors, especially informally-organized ones (supporters of political parties and identity groups), I consulted the UCDP conflict encyclopedia,[6] which contains short narratives on all actors and conflicts in the NSV data. Third, for identifying ethnically based protest events, I relied on existing, text-based information provided by MMD (Clark and Regan 2016): the protest group identity and the short newspaper article extracts, both of which MMD provides for each protest event.

S2.2: Identification of ethnic actors

Depending on the dataset and type of actor/event, I proceeded as follows:

- For formally-organized actors in the UCDP NSV dataset and for protests where MMD states they were organized by a formal organization, I proceeded as follows:
    - First, I relied on existing information by the **ACD2EPR dataset, wherever possible** (Vogt et al. 2015). This codes, for each organization that ever engaged in state-based violence against the national government between 1946 and 2021, whether it is connected to a specific ethnic group in the EPR list through organizational claims and recruitment. Using this data, it was possible to account for the ethnic identity of all UCDP actors that were simultaneously, previously, or subsequently engaged in rebellion against the national government. For example, the Gerkan Aceh Merdeka (Free Aceh Movement), which repeatedly committed violence against Aceh's highland minorities, is linked to the "Acehnese" EPR group in the ACD2EPR data. Similarly, the Mohajir Qaumi Movement (MQM), which engaged in one-sided and communal violence against Sindhi in Sindh, is linked to the "Mohajir" EPR group in the ACD2EPR data.
    - Second, if the organization was not included in ACD2EPR, I **manually checked whether its membership was ethnically delimited in membership** by relying on the actor/dyad/conflict narratives provided by the UCDP conflict encyclopedia, the news article extracts provided by MMD, and complementary web research where this was necessary. For example, in South Africa, protests organized by the African National Congress were linked to the "Blacks" EPR group, whereas protests organized by the Afrikaner Resistance Movement were linked to the "Afrikaner" EPR group, owing to these organizations clear ethnic ties to these groups. Similarly, the South Lebanon Army (SLA) (*side b* in Lebanon's Hezbollah-SLA conflict in the UCDP NSV dataset) was linked to the "Maronites" EPR group, as the UCDP conflict encyclopedia stated that it had its roots in efforts by Maronite villages to coordinate armed militias.[7] Finally, the Forces of Amanullah Khan, which repeatedly engaged in non-state violence against forces of other warlords, were linked to the "Pashto" EPR group, as the UCDP conflict encyclopedia stated that Khan was

[6] Available at <https://ucdp.uu.se/>.
[7] Cf. <https://ucdp.uu.se/actor/827>.

of Pashtun ethnicity and that one of his stated goals has been to fight for the inclusion of Pashtuns in Afghanistan's western provinces.[8]

- Second, if the actor in question was an **informally organized conflict actor** that "shares a common identification along ethnic, clan, religious, national or tribal lines" (as per the codebook in the UCDP Non-State Violence Dataset) or where MMD does not identify a formal organization as the initiator of a protest event, I manually coded links between the actors/events and the EPR groups by name. Similar to Wig and Kromrey (2018), I proceeded as follows:
  - First, I linked actors that have a **direct (identical) match** in the EPR dataset. For example, protests with protester identity "Basques" and conflict events involving the Masalit (*side a* in Sudan's Masalit-Rizeigat Abbala conflict in the UCDP NSV dataset) were linked to the EPR group of the same name.
  - Second, I looked for direct matches with identifying names that are similar but **spelled differently**. For example, protests with protester identity "Flemish" were linked to the "Flemings" group in the EPR dataset, while the Karimojong (*side a* in Uganda's Karimojong-Pkoto conflict in the UCDP NSV dataset) were linked to the "Karamojong" group in the EPR dataset.
  - Third, I use databases on ethnic groups, such as the EPR encyclopedia,[9] the actor/dyad/conflict narratives provided by the UCDP conflict encyclopedia, and the news article extracts provided by MMD, to find **synonyms** for these names and match by synonyms. For example, protests involving the "Dalits" were linked to the "Scheduled Castes" EPR group. Similarly, the Fulani (*side b* in Mali's Dogon-Fulani conflict in the UCDP NSV dataset) were linked to the group encompassing the "Peul" in the EPR dataset.
  - Fourth, I match actors to the larger **umbrella EPR group** of which they are subgroups using the same databases. For example, the Kikuyu (*side b* in Kenya's Kalenjin-Kikuyu conflict in the UCDP NSV dataset) were linked to the "Kikuyu-Meru-Emb" group in the EPR dataset.
  - Vice-versa, I match actors that refer to a more encompassing cultural or phenotypical identity group to the various EPR groups that are encompassed by this wider identity. For this purpose, I consulted the EPR-Ethnic Dimensions Dataset (EPR-ED, Vogt et al. 2015), which lists the three largest linguistic, religious, and racial segments of each ethnic group. I coded a link where the cultural identity in question covers at least 50% of the group membership. For example, protests with protester identities "Hindus", "Hindu hardliners", "Hindu nationalists" and "Extremist Hindu groups" were linked to all EPR groups with a majority-Hindu religious affiliation (most notably the "Hindi (Non SC/ST OBCs)"), as were non-state conflict events that included the "Hindus" (*side a* in India's Hindus-Muslims conflict in the UCDP NSV dataset).
  - Finally, I match actors to a **closely related EPR group** if such connections are explicitly stated in these databases. For example, the Dogon (*side a* in Mali's Dogon-Fulani conflict in the UCDP NSV dataset) were a dark-skinned tribe and henced matched to Mali's "Blacks (Mande, Peul, Voltaic etc.)" EPR group.

S2.3: Validation (non-state violence)

To validate my coding, I compare my non-state violence data with two existing datasets that ethnically attributed the UCDP Non-State Violence to the EPR group list in a similar fashion: First, with the **group year-level data assembled by Hillesund (2019)** (which captures whether a group was involved in communal violence in a given year); and, second, with **group-level data collected by Wig & Kromrey (2018)** (which codes whether a group was ever involved in communal violence between 1989 and 2013). In both cases, the data only cover the subset of African countries and exclusively focus on informally organized instances of non-state violence. Hence, I report the correlations of these external measures with the group-year/group level equivalents of my communal violence variable for the same sample, respectively, focusing on informally organized instances thereof. A limitation of this comparison is that the EPR group classification has changed in several countries since these data have been collected, which means some groups coded by Hillesund (2019) and Wig & Kromrey (2018) lack equivalents in my data or may be mismatched. Nevertheless, and reassuringly, my measures are strongly correlated with both, yielding a Pearson's correlation coefficient of **r = 0.61** (correlation at the group year-level with data by Hillesund 2019) and **r = 0.66** (correlation at the group-level with data by Wig & Kromrey 2018).

To provide further transparency, and enable follow-up analyses, the supplementary material contains a full list of all ethnically-attributed protest events and non-state violence incidents.

[8] Cf. <https://ucdp.uu.se/actor/377>.
[9] Available at <https://growup.ethz.ch/>.

S3: Dominant nationalist parties

S3.1: Identification of dominant nationalist parties

To ascertain the existence of at least one dominant nationalist party, I predominantly relied on the V-Parties Dataset, V2.0 (Lindberg et al. 2022). This dataset identifies all parties that have achieved at least 5% of the vote share at a given election. Using this dataset as the foundation, I identified any party that satisfied any of the two following conditions:

- First, according to the V-Parties dataset's expert assessment, it was both in favor of majoritarian rule (*v2paminor_ord* ≤ 2; it "always", "usually", or "half of the time" argues that the will of the majority should determine policy even if such policy violates minority rights) and did *not* oppose the promotion of a specific social group's or the nation's cultural superiority (*v2paculsup_ord* ≥ 2; it "strongly promotes" or "promotes" the cultural superiority of a specific social group or the nation as a whole or is "ambiguous" and does not take a specific position on the cultural superiority of a specific social group or the nation as a whole).
- Second, it was a governing party or officially supported the government (*v2pagovsup* ≤ 2) during a time when an ethnic group in the EPR dataset (Vogt et al. 2015) attained "dominance" or "monopoly rule" status and was simultaneously in favor of majoritarian rule according to the V-Parties dataset's expert assessment (*v2paminor_ord* ≤ 2; it "always", "usually", or "half of the time" argues that the will of the majority should determine policy even if such policy violates minority rights).

From this initial list of "suspect" parties, I excluded 41 parties that ostensibly represented a politically subordinate ethnic subordinate group (e.g., "Quebec Bloc" in Canada or "Republican Left of Catalonia" in Spain), were clearly non-ethnic (e.g., "Greens of Serbia" in Serbia), or were not a party in the formally organized sense (e.g., the "Maronite Christians", "Sunni Moslem", "Greek Orthodox", "Shia Moslem", and "Greek Catholics" in Lebanon).

For each of the identified parties, I then coded the years of existence (founding and dissolution years, if applicable), relying on the information provided by the PartyFacts dataset.[10]

The result of these procedures is a list of a total of 313 dominant nationalist parties, along with their years of existence, in the 113 countries in my sample between 1990 and 2018. In data supplement S3.2, I provide a complete list of all identified dominant nationalist parties in my sample. The supplementary material contains a list of all of these parties, along with their V-Party ID.

[10] Available at < https://partyfacts.herokuapp.com/>.

S3.2: Complete list of dominant nationalist parties

Canada
Reform Party of Canada (1993-1999)
Guatemala
National Liberation Movement Party (1990-1993)
El Salvador
National Conciliation [Coalition] Party (1990-2018)
Nationalist Republican Alliance (1990-2018)
Colombia
Colombian Conservative Party (1990-2018)
National Salvation Movement (1991-1993)
Coalition (1998-2005)
National Integration Party (2010-2013)
Guyana
People's Progressive Party (1990-2018)
People's National Congress – Reform (1990-2014)
Working People's Alliance (2011-2014)
A Partnership for National Unity (2015-2018)
Ecuador
Social Christian Party [Movement] (1990-2018)
Radical Liberal Party (1990-1993)
Brazil
Social Liberal Party (2018)
Brazilian Republican Party (2014-2018)
Progressive Party / Brazilian Progressive Party (PPB) (2010)
Paraguay
National Republican Association – Colorado Party (1990-2018)
National Union of Ethical Citizens (2003-2017)
United Kingdom
United Kingdom Independence Party (2001-2018)
Conservatives (2017-2018)
Belgium
Flemish Block / Interest (1991-2013)
France
National Front (1990-2018)
Switzerland
Swiss People's Party (1990-2018)
Automobile Party / Freedom Party of Switzerland (1991-1994)
Catholic Conservative / Christian Democratic Peoples Party (2011-2018)
Poland
Christian National Union (1991-1996)
Confederation for Independent Poland (1991-1996)
Self-Defense of the Republic Poland (2001-2006)
Movement for the Reconstruction of Poland (1997-2000)
Law and Justice (2001-2018)
League of Polish Families (2001-2006)
Kukiz'15 (2015-2018)
Austria
Freedom Party of Austria (1990-2018)
Hungary
Independent Smallholders Party (1988) (1990-2001)
Hungarian Justice and Life Party (1998-2001)
Hungarian Democratic Forum (1994-1997)
Fidesz – Hungarian Civic Alliance (2002-2018)
Movement for a Better Hungary (2010-2018)
Christian Democratic People's Party (2006-2018)
Slovakia
Movement for a Democratic Slovakia (1994-2009)
Slovak National Party (1994-2018)
People's Party Our Slovakia (2016-2018)
Direction -- Social Democracy (2012-2018)
We are family – Boris Kollar (2016-2018)
Italy
North League (1992-2018)
Italian Social Movement / Italian Social Movement-National Right (1990-1993)
National Alliance (1994-2007)
Brothers of Italy (2013-2018)
Albania
Party of Labour / Albanian Workers' Party (1990)
North Macedonia
Internal Macedonian Revolutionary Organization – Democratic Party for Macedonian National Unity (1994-2018)
VMRO-People's Party (2006-2007)
Croatia
Croatian Party of Rights (1992-2006)
Serbia and Montenegro / Serbia
Serbian Radical Party (1992-2018)
Serbian Renewal Movement (1990-2018)
Socialist Party of Serbia (1990-2018)
Party of United Pensioners of Serbia (2012-2018)
Democratic Party of Serbia (1993-2018)
Strength of Serbia Movement – BK (2016-2018)
Doors (2016-2018)
United Serbia (2012-2018)
Yugoslav Left (1997-1999)
Serbian Progressive Party (2014-2018)
Movement of Socialists (2014-2018)
Let's get Serbia moving - Tomislav Nikolic (2012-2013)
New Serbia (2014-2018)
Social Democratic Party of Serbia (2014-2018)
People's Peasant Party (2016-2018)
Bosnia and Herzegovina
Party of Democratic Action (1996-2018)
Alliance of Independent Social Democrats (2002-2018)
Kosovo
Self-determination (2010-2018)
Democratic League of Dardania (2007-2009)
Albanian Christian Democratic Party of Kosovo (2007-2009)
Slovenia
Slovenian National Party (1992-2010)
Slovenian Democratic Party (1990-2018)
Slovenian People's Party (1992-2013)
New Slovenia -- Christian People's Party (1996-2018)
Greece
People's Association / Golden Dawn (2012-2018)
Popular Orthodox Rally (2009-2011)
Independent Greeks (2012-2014)
Cyprus

Democratic Rally (1990-2000)
Democratic Party (1990-2018)
Movement for Social Democracy EDEK (1990-2018)
Solidarity Movement (2016-2018)
Bulgaria
National Union Attack (2005-2013)
National Front for the Salvation of Bulgaria (2014-2018)
Bulgaria Without Censorship (2014-2016)
Moldova
Christian Democratic People's Party (1994-2008)
Unity Movement for Equality in Rights (1994-1997)
Socialist Party of Moldova (1994-1997)
Liberal Party (2009-2018)
Bloc of Peasants and Intellectuals (1994-1997)
Party of Democratic Forces (1998-2000)
Party of Communists of the Republic of Moldova (2001-2018)
Electoral Bloc ""Democratic Moldova"" (2005-2008)
Romania
Greater Romania Party (2000-2007)
Conservative Party / Romanian Humanist Party (2000-2015)
Romanian National Unity Party (1992-1995)
Social Democratic Party (1992-1999)
People's Party – Dan Diaconescu (2012-2015)
Russia
Liberal Democratic Party of Russia (1993-2018)
Our Home - Russia (1995-1998)
Unity (1999-2002)
Motherland -- National Patriotic Union (2003-2006)
United Russia (2003-2018)
Estonia
Estonian People's Party (1990-2018)
Ukraine
People's Movement of Ukraine (1990-2001)
All Ukrainian Union ""Freedom"" / Svoboda (2014-2018)
All Ukrainian Union ""Fatherland"" (2014-2018)
Our Ukraine – People's Self-Defense Bloc (2002-2011)
Yulia Tymoshenko Bloc (2006-2011)
Radical Party of Oleh Lyashko (2014-2018)
Self Reliance Party (2014-2018)
People's Front (2014-2018)
European Solidarity / Petro Poroshenko Bloc (2014-2018)
Azerbaijan
Equality Party (2005-2009)
Azerbaijan Popular Front (1990-2004)
New Azerbaijan Party (1995-2018)
Equatorial Guinea
Democratic Party of Equatorial Guinea (1990-2018)
Mauritania
Rally for Democracy and Unity (1992-2005)
Democratic [and Social] Republican Party for Renewal (1992-2012)
Union for the Republic (2013-2018)
Niger
Rally for Democracy and Progress / National Union of Independents for Democratic Renewal (1996-2010)
Party for People's Dignity (1996)
Social Democratic Rally (2010)
Togo
Rally of the Togolese People (1990-1991)
Pan-African Democratic Party (2018)
Union for the Republic (2015)
Union of Forces for Change (2015)
Central African Republic
Central African Democratic Rally (1990-1997)
Democratic Republic of the Congo
Popular Movement of the Revolution (1990-2002)
People's Party for Reconstruction and Democracy (2003-2018)
Burundi
Union for National Progress (1990-2009)
Front for Democracy in Burundi (1993-2014)
National Council for the Defense of Democracy – Front for the Defense of Democracy (2005-2018)
Rwanda
National Republican Movement for Democracy and Development (1990-1994)
Djibouti
Front for the Restoration of Unity and Democracy (1997-2002)
Ethiopia
Coalition for Unity and Democracy (2005-2009)
Angola
National Union for the Total Independence of Angola (1992-2007)
Zimbabwe
Zimbabwe African National Union-Ndongo (1990-1999)
Zimbabwe African National Union – Patriotic Front (1990-2018)
Malawi
Malawi Congress Party (1990-1993)
South Africa
Economic Freedom Fighters (2014-2018)
Comoros
National Front for Justice (1997)
National Rally for Development (1997)
Convention for the Renewal of the Comoros (2018)
Democratic Rally of the Comoros (2018)
Algeria
Movement for the Society of Peace (1997-2018)
Libya
Arab Socialist Union of Libya (1990-2011)
Sudan
National Islamic Front (1990-1999)
National Congress Party (2000-2018)
Iran
Islamic Republican Party (1990-1991)
Combatant Clergy Association (1992-2003)
Combatant Clerics of Tehran (1996-1999)
Servants of Iran's Construction (1996-1999)
Islamic Iran Participation Front (2000-2003)

Principlists / Iranian Conservatives (2004-2015)
Front of Islamic Revolution Stability (2016-2018)
Islamic Coalition Party (2016-2018)
Society of Devotees of the Islamic Revolution (2016-2018)
Society of Pathseekers of the Islamic Revolution (2016-2018)

Turkey

Motherland Party (1990-2006)
Nationalist Movement [Action] Party (1990-2018)
True Path Party (1995-1998)
Republican Peoples Party (1999-2006)
Justice and Development Party (2011-2018)

Iraq

Islamic Dawa Party (2005-2018)
Islamic Supreme Council of Iraq (2005-2017)
Sadrist Movement (2010-2018)
Iraqi National Congress (2014-2017)
Badr Organization (2005-2018)
Speakers of the Truth (2018)
National Movement for Development and Reform (2014-2017)
Al-Hadba (2014-2017)

Egypt

National Democratic Party (1990-2010)
New Wafd Party (1990-2018)
Nation's Future Party (2015-2018)

Syria

National Progressive Front (1990-2015)
Arab Socialist Ba'ath Party – Syria Region (1990-2018)
Arab Socialist Union (1990-2018)
Arabic Democratic Union Party (2016-2018)
Democratic Socialist Unionist Party (2016-2018)
National Covenant Party (2016-2018)
Socialist Unionist Party (2016-2018)
Syrian Communist Party (Bakdash) (2016-2018)
Syrian Communist Party (Unified) (2016-2018)
Syrian Social Nationalist Party (2016-2018)

Jordan

Islamic Action Front (1990-2009)

Kuwait

Popular Action Bloc (2008)

Bahrain

Al-Wefaq National Islamic Societ (2006-2013)

Tajikistan

People's Democratic Party of Tajikistan (1995-2018)
Islamic Renaissance Party of Tajikistan (2000-2009)

Kyrgyzstan

Ata Zhurt / Fatherland (2010-2014)
Union of Democratic Forces (2004)
My Country Party of Action (2004)
Democratic Women's Party of Kyrgyzstan (2004)
Political Party of Afghan War Veterans (2004)
United Kyrgyzstan (2010-2014)

Uzbekistan

People's Democratic Party of Uzbekistan (1990-2018)
Justice Social Democratic Party (1995-2018)
Self-Sacrifice National Democratic Party (1999-2008)
Uzbekistan Liberal Democratic Party (2004-2018)
Uzbekistan National Revival Democratic Party (2004-2018)

Kazakhstan

People's Union of Kazakhstan Unity (1994)

Mongolia

Democratic Party (1990-2018)
Mongolian People's [Revolutionary] Party (1990-2018)
Mongolian Social Democratic Party (1990-1999)
Mongolian National Progress Party (1990-1995)
Motherland Party / Mongolian Democratic New Socialist Party (2004-2007)
United Party (1992-1995)
Mongolian National Democratic Party (2012-2015)
Civil Will-Green Party (2004-2015)
Mongolian People's Revolutionary Party (2012-2015)

Taiwan

Nationalist Party (1990-2018)
Democratic Progressive Party (1990-2018)
Chinese / New Party (1995-1997)
People First Party (2001-2007)
Taiwan Solidarity Union (2001-2007)

Japan

Liberal Democratic Party (1990-2018)
Japan Restoration [Innovation] Party (2012-2013)

India

Indian People's Party (1990-2018)

Pakistan

Muttahida Quami Movement /Worship of the Real Group (1990-2017)
Islamic Democratic Alliance (1990-1992)
United Council of Action (2002-2018)

Bangladesh

Bangladesh People's League (1990-2018)
Islam Conference Bangladesh (1991-1995)
Bangladesh Nationalist Party (1990-2013)
Combined Opposition Party (1990)

Burma/Myanmar

Union Solidarity and Development Party (1993-2018)
National Unity Party (1990-2014)

Sri Lanka

United National Party (1990-2009)
Sri Lanka Freedom Party (1990-2018)
People's Liberation Front (2000-2014)
Democratic United National Front (2010-2014)
National Heritage Party (2004-2009)

Nepal

National Democratic Party (1999-2018)

Thailand

Thai Nation Party / Chart Thai Party (1990-2010)
National Development Party (1992-2004)
Thai Citizens' Party / Prachakorn Thai Party (1990-1991)
Bhumjaithai Party (2011-2018)

Cambodia

Cambodian Peoples' Party / Kampuchean People's

Malaysia

United Malays National Organisation (1990-2018)
Pan-Malaysian Islamic Party (1990-2018)
United Bumiputera Heritage Party (1990-2018)
Spirit of 46 Malay Party (1990-1998)
Malaysian United Indigenous Party (2018)
National Trust Party / Malaysia Workers' Party (2018)

Indonesia

Party of the Functional Groups (1990-2018)
Prosperous Justice Party / Welfare and Justice Party (2004-2018)
United Development Party (1990-2018)
National Awakening Party (1999-2018)
National Mandate Party (2004-2018)
People's Conscience Party (2014-2018)
Great Indonesia Movement Party (2009-2018)
