# Supplementary material for: Ethnic Accommodation and the Backlash From Dominant Groups
Source: J Conflict Resolut. 2025 May 22;70(2-3):359–86. doi: 10.1177/00220027251343836 (PMC12782309; doi:10.1177/00220027251343836)
Supplement: Supplemental Material - Ethnic Accommodation and the Backlash From Dominant Groups [file sj-zip-3-jcr-10.1177_00220027251343836.zip › tables/results/app1.1_reverse.html]

**Reverse analysis: Dominant group mobilization, subordinate group civil violence and protests, and the provision of concessions to subordinate groups.**

|  | | | |
|  | **Model 1 Concession** | **Model 2 Concession (group-based)** | **Model 3 Concession (group-blind)** |
|  | | | |
| Dominant group mobilization events (last 5y, log) | 0.081 | -0.069 | 0.125 |
|  | (0.089) | (0.131) | (0.150) |
| Subordinate group civil violence incidents (last 5y, log) | 0.009 | 0.005 | 0.002 |
|  | (0.053) | (0.076) | (0.068) |
| Subordinate group protests (last 5y, log) | 0.019 | 0.322\* | -0.026 |
|  | (0.088) | (0.140) | (0.150) |
| DN party | -0.144 | 0.152 | -0.369 |
|  | (0.272) | (0.370) | (0.574) |
| DN party in government | 0.043 | 0.020 | -0.542 |
|  | (0.259) | (0.385) | (0.397) |
| Months to next election (log) | -0.212\*\*\* | -0.191\*\* | -0.224\*\* |
|  | (0.042) | (0.059) | (0.070) |
| Battle deaths (last 10y, log) | -0.038 | -0.167 | 0.104 |
|  | (0.117) | (0.183) | (0.163) |
| Democracy level | -0.130 | -0.733 | 2.062 |
|  | (1.100) | (1.317) | (1.442) |
| Abs. size (log) | -0.059 | -0.020 | 0.265 |
|  | (0.389) | (0.547) | (0.567) |
| GDP p.c. (log) | 0.350 | -1.139 | -0.318 |
|  | (0.486) | (0.874) | (0.569) |
| GDP growth | -2.604\*\* | -1.690 | -2.145\*\* |
|  | (0.806) | (1.090) | (0.815) |
| no\_conc\_months\_l1 | -0.082\*\*\* |  |  |
|  | (0.007) |  |  |
| I(no\_conc\_months\_l12) | 0.001\*\*\* |  |  |
|  | (0.0001) |  |  |
| I(no\_conc\_months\_l13) | -0.00000\*\*\* |  |  |
|  | (0.00000) |  |  |
| no\_conc\_symbolic\_months\_l1 |  | -0.080\*\*\* |  |
|  |  | (0.010) |  |
| I(no\_conc\_symbolic\_months\_l12) |  | 0.001\*\*\* |  |
|  |  | (0.0001) |  |
| I(no\_conc\_symbolic\_months\_l13) |  | -0.00000\*\* |  |
|  |  | (0.00000) |  |
| no\_conc\_nonsymbolic\_months\_l1 |  |  | -0.094\*\*\* |
|  |  |  | (0.007) |
| I(no\_conc\_nonsymbolic\_months\_l12) |  |  | 0.001\*\*\* |
|  |  |  | (0.0001) |
| I(no\_conc\_nonsymbolic\_months\_l13) |  |  | -0.00000\*\*\* |
|  |  |  | (0.00000) |
| Constant | -10.928\* | -6.304 | -16.874\*\* |
|  | (5.495) | (9.326) | (6.346) |
| Country-FE | yes | yes | yes |
| Year-FE | yes | yes | yes |
| N | 32723 | 32723 | 32723 |
| Log Likelihood | -3624.979 | -1983.553 | -1636.398 |
| AIC | 7567.958 | 4285.107 | 3590.796 |
|  | | | |
| † p<0.1; \* p<0.05; \*\* p<0.01; \*\*\* p<0.001; country-clustered SE's in parentheses; cubic terms for country-wise years without (group-based/group-blind) concessions included but not reported. | | | |
