# Supplementary material for: Ethnic Accommodation and the Backlash From Dominant Groups
Source: J Conflict Resolut. 2025 May 22;70(2-3):359–86. doi: 10.1177/00220027251343836 (PMC12782309; doi:10.1177/00220027251343836)
Supplement: Supplemental Material - Ethnic Accommodation and the Backlash From Dominant Groups [file sj-zip-3-jcr-10.1177_00220027251343836.zip › tables/results/app1.3_1.4_endo.html]

**Ethnic accommodation and the number of mobilization events involving the dominant group [post-conflict vs. stable contexts subsample analyses].**

|  | | | | |
|  | **Model 1** | **Model 2** | **Model 3** | **Model 4** |
|  | | | | |
| conc\_sum\_close3 | 0.182\*\* |  | 0.164\*\* |  |
|  | (0.056) |  | (0.060) |  |
| conc\_sum\_symbolic\_close3 |  | 0.371\*\* |  | 0.276 |
|  |  | (0.142) |  | (0.176) |
| conc\_sum\_nonsymbolic\_close3 |  | -0.013 |  | 0.055 |
|  |  | (0.138) |  | (0.171) |
| mnm\_party\_exists | 0.088 | 0.082 | 0.111 | 0.107 |
|  | (0.288) | (0.285) | (0.168) | (0.167) |
| mnm\_partygov | 0.097 | 0.102 | 0.146 | 0.145 |
|  | (0.147) | (0.147) | (0.158) | (0.158) |
| ltt\_nextelec | -0.026 | -0.027 | -0.130\*\* | -0.133\*\* |
|  | (0.027) | (0.027) | (0.047) | (0.047) |
| month\_protest2\_nsc\_forward3\_close3 | 0.481\*\*\* | 0.480\*\*\* | 0.306\*\* | 0.307\*\* |
|  | (0.101) | (0.101) | (0.110) | (0.110) |
| month\_viol\_civil\_nsc\_forward3\_close3 | 0.288† | 0.287† | 0.143 | 0.139 |
|  | (0.155) | (0.159) | (0.167) | (0.164) |
| ld10\_bdead\_allc | 0.233\* | 0.233\* | -0.169\* | -0.166\* |
|  | (0.112) | (0.112) | (0.079) | (0.076) |
| vdem\_libdem | -0.069 | -0.031 | -2.050\*\* | -2.055\*\* |
|  | (0.451) | (0.460) | (0.746) | (0.746) |
| lsize\_abs | 0.413 | 0.413 | -0.102 | -0.100 |
|  | (0.363) | (0.356) | (0.324) | (0.324) |
| lgdppc | -0.322 | -0.316 | -0.751 | -0.736 |
|  | (0.334) | (0.332) | (0.543) | (0.538) |
| gdppc\_change | -0.534 | -0.548 | -1.212\* | -1.240\* |
|  | (0.721) | (0.724) | (0.530) | (0.538) |
| Constant | 0.200 | 0.124 | 7.879 | 7.744 |
|  | (3.748) | (3.714) | (5.722) | (5.686) |
| N | 28065 | 28065 | 10150 | 10150 |
| Log Likelihood | -16577.600 | -16574.130 | -6877.510 | -6876.623 |
| theta | 0.445\*\*\* (0.015) | 0.446\*\*\* (0.015) | 0.484\*\*\* (0.024) | 0.484\*\*\* (0.024) |
| AIC | 33445.200 | 33440.260 | 13933.020 | 13933.250 |
|  | | | | |
| † p<0.1; \* p<0.05; \*\* p<0.01; \*\*\* p<0.001; country-clustered SE's in parentheses; cubic terms for group-wise months without mobilization included but not reported. | | | | |
