# Supplementary material for: Ethnic Accommodation and the Backlash From Dominant Groups
Source: J Conflict Resolut. 2025 May 22;70(2-3):359–86. doi: 10.1177/00220027251343836 (PMC12782309; doi:10.1177/00220027251343836)
Supplement: Supplemental Material - Ethnic Accommodation and the Backlash From Dominant Groups [file sj-zip-3-jcr-10.1177_00220027251343836.zip › tables/results/app2.1_controls_shift.html]

**Ethnic accommodation and the number of mobilization events involving the dominant group [Additional controls for major constitutional and political shfits].**

|  | | | | |
|  | **Model 1** | **Model 2** | **Model 3** | **Model 4** |
|  | | | | |
| Concession number | 0.141\*\* | 0.081 |  |  |
|  | (0.053) | (0.080) |  |  |
| Concession number x DN party |  | 0.101 |  |  |
|  |  | (0.084) |  |  |
| Concession number (group-based) |  |  | 0.267\* | 0.061 |
|  |  |  | (0.104) | (0.130) |
| Concession number (group-based) x DN party |  |  |  | 0.332† |
|  |  |  |  | (0.193) |
| Concession number (group-blind) |  |  | 0.025 | 0.106 |
|  |  |  | (0.115) | (0.133) |
| Concession number (group-blind) x DN party |  |  |  | -0.131 |
|  |  |  |  | (0.200) |
| DN party | 0.088 | 0.075 | 0.087 | 0.075 |
|  | (0.165) | (0.164) | (0.165) | (0.162) |
| DN party in government | 0.044 | 0.050 | 0.046 | 0.052 |
|  | (0.092) | (0.092) | (0.093) | (0.094) |
| Months to next election (log) | -0.061\* | -0.061\* | -0.062\* | -0.062\* |
|  | (0.025) | (0.025) | (0.025) | (0.025) |
| Recent subordinate group protest | 0.380\*\*\* | 0.380\*\*\* | 0.380\*\*\* | 0.382\*\*\* |
|  | (0.082) | (0.082) | (0.082) | (0.082) |
| Recent civil violence | 0.112 | 0.110 | 0.110 | 0.109 |
|  | (0.119) | (0.118) | (0.118) | (0.117) |
| Battle deaths (last 10y, log) | 0.063 | 0.065 | 0.065 | 0.068 |
|  | (0.073) | (0.072) | (0.072) | (0.072) |
| Democracy level | -0.399 | -0.401 | -0.382 | -0.400 |
|  | (0.320) | (0.323) | (0.326) | (0.322) |
| Abs. size (log) | 0.210 | 0.212 | 0.211 | 0.220 |
|  | (0.185) | (0.184) | (0.184) | (0.181) |
| GDP p.c. (log) | -0.212 | -0.214 | -0.203 | -0.205 |
|  | (0.298) | (0.299) | (0.297) | (0.297) |
| GDP growth | -0.892† | -0.881† | -0.915† | -0.912† |
|  | (0.499) | (0.500) | (0.502) | (0.505) |
| Regional DG mobilization events (log) | 0.026 | 0.039 | 0.002 | 0.017 |
|  | (0.206) | (0.208) | (0.201) | (0.201) |
| month\_election\_shock3\_close3 | 0.021 | 0.023 | 0.023 | 0.029 |
|  | (0.064) | (0.064) | (0.064) | (0.064) |
| month\_coup\_shock3\_close3 | 0.232 | 0.241 | 0.265 | 0.231 |
|  | (0.269) | (0.272) | (0.272) | (0.271) |
| pcf\_shock\_close3 | 0.310\* | 0.312\* | 0.309\* | 0.305\* |
|  | (0.151) | (0.150) | (0.152) | (0.151) |
| month\_govsup\_change\_shock3\_close3 | -0.221† | -0.222† | -0.219† | -0.225† |
|  | (0.117) | (0.117) | (0.118) | (0.118) |
| lunreg\_backlash\_no | 0.067\* | 0.067\* | 0.067\* | 0.067\* |
|  | (0.029) | (0.029) | (0.029) | (0.029) |
| Constant | 0.535 | 0.562 | 0.445 | 0.453 |
|  | (3.241) | (3.244) | (3.224) | (3.216) |
| Country-FE | yes | yes | yes | yes |
| Year-FE | yes | yes | yes | yes |
| Wald-Test Chisq |  |  |  |  |
| Joint sig. int. concession |  | 0.002\*\* |  |  |
| Joint sig. int. concession (group-based) |  |  |  | 0.004\*\* |
| Joint sig. int. concession (group-blind) |  |  |  | 0.883 |
| N | 38130 | 38130 | 38130 | 38130 |
| Log Likelihood | -23025.740 | -23024.160 | -23023.170 | -23019.520 |
| theta | 0.515\*\*\* (0.015) | 0.515\*\*\* (0.015) | 0.515\*\*\* (0.015) | 0.516\*\*\* (0.015) |
| AIC | 46397.490 | 46396.320 | 46394.340 | 46391.040 |
|  | | | | |
| † p<0.1; \* p<0.05; \*\* p<0.01; \*\*\* p<0.001; country-clustered SE's in parentheses; cubic terms for group-wise months without mobilization included but not reported. | | | | |
