# Supplementary material for: Ethnic Accommodation and the Backlash From Dominant Groups
Source: J Conflict Resolut. 2025 May 22;70(2-3):359–86. doi: 10.1177/00220027251343836 (PMC12782309; doi:10.1177/00220027251343836)
Supplement: Supplemental Material - Ethnic Accommodation and the Backlash From Dominant Groups [file sj-zip-3-jcr-10.1177_00220027251343836.zip › tables/results/app2.2_protest_psi.html]

**Ethnic accommodation and the number of mobilization events involving the dominant group [protests related to institutions and minority rights].**

|  | | | | |
|  | **Model 1** | **Model 2** | **Model 3** | **Model 4** |
|  | | | | |
| Concession number | 0.327\*\*\* | 0.134\* |  |  |
|  | (0.076) | (0.063) |  |  |
| Concession number x DN party |  | 0.494\*\*\* |  |  |
|  |  | (0.117) |  |  |
| Concession number (group-based) |  |  | 0.399 | -0.180 |
|  |  |  | (0.264) | (0.191) |
| Concession number (group-based) x DN party |  |  |  | 1.066\*\* |
|  |  |  |  | (0.337) |
| Concession number (group-blind) |  |  | 0.252 | 0.444\* |
|  |  |  | (0.308) | (0.204) |
| Concession number (group-blind) x DN party |  |  |  | -0.088 |
|  |  |  |  | (0.373) |
| DN party | -0.235 | -0.446 | -0.238 | -0.450 |
|  | (0.596) | (0.601) | (0.588) | (0.569) |
| DN party in government | -0.387 | -0.295 | -0.383 | -0.264 |
|  | (0.285) | (0.260) | (0.286) | (0.271) |
| Months to next election (log) | -0.019 | -0.033 | -0.022 | -0.041 |
|  | (0.076) | (0.074) | (0.080) | (0.078) |
| Recent subordinate group protest | 1.110\*\*\* | 1.106\*\*\* | 1.113\*\*\* | 1.115\*\*\* |
|  | (0.210) | (0.205) | (0.211) | (0.199) |
| Recent civil violence | -0.074 | -0.128 | -0.084 | -0.102 |
|  | (0.395) | (0.378) | (0.381) | (0.379) |
| Battle deaths (last 10y, log) | 0.291\* | 0.299\* | 0.290\* | 0.295\* |
|  | (0.137) | (0.138) | (0.137) | (0.136) |
| Democracy level | -0.261 | -0.480 | -0.281 | -0.487 |
|  | (1.485) | (1.355) | (1.435) | (1.308) |
| Abs. size (log) | -0.381 | -0.436 | -0.382 | -0.429 |
|  | (0.618) | (0.610) | (0.618) | (0.613) |
| GDP p.c. (log) | -1.790\* | -1.976\* | -1.775\* | -1.950\* |
|  | (0.884) | (0.877) | (0.889) | (0.870) |
| GDP growth | -0.028 | 0.054 | -0.018 | -0.046 |
|  | (0.952) | (1.058) | (0.949) | (1.116) |
| Regional DG mobilization events (log) | 0.115\* | 0.117\* | 0.115\* | 0.115\* |
|  | (0.053) | (0.055) | (0.054) | (0.055) |
| Constant | 15.441 | 17.706† | 15.324 | 17.443† |
|  | (9.616) | (9.582) | (9.692) | (9.497) |
| Country-FE | yes | yes | yes | yes |
| Year-FE | yes | yes | yes | yes |
| Wald-Test Chisq |  |  |  |  |
| Joint sig. int. concession |  | 0\*\*\* |  |  |
| Joint sig. int. concession (group-based) |  |  |  | 0\*\*\* |
| Joint sig. int. concession (group-blind) |  |  |  | 0.273 |
| N | 38130 | 38130 | 38130 | 38130 |
| Log Likelihood | -5162.261 | -5120.317 | -5161.658 | -5111.404 |
| AIC | 10660.520 | 10578.640 | 10661.320 | 10564.810 |
|  | | | | |
| † p<0.1; \* p<0.05; \*\* p<0.01; \*\*\* p<0.001; country-clustered SE's in parentheses; cubic terms for group-wise months without mobilization included but not reported. | | | | |
