# Supplementary material for: Ethnic Accommodation and the Backlash From Dominant Groups
Source: J Conflict Resolut. 2025 May 22;70(2-3):359–86. doi: 10.1177/00220027251343836 (PMC12782309; doi:10.1177/00220027251343836)
Supplement: Supplemental Material - Ethnic Accommodation and the Backlash From Dominant Groups [file sj-zip-3-jcr-10.1177_00220027251343836.zip › tables/results/app2.3_minviol.html]

**Ethnic accommodation and the number of mobilization events involving the dominant group [Alternative dependent variable: anti-government protests].**

|  | | | | |
|  | **Model 1** | **Model 2** | **Model 3** | **Model 4** |
|  | | | | |
| Concession number | 0.084† | 0.070 |  |  |
|  | (0.045) | (0.069) |  |  |
| Concession number x DN party |  | 0.025 |  |  |
|  |  | (0.080) |  |  |
| Concession number (group-based) |  |  | 0.145 | 0.088 |
|  |  |  | (0.107) | (0.125) |
| Concession number (group-based) x DN party |  |  |  | 0.100 |
|  |  |  |  | (0.185) |
| Concession number (group-blind) |  |  | 0.025 | 0.053 |
|  |  |  | (0.108) | (0.129) |
| Concession number (group-blind) x DN party |  |  |  | -0.048 |
|  |  |  |  | (0.202) |
| DN party | 0.179 | 0.176 | 0.180 | 0.177 |
|  | (0.158) | (0.158) | (0.158) | (0.157) |
| DN party in government | 0.070 | 0.071 | 0.070 | 0.072 |
|  | (0.103) | (0.103) | (0.103) | (0.103) |
| Months to next election (log) | -0.062\*\* | -0.062\*\* | -0.063\*\* | -0.063\*\* |
|  | (0.023) | (0.023) | (0.023) | (0.023) |
| Recent subordinate group protest | 0.338\*\*\* | 0.338\*\*\* | 0.338\*\*\* | 0.339\*\*\* |
|  | (0.094) | (0.094) | (0.094) | (0.094) |
| Recent civil violence | 0.072 | 0.073 | 0.072 | 0.072 |
|  | (0.106) | (0.106) | (0.106) | (0.106) |
| Battle deaths (last 10y, log) | 0.066 | 0.067 | 0.067 | 0.068 |
|  | (0.061) | (0.061) | (0.061) | (0.062) |
| Democracy level | -0.606† | -0.606† | -0.596† | -0.600† |
|  | (0.350) | (0.352) | (0.352) | (0.351) |
| Abs. size (log) | 0.459† | 0.459† | 0.459† | 0.462† |
|  | (0.256) | (0.255) | (0.254) | (0.254) |
| GDP p.c. (log) | 0.153 | 0.152 | 0.155 | 0.155 |
|  | (0.182) | (0.181) | (0.181) | (0.181) |
| GDP growth | -1.071\* | -1.070\* | -1.077\* | -1.078\* |
|  | (0.446) | (0.446) | (0.446) | (0.448) |
| Regional DG mobilization events (log) | 0.069\* | 0.069\* | 0.069\* | 0.069\* |
|  | (0.030) | (0.030) | (0.030) | (0.030) |
| Constant | -3.861† | -3.856† | -3.893† | -3.894† |
|  | (2.175) | (2.174) | (2.166) | (2.163) |
| Country-FE | yes | yes | yes | yes |
| Year-FE | yes | yes | yes | yes |
| Wald-Test Chisq |  |  |  |  |
| Joint sig. int. concession |  | 0.074† |  |  |
| Joint sig. int. concession (group-based) |  |  |  | 0.218 |
| Joint sig. int. concession (group-blind) |  |  |  | 0.978 |
| N | 38130 | 38130 | 38130 | 38130 |
| Log Likelihood | -21727.690 | -21727.600 | -21727.120 | -21726.830 |
| theta | 0.539\*\*\* (0.017) | 0.539\*\*\* (0.017) | 0.539\*\*\* (0.017) | 0.539\*\*\* (0.017) |
| AIC | 43791.370 | 43793.200 | 43792.240 | 43795.650 |
|  | | | | |
| † p<0.1; \* p<0.05; \*\* p<0.01; \*\*\* p<0.001; country-clustered SE's in parentheses; cubic terms for group-wise months without mobilization included but not reported. | | | | |
