# Supplementary material for: Ethnic Accommodation and the Backlash From Dominant Groups
Source: J Conflict Resolut. 2025 May 22;70(2-3):359–86. doi: 10.1177/00220027251343836 (PMC12782309; doi:10.1177/00220027251343836)
Supplement: Supplemental Material - Ethnic Accommodation and the Backlash From Dominant Groups [file sj-zip-3-jcr-10.1177_00220027251343836.zip › tables/results/app2.4_conch.html]

**Ethnic accommodation and the number of mobilization events involving the dominant group [horizontal concessions].**

|  | | | | |
|  | **Model 1** | **Model 2** | **Model 3** | **Model 4** |
|  | | | | |
| Concession number |  | -0.017 |  |  |
|  |  | (0.186) |  |  |
| Concession number x DN party |  |  |  | 0.185 |
|  |  |  |  | (0.349) |
| Concession number (group-based) |  |  |  | -0.177 |
|  |  |  |  | (0.264) |
| Concession number (group-based) x DN party | 0.085 | 0.086 | 0.084 | 0.088 |
|  | (0.167) | (0.167) | (0.167) | (0.167) |
| Concession number (group-blind) | 0.034 | 0.034 | 0.036 | 0.037 |
|  | (0.093) | (0.093) | (0.093) | (0.093) |
| Concession number (group-blind) x DN party | 0.173† | 0.182 |  |  |
|  | (0.092) | (0.143) |  |  |
| DN party |  |  | 0.338† | 0.224 |
|  |  |  | (0.191) | (0.198) |
| DN party in government |  |  | 0.066 | 0.159 |
|  |  |  | (0.130) | (0.178) |
| Months to next election (log) | -0.062\*\* | -0.062\*\* | -0.063\*\* | -0.063\*\* |
|  | (0.023) | (0.023) | (0.023) | (0.023) |
| Recent subordinate group protest | 0.389\*\*\* | 0.389\*\*\* | 0.388\*\*\* | 0.388\*\*\* |
|  | (0.083) | (0.083) | (0.082) | (0.083) |
| Recent civil violence | 0.143 | 0.144 | 0.144 | 0.145 |
|  | (0.124) | (0.124) | (0.123) | (0.123) |
| Battle deaths (last 10y, log) | 0.065 | 0.065 | 0.066 | 0.066 |
|  | (0.072) | (0.072) | (0.071) | (0.071) |
| Democracy level | -0.400 | -0.399 | -0.407 | -0.405 |
|  | (0.332) | (0.332) | (0.330) | (0.331) |
| Abs. size (log) | 0.209 | 0.209 | 0.208 | 0.207 |
|  | (0.184) | (0.184) | (0.184) | (0.184) |
| GDP p.c. (log) | -0.224 | -0.224 | -0.219 | -0.217 |
|  | (0.301) | (0.301) | (0.299) | (0.299) |
| GDP growth | -0.961† | -0.962† | -0.983† | -0.991\* |
|  | (0.502) | (0.502) | (0.503) | (0.502) |
| Regional DG mobilization events (log) | 0.066\* | 0.066\* | 0.067\* | 0.067\* |
|  | (0.029) | (0.029) | (0.029) | (0.029) |
| Constant | 0.687 | 0.683 | 0.653 | 0.629 |
|  | (3.270) | (3.272) | (3.254) | (3.258) |
| Country-FE | yes | yes | yes | yes |
| Year-FE | yes | yes | yes | yes |
| Wald-Test Chisq |  |  |  |  |
| Joint sig. int. concession |  | 0.164 |  |  |
| Joint sig. int. concession (group-based) |  |  |  | 0.143 |
| Joint sig. int. concession (group-blind) |  |  |  | 0.925 |
| N | 38130 | 38130 | 38130 | 38130 |
| Log Likelihood | -23046.340 | -23046.330 | -23044.930 | -23044.260 |
| theta | 0.511\*\*\* (0.014) | 0.511\*\*\* (0.014) | 0.512\*\*\* (0.014) | 0.512\*\*\* (0.014) |
| AIC | 46428.680 | 46430.650 | 46427.860 | 46430.510 |
|  | | | | |
| † p<0.1; \* p<0.05; \*\* p<0.01; \*\*\* p<0.001; country-clustered SE's in parentheses; cubic terms for group-wise months without mobilization included but not reported. | | | | |
