# Supplementary material for: Ethnic Accommodation and the Backlash From Dominant Groups
Source: J Conflict Resolut. 2025 May 22;70(2-3):359–86. doi: 10.1177/00220027251343836 (PMC12782309; doi:10.1177/00220027251343836)
Supplement: Supplemental Material - Ethnic Accommodation and the Backlash From Dominant Groups [file sj-zip-3-jcr-10.1177_00220027251343836.zip › tables/results/app2.4_concv.html]

**Ethnic accommodation and the number of mobilization events involving the dominant group [vertical concessions].**

|  | | | | |
|  | **Model 1** | **Model 2** | **Model 3** | **Model 4** |
|  | | | | |
| Concession number |  | 0.249\* |  |  |
|  |  | (0.115) |  |  |
| Concession number x DN party |  |  |  | 0.478† |
|  |  |  |  | (0.250) |
| Concession number (group-based) |  |  |  | 0.002 |
|  |  |  |  | (0.268) |
| Concession number (group-based) x DN party | 0.082 | 0.062 | 0.081 | 0.057 |
|  | (0.164) | (0.162) | (0.164) | (0.161) |
| Concession number (group-blind) | 0.042 | 0.052 | 0.042 | 0.053 |
|  | (0.093) | (0.093) | (0.093) | (0.094) |
| Concession number (group-blind) x DN party | 0.249\*\*\* | 0.104 |  |  |
|  | (0.063) | (0.076) |  |  |
| DN party |  |  | 0.367\*\* | 0.096 |
|  |  |  | (0.140) | (0.166) |
| DN party in government |  |  | 0.115 | 0.113 |
|  |  |  | (0.154) | (0.172) |
| Months to next election (log) | -0.060\*\* | -0.061\*\* | -0.060\*\* | -0.061\*\* |
|  | (0.022) | (0.022) | (0.023) | (0.022) |
| Recent subordinate group protest | 0.388\*\*\* | 0.388\*\*\* | 0.389\*\*\* | 0.390\*\*\* |
|  | (0.084) | (0.083) | (0.084) | (0.083) |
| Recent civil violence | 0.147 | 0.146 | 0.145 | 0.143 |
|  | (0.123) | (0.122) | (0.122) | (0.121) |
| Battle deaths (last 10y, log) | 0.062 | 0.064 | 0.063 | 0.066 |
|  | (0.073) | (0.072) | (0.072) | (0.072) |
| Democracy level | -0.414 | -0.405 | -0.387 | -0.395 |
|  | (0.321) | (0.326) | (0.328) | (0.325) |
| Abs. size (log) | 0.212 | 0.213 | 0.213 | 0.223 |
|  | (0.183) | (0.181) | (0.182) | (0.180) |
| GDP p.c. (log) | -0.226 | -0.229 | -0.221 | -0.225 |
|  | (0.297) | (0.298) | (0.298) | (0.298) |
| GDP growth | -0.946† | -0.927† | -0.945† | -0.930† |
|  | (0.502) | (0.503) | (0.505) | (0.508) |
| Regional DG mobilization events (log) | 0.067\* | 0.068\* | 0.066\* | 0.067\* |
|  | (0.029) | (0.029) | (0.029) | (0.029) |
| Constant | 0.703 | 0.734 | 0.626 | 0.660 |
|  | (3.237) | (3.244) | (3.242) | (3.240) |
| Country-FE | yes | yes | yes | yes |
| Year-FE | yes | yes | yes | yes |
| Wald-Test Chisq |  |  |  |  |
| Joint sig. int. concession |  | 0\*\*\* |  |  |
| Joint sig. int. concession (group-based) |  |  |  | 0.003\*\* |
| Joint sig. int. concession (group-blind) |  |  |  | 0.599 |
| N | 38130 | 38130 | 38130 | 38130 |
| Log Likelihood | -23036.050 | -23032.280 | -23034.420 | -23029.020 |
| theta | 0.513\*\*\* (0.014) | 0.514\*\*\* (0.014) | 0.513\*\*\* (0.014) | 0.515\*\*\* (0.015) |
| AIC | 46408.090 | 46402.560 | 46406.840 | 46400.050 |
|  | | | | |
| † p<0.1; \* p<0.05; \*\* p<0.01; \*\*\* p<0.001; country-clustered SE's in parentheses; cubic terms for group-wise months without mobilization included but not reported. | | | | |
