# Supplementary material for: Ethnic Accommodation and the Backlash From Dominant Groups
Source: J Conflict Resolut. 2025 May 22;70(2-3):359–86. doi: 10.1177/00220027251343836 (PMC12782309; doi:10.1177/00220027251343836)
Supplement: Supplemental Material - Ethnic Accommodation and the Backlash From Dominant Groups [file sj-zip-3-jcr-10.1177_00220027251343836.zip › tables/results/app3.1_bwd.html]

**Ethnic accommodation and the number of mobilization events involving the dominant group [only past concessions].**

|  | | | | |
|  | **Model 1** | **Model 2** | **Model 3** | **Model 4** |
|  | | | | |
| Concession number |  | 0.124 |  |  |
|  |  | (0.102) |  |  |
| Concession number x DN party |  |  |  | 0.322 |
|  |  |  |  | (0.211) |
| Concession number (group-based) |  |  |  | -0.085 |
|  |  |  |  | (0.247) |
| Concession number (group-based) x DN party | 0.118 | 0.110 | 0.118 | 0.111 |
|  | (0.197) | (0.197) | (0.196) | (0.197) |
| Concession number (group-blind) | 0.100 | 0.103 | 0.101 | 0.104 |
|  | (0.114) | (0.114) | (0.114) | (0.114) |
| Concession number (group-blind) x DN party | 0.080† | 0.008 |  |  |
|  | (0.048) | (0.083) |  |  |
| DN party |  |  | 0.197† | -0.001 |
|  |  |  | (0.108) | (0.164) |
| DN party in government |  |  | -0.037 | 0.016 |
|  |  |  | (0.114) | (0.204) |
| Months to next election (log) | -0.064\*\* | -0.064\*\* | -0.064\*\* | -0.064\*\* |
|  | (0.025) | (0.025) | (0.025) | (0.025) |
| Recent subordinate group protest | 0.504\*\*\* | 0.505\*\*\* | 0.503\*\*\* | 0.502\*\*\* |
|  | (0.091) | (0.091) | (0.091) | (0.091) |
| Recent civil violence | 0.205 | 0.205 | 0.205 | 0.204 |
|  | (0.152) | (0.152) | (0.151) | (0.151) |
| Battle deaths (last 10y, log) | 0.068 | 0.069 | 0.068 | 0.069 |
|  | (0.078) | (0.078) | (0.078) | (0.078) |
| Democracy level | -0.582 | -0.586 | -0.573 | -0.585 |
|  | (0.436) | (0.438) | (0.439) | (0.438) |
| Abs. size (log) | 0.262 | 0.261 | 0.261 | 0.265 |
|  | (0.229) | (0.228) | (0.229) | (0.227) |
| GDP p.c. (log) | -0.289 | -0.290 | -0.286 | -0.287 |
|  | (0.341) | (0.342) | (0.341) | (0.341) |
| GDP growth | -1.239\* | -1.238\* | -1.243\* | -1.242\* |
|  | (0.586) | (0.586) | (0.587) | (0.589) |
| Regional DG mobilization events (log) | 0.751 | 0.770 | 0.723 | 0.732 |
|  | (3.679) | (3.682) | (3.672) | (3.671) |
| Country-FE | yes | yes | yes | yes |
| Year-FE | yes | yes | yes | yes |
| Wald-Test Chisq |  |  |  |  |
| Joint sig. int. concession |  | 0.024\* |  |  |
| Joint sig. int. concession (group-based) |  |  |  | 0.012\* |
| Joint sig. int. concession (group-blind) |  |  |  | 0.603 |
| N | 38215 | 38215 | 38215 | 38215 |
| Log Likelihood | -23680.590 | -23679.350 | -23679.300 | -23677.330 |
| theta | 0.422\*\*\* (0.011) | 0.422\*\*\* (0.011) | 0.422\*\*\* (0.011) | 0.422\*\*\* (0.011) |
| AIC | 47689.180 | 47688.700 | 47688.610 | 47688.670 |
|  | | | | |
| † p<0.1; \* p<0.05; \*\* p<0.01; \*\*\* p<0.001; country-clustered SE's in parentheses; cubic terms for group-wise months without mobilization included but not reported. | | | | |
