# Supplementary material for: Ethnic Accommodation and the Backlash From Dominant Groups
Source: J Conflict Resolut. 2025 May 22;70(2-3):359–86. doi: 10.1177/00220027251343836 (PMC12782309; doi:10.1177/00220027251343836)
Supplement: Supplemental Material - Ethnic Accommodation and the Backlash From Dominant Groups [file sj-zip-3-jcr-10.1177_00220027251343836.zip › figures/margins/app3.1_bwd.pdf]

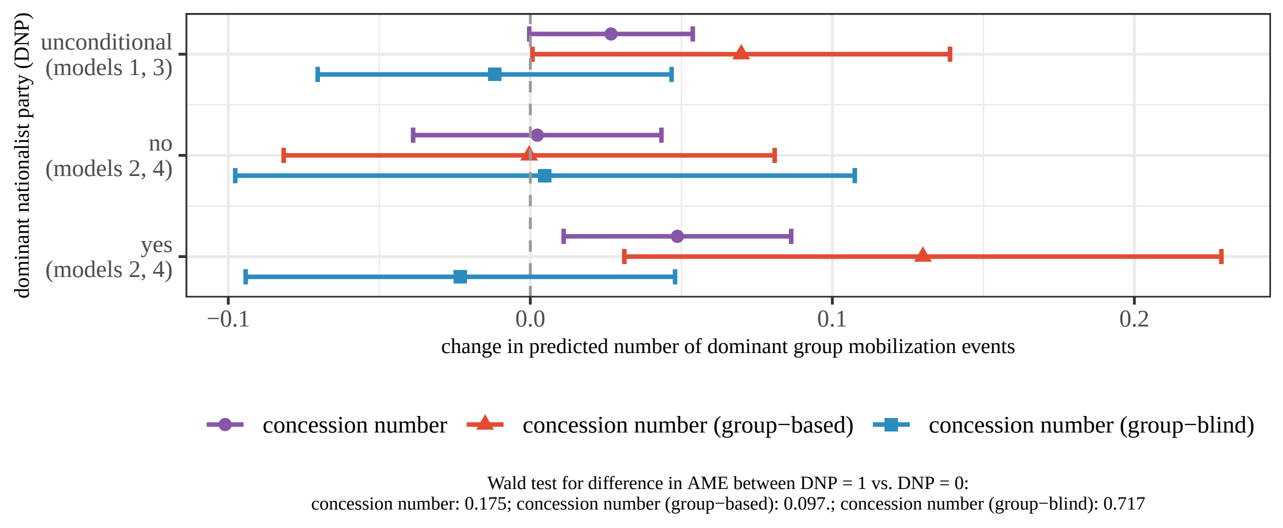
dominant nationalist party (DNP)
unconditional (models 1, 3)
no (models 2, 4)
yes (models 2, 4)
−0.1
0.0
0.1
0.2
change in predicted number of dominant group mobilization events
concession number
concession number (group−based)
concession number (group−blind)
Wald test for difference in AME between DNP = 1 vs. DNP = 0:
concession number: 0.175; concession number (group−based): 0.097.; concession number (group−blind): 0.717
