# Supplementary material for: Ethnic Accommodation and the Backlash From Dominant Groups
Source: J Conflict Resolut. 2025 May 22;70(2-3):359–86. doi: 10.1177/00220027251343836 (PMC12782309; doi:10.1177/00220027251343836)
Supplement: Supplemental Material - Ethnic Accommodation and the Backlash From Dominant Groups [file sj-zip-3-jcr-10.1177_00220027251343836.zip › tables/results/app3.1_dummy.html]

**Ethnic accommodation and the number of mobilization events involving the dominant group [dichotomous indicator].**

|  | | | | |
|  | **Model 1** | **Model 2** | **Model 3** | **Model 4** |
|  | | | | |
| Concession number | 0.318\*\*\* | 0.223† |  |  |
|  | (0.086) | (0.116) |  |  |
| Concession number x DN party |  | 0.170 |  |  |
|  |  | (0.163) |  |  |
| Concession number (group-based) |  |  | 0.383\*\* | 0.101 |
|  |  |  | (0.144) | (0.159) |
| Concession number (group-based) x DN party |  |  |  | 0.486† |
|  |  |  |  | (0.250) |
| Concession number (group-blind) |  |  | 0.054 | 0.154 |
|  |  |  | (0.124) | (0.150) |
| Concession number (group-blind) x DN party |  |  |  | -0.174 |
|  |  |  |  | (0.234) |
| DN party | 0.077 | 0.062 | 0.078 | 0.063 |
|  | (0.165) | (0.164) | (0.164) | (0.161) |
| DN party in government | 0.038 | 0.042 | 0.040 | 0.047 |
|  | (0.094) | (0.094) | (0.094) | (0.095) |
| Months to next election (log) | -0.059\*\* | -0.060\*\* | -0.061\*\* | -0.062\*\* |
|  | (0.023) | (0.023) | (0.023) | (0.023) |
| Recent subordinate group protest | 0.382\*\*\* | 0.383\*\*\* | 0.381\*\*\* | 0.382\*\*\* |
|  | (0.082) | (0.082) | (0.081) | (0.081) |
| Recent civil violence | 0.145 | 0.144 | 0.141 | 0.140 |
|  | (0.122) | (0.121) | (0.121) | (0.119) |
| Battle deaths (last 10y, log) | 0.063 | 0.063 | 0.066 | 0.068 |
|  | (0.073) | (0.072) | (0.072) | (0.072) |
| Democracy level | -0.428 | -0.426 | -0.381 | -0.395 |
|  | (0.320) | (0.323) | (0.330) | (0.326) |
| Abs. size (log) | 0.219 | 0.222 | 0.211 | 0.222 |
|  | (0.182) | (0.181) | (0.181) | (0.179) |
| GDP p.c. (log) | -0.222 | -0.223 | -0.212 | -0.217 |
|  | (0.302) | (0.302) | (0.299) | (0.298) |
| GDP growth | -0.949† | -0.942† | -0.940† | -0.927† |
|  | (0.508) | (0.508) | (0.508) | (0.509) |
| Regional DG mobilization events (log) | 0.066\* | 0.066\* | 0.067\* | 0.067\* |
|  | (0.029) | (0.029) | (0.029) | (0.029) |
| Constant | 0.637 | 0.650 | 0.536 | 0.581 |
|  | (3.281) | (3.282) | (3.249) | (3.239) |
| Country-FE | yes | yes | yes | yes |
| Year-FE | yes | yes | yes | yes |
| Wald-Test Chisq |  |  |  |  |
| Joint sig. int. concession |  | 0.001\*\* |  |  |
| Joint sig. int. concession (group-based) |  |  |  | 0.004\*\* |
| Joint sig. int. concession (group-blind) |  |  |  | 0.916 |
| N | 38130 | 38130 | 38130 | 38130 |
| Log Likelihood | -23033.600 | -23032.290 | -23033.110 | -23028.090 |
| theta | 0.513\*\*\* (0.014) | 0.514\*\*\* (0.014) | 0.514\*\*\* (0.014) | 0.515\*\*\* (0.015) |
| AIC | 46403.190 | 46402.580 | 46404.220 | 46398.180 |
|  | | | | |
| † p<0.1; \* p<0.05; \*\* p<0.01; \*\*\* p<0.001; country-clustered SE's in parentheses; cubic terms for group-wise months without mobilization included but not reported. | | | | |
