# Supplementary material for: Ethnic Accommodation and the Backlash From Dominant Groups
Source: J Conflict Resolut. 2025 May 22;70(2-3):359–86. doi: 10.1177/00220027251343836 (PMC12782309; doi:10.1177/00220027251343836)
Supplement: Supplemental Material - Ethnic Accommodation and the Backlash From Dominant Groups [file sj-zip-3-jcr-10.1177_00220027251343836.zip › tables/results/app3.1_ldnp.html]

**Ethnic accommodation and the number of mobilization events involving the dominant group [interaction with logged number of dominant nationalist parties].**

|  | | | | |
|  | **Model 1** | **Model 2** | **Model 3** | **Model 4** |
|  | | | | |
| Concession number | 0.148\*\*\* | 0.080 |  |  |
|  | (0.040) | (0.056) |  |  |
| Concession number x DN party |  | 0.121 |  |  |
|  |  | (0.075) |  |  |
| Concession number (group-based) |  |  | 0.268\* | 0.060 |
|  |  |  | (0.108) | (0.110) |
| Concession number (group-based) x DN party |  |  |  | 0.331\* |
|  |  |  |  | (0.159) |
| Concession number (group-blind) |  |  | 0.029 | 0.097 |
|  |  |  | (0.107) | (0.119) |
| Concession number (group-blind) x DN party |  |  |  | -0.093 |
|  |  |  |  | (0.178) |
| DN party | 0.053 | 0.058 | 0.054 | 0.059 |
|  | (0.092) | (0.092) | (0.093) | (0.093) |
| DN party in government | -0.060\*\* | -0.060\*\* | -0.061\*\* | -0.063\*\* |
|  | (0.023) | (0.022) | (0.023) | (0.022) |
| Months to next election (log) | 0.386\*\*\* | 0.386\*\*\* | 0.385\*\*\* | 0.386\*\*\* |
|  | (0.082) | (0.082) | (0.082) | (0.082) |
| Recent subordinate group protest | 0.146 | 0.142 | 0.144 | 0.140 |
|  | (0.123) | (0.121) | (0.121) | (0.119) |
| Recent civil violence | 0.065 | 0.066 | 0.066 | 0.070 |
|  | (0.072) | (0.072) | (0.072) | (0.071) |
| Battle deaths (last 10y, log) | 0.020 | 0.010 | 0.019 | 0.013 |
|  | (0.158) | (0.157) | (0.157) | (0.156) |
| Democracy level | -0.403 | -0.411 | -0.386 | -0.407 |
|  | (0.318) | (0.321) | (0.323) | (0.320) |
| Abs. size (log) | 0.203 | 0.209 | 0.204 | 0.217 |
|  | (0.183) | (0.182) | (0.182) | (0.180) |
| GDP p.c. (log) | -0.225 | -0.227 | -0.217 | -0.216 |
|  | (0.297) | (0.298) | (0.295) | (0.296) |
| GDP growth | -0.947† | -0.941† | -0.967† | -0.965† |
|  | (0.501) | (0.502) | (0.505) | (0.507) |
| Regional DG mobilization events (log) | 0.066\* | 0.066\* | 0.066\* | 0.067\* |
|  | (0.029) | (0.029) | (0.029) | (0.029) |
| Constant | 0.720 | 0.733 | 0.628 | 0.609 |
|  | (3.222) | (3.226) | (3.205) | (3.202) |
| Country-FE | yes | yes | yes | yes |
| Year-FE | yes | yes | yes | yes |
| Wald-Test Chisq |  |  |  |  |
| Joint sig. int. concession |  | 0\*\*\* |  |  |
| Joint sig. int. concession (group-based) |  |  |  | 0.003\*\* |
| Joint sig. int. concession (group-blind) |  |  |  | 0.978 |
| N | 38130 | 38130 | 38130 | 38130 |
| Log Likelihood | -23038.900 | -23036.300 | -23036.330 | -23031.300 |
| theta | 0.512\*\*\* (0.014) | 0.513\*\*\* (0.014) | 0.513\*\*\* (0.014) | 0.514\*\*\* (0.014) |
| AIC | 46413.790 | 46410.600 | 46410.670 | 46404.600 |
|  | | | | |
| † p<0.1; \* p<0.05; \*\* p<0.01; \*\*\* p<0.001; country-clustered SE's in parentheses; cubic terms for group-wise months without mobilization included but not reported. | | | | |
