# Supplementary material for: Ethnic Accommodation and the Backlash From Dominant Groups
Source: J Conflict Resolut. 2025 May 22;70(2-3):359–86. doi: 10.1177/00220027251343836 (PMC12782309; doi:10.1177/00220027251343836)
Supplement: Supplemental Material - Ethnic Accommodation and the Backlash From Dominant Groups [file sj-zip-3-jcr-10.1177_00220027251343836.zip › tables/results/app3.1_tw12.html]

**Ethnic accommodation and the number of mobilization events involving the dominant group [12-month time window].**

|  | | | | |
|  | **Model 1** | **Model 2** | **Model 3** | **Model 4** |
|  | | | | |
| Concession number | 0.077\*\* | 0.013 |  |  |
|  | (0.026) | (0.035) |  |  |
| Concession number x DN party |  | 0.107\* |  |  |
|  |  | (0.052) |  |  |
| Concession number (group-based) |  |  | 0.180\*\* | 0.045 |
|  |  |  | (0.068) | (0.098) |
| Concession number (group-based) x DN party |  |  |  | 0.211† |
|  |  |  |  | (0.123) |
| Concession number (group-blind) |  |  | -0.031 | -0.017 |
|  |  |  | (0.066) | (0.091) |
| Concession number (group-blind) x DN party |  |  |  | -0.014 |
|  |  |  |  | (0.116) |
| DN party | 0.079 | 0.033 | 0.087 | 0.048 |
|  | (0.166) | (0.164) | (0.164) | (0.163) |
| DN party in government | 0.045 | 0.060 | 0.046 | 0.060 |
|  | (0.093) | (0.094) | (0.093) | (0.094) |
| Months to next election (log) | -0.062\*\* | -0.063\*\* | -0.064\*\* | -0.064\*\* |
|  | (0.023) | (0.023) | (0.023) | (0.023) |
| Recent subordinate group protest | 0.392\*\*\* | 0.393\*\*\* | 0.390\*\*\* | 0.390\*\*\* |
|  | (0.083) | (0.082) | (0.083) | (0.082) |
| Recent civil violence | 0.153 | 0.151 | 0.144 | 0.140 |
|  | (0.122) | (0.118) | (0.118) | (0.114) |
| Battle deaths (last 10y, log) | 0.069 | 0.074 | 0.071 | 0.077 |
|  | (0.071) | (0.070) | (0.070) | (0.069) |
| Democracy level | -0.398 | -0.405 | -0.345 | -0.379 |
|  | (0.319) | (0.328) | (0.331) | (0.327) |
| Abs. size (log) | 0.213 | 0.221 | 0.218 | 0.235 |
|  | (0.180) | (0.177) | (0.177) | (0.173) |
| GDP p.c. (log) | -0.214 | -0.220 | -0.189 | -0.199 |
|  | (0.296) | (0.298) | (0.286) | (0.287) |
| GDP growth | -0.907† | -0.891† | -0.942† | -0.937† |
|  | (0.496) | (0.489) | (0.506) | (0.500) |
| Regional DG mobilization events (log) | 0.066\* | 0.066\* | 0.067\* | 0.067\* |
|  | (0.029) | (0.029) | (0.029) | (0.029) |
| Constant | 0.550 | 0.617 | 0.258 | 0.349 |
|  | (3.214) | (3.219) | (3.114) | (3.115) |
| Country-FE | yes | yes | yes | yes |
| Year-FE | yes | yes | yes | yes |
| Wald-Test Chisq |  |  |  |  |
| Joint sig. int. concession |  | 0.001\*\* |  |  |
| Joint sig. int. concession (group-based) |  |  |  | 0.001\*\* |
| Joint sig. int. concession (group-blind) |  |  |  | 0.702 |
| N | 38130 | 38130 | 38130 | 38130 |
| Log Likelihood | -23041.060 | -23035.860 | -23034.570 | -23028.470 |
| theta | 0.512\*\*\* (0.014) | 0.513\*\*\* (0.014) | 0.514\*\*\* (0.015) | 0.516\*\*\* (0.015) |
| AIC | 46418.130 | 46409.710 | 46407.150 | 46398.940 |
|  | | | | |
| † p<0.1; \* p<0.05; \*\* p<0.01; \*\*\* p<0.001; country-clustered SE's in parentheses; cubic terms for group-wise months without mobilization included but not reported. | | | | |
