# Supplementary material for: Ethnic Accommodation and the Backlash From Dominant Groups
Source: J Conflict Resolut. 2025 May 22;70(2-3):359–86. doi: 10.1177/00220027251343836 (PMC12782309; doi:10.1177/00220027251343836)
Supplement: Supplemental Material - Ethnic Accommodation and the Backlash From Dominant Groups [file sj-zip-3-jcr-10.1177_00220027251343836.zip › tables/results/app3.1_tw1.html]

**Ethnic accommodation and the number of mobilization events involving the dominant group [1-month time window].**

|  | | | | |
|  | **Model 1** | **Model 2** | **Model 3** | **Model 4** |
|  | | | | |
| Concession number | 0.116\* | 0.007 |  |  |
|  | (0.046) | (0.081) |  |  |
| Concession number x DN party |  | 0.182† |  |  |
|  |  | (0.100) |  |  |
| Concession number (group-based) |  |  | 0.185 | -0.173 |
|  |  |  | (0.136) | (0.174) |
| Concession number (group-based) x DN party |  |  |  | 0.559\* |
|  |  |  |  | (0.232) |
| Concession number (group-blind) |  |  | 0.049 | 0.165 |
|  |  |  | (0.128) | (0.202) |
| Concession number (group-blind) x DN party |  |  |  | -0.176 |
|  |  |  |  | (0.265) |
| DN party | 0.085 | 0.076 | 0.084 | 0.076 |
|  | (0.166) | (0.165) | (0.166) | (0.165) |
| DN party in government | 0.034 | 0.039 | 0.035 | 0.041 |
|  | (0.093) | (0.094) | (0.094) | (0.094) |
| Months to next election (log) | -0.063\*\* | -0.063\*\* | -0.063\*\* | -0.064\*\* |
|  | (0.023) | (0.023) | (0.023) | (0.023) |
| Recent subordinate group protest | 0.392\*\*\* | 0.392\*\*\* | 0.392\*\*\* | 0.392\*\*\* |
|  | (0.083) | (0.083) | (0.084) | (0.083) |
| Recent civil violence | 0.144 | 0.143 | 0.144 | 0.144 |
|  | (0.124) | (0.124) | (0.124) | (0.123) |
| Battle deaths (last 10y, log) | 0.063 | 0.065 | 0.064 | 0.066 |
|  | (0.072) | (0.072) | (0.072) | (0.071) |
| Democracy level | -0.413 | -0.416 | -0.409 | -0.421 |
|  | (0.332) | (0.335) | (0.334) | (0.332) |
| Abs. size (log) | 0.211 | 0.213 | 0.211 | 0.215 |
|  | (0.184) | (0.183) | (0.184) | (0.182) |
| GDP p.c. (log) | -0.229 | -0.232 | -0.227 | -0.229 |
|  | (0.300) | (0.301) | (0.300) | (0.300) |
| GDP growth | -0.967† | -0.956† | -0.972† | -0.964† |
|  | (0.504) | (0.507) | (0.506) | (0.508) |
| Regional DG mobilization events (log) | 0.066\* | 0.066\* | 0.066\* | 0.067\* |
|  | (0.029) | (0.029) | (0.029) | (0.029) |
| Constant | 0.746 | 0.781 | 0.727 | 0.747 |
|  | (3.265) | (3.267) | (3.260) | (3.259) |
| Country-FE | yes | yes | yes | yes |
| Year-FE | yes | yes | yes | yes |
| Wald-Test Chisq |  |  |  |  |
| Joint sig. int. concession |  | 0.001\*\* |  |  |
| Joint sig. int. concession (group-based) |  |  |  | 0.013\* |
| Joint sig. int. concession (group-blind) |  |  |  | 0.946 |
| N | 38130 | 38130 | 38130 | 38130 |
| Log Likelihood | -23047.620 | -23045.300 | -23047.230 | -23042.470 |
| theta | 0.511\*\*\* (0.014) | 0.512\*\*\* (0.014) | 0.511\*\*\* (0.014) | 0.512\*\*\* (0.014) |
| AIC | 46431.230 | 46428.600 | 46432.470 | 46426.950 |
|  | | | | |
| † p<0.1; \* p<0.05; \*\* p<0.01; \*\*\* p<0.001; country-clustered SE's in parentheses; cubic terms for group-wise months without mobilization included but not reported. | | | | |
