# Supplementary material for: Ethnic Accommodation and the Backlash From Dominant Groups
Source: J Conflict Resolut. 2025 May 22;70(2-3):359–86. doi: 10.1177/00220027251343836 (PMC12782309; doi:10.1177/00220027251343836)
Supplement: Supplemental Material - Ethnic Accommodation and the Backlash From Dominant Groups [file sj-zip-3-jcr-10.1177_00220027251343836.zip › tables/results/app3.1_tw2.html]

**Ethnic accommodation and the number of mobilization events involving the dominant group [2-month time window].**

|  | | | | |
|  | **Model 1** | **Model 2** | **Model 3** | **Model 4** |
|  | | | | |
| Concession number | 0.150\*\*\* | 0.090 |  |  |
|  | (0.043) | (0.063) |  |  |
| Concession number x DN party |  | 0.104 |  |  |
|  |  | (0.089) |  |  |
| Concession number (group-based) |  |  | 0.194† | -0.008 |
|  |  |  | (0.116) | (0.134) |
| Concession number (group-based) x DN party |  |  |  | 0.328 |
|  |  |  |  | (0.210) |
| Concession number (group-blind) |  |  | 0.107 | 0.180 |
|  |  |  | (0.117) | (0.142) |
| Concession number (group-blind) x DN party |  |  |  | -0.111 |
|  |  |  |  | (0.231) |
| DN party | 0.084 | 0.075 | 0.084 | 0.075 |
|  | (0.166) | (0.165) | (0.166) | (0.164) |
| DN party in government | 0.038 | 0.042 | 0.038 | 0.043 |
|  | (0.093) | (0.093) | (0.093) | (0.094) |
| Months to next election (log) | -0.060\*\* | -0.061\*\* | -0.061\*\* | -0.061\*\* |
|  | (0.022) | (0.022) | (0.022) | (0.022) |
| Recent subordinate group protest | 0.387\*\*\* | 0.388\*\*\* | 0.387\*\*\* | 0.388\*\*\* |
|  | (0.083) | (0.083) | (0.083) | (0.082) |
| Recent civil violence | 0.143 | 0.142 | 0.143 | 0.142 |
|  | (0.123) | (0.122) | (0.122) | (0.121) |
| Battle deaths (last 10y, log) | 0.064 | 0.065 | 0.064 | 0.066 |
|  | (0.072) | (0.072) | (0.072) | (0.072) |
| Democracy level | -0.405 | -0.409 | -0.401 | -0.414 |
|  | (0.327) | (0.330) | (0.331) | (0.328) |
| Abs. size (log) | 0.211 | 0.213 | 0.211 | 0.217 |
|  | (0.184) | (0.182) | (0.183) | (0.181) |
| GDP p.c. (log) | -0.225 | -0.228 | -0.223 | -0.225 |
|  | (0.300) | (0.300) | (0.299) | (0.299) |
| GDP growth | -0.947† | -0.939† | -0.953† | -0.949† |
|  | (0.504) | (0.505) | (0.505) | (0.508) |
| Regional DG mobilization events (log) | 0.067\* | 0.067\* | 0.067\* | 0.067\* |
|  | (0.029) | (0.029) | (0.029) | (0.029) |
| Constant | 0.691 | 0.717 | 0.668 | 0.687 |
|  | (3.259) | (3.263) | (3.253) | (3.251) |
| Country-FE | yes | yes | yes | yes |
| Year-FE | yes | yes | yes | yes |
| Wald-Test Chisq |  |  |  |  |
| Joint sig. int. concession |  | 0.001\*\* |  |  |
| Joint sig. int. concession (group-based) |  |  |  | 0.043\* |
| Joint sig. int. concession (group-blind) |  |  |  | 0.695 |
| N | 38130 | 38130 | 38130 | 38130 |
| Log Likelihood | -23041.110 | -23039.890 | -23040.860 | -23038.180 |
| theta | 0.512\*\*\* (0.014) | 0.513\*\*\* (0.014) | 0.513\*\*\* (0.014) | 0.513\*\*\* (0.014) |
| AIC | 46418.230 | 46417.780 | 46419.730 | 46418.360 |
|  | | | | |
| † p<0.1; \* p<0.05; \*\* p<0.01; \*\*\* p<0.001; country-clustered SE's in parentheses; cubic terms for group-wise months without mobilization included but not reported. | | | | |
