# Supplementary material for: Ethnic Accommodation and the Backlash From Dominant Groups
Source: J Conflict Resolut. 2025 May 22;70(2-3):359–86. doi: 10.1177/00220027251343836 (PMC12782309; doi:10.1177/00220027251343836)
Supplement: Supplemental Material - Ethnic Accommodation and the Backlash From Dominant Groups [file sj-zip-3-jcr-10.1177_00220027251343836.zip › tables/results/app3.1_tw6.html]

**Ethnic accommodation and the number of mobilization events involving the dominant group [6-month time window].**

|  | | | | |
|  | **Model 1** | **Model 2** | **Model 3** | **Model 4** |
|  | | | | |
| Concession number | 0.095\*\* | 0.022 |  |  |
|  | (0.034) | (0.050) |  |  |
| Concession number x DN party |  | 0.125† |  |  |
|  |  | (0.066) |  |  |
| Concession number (group-based) |  |  | 0.216\* | 0.028 |
|  |  |  | (0.085) | (0.093) |
| Concession number (group-based) x DN party |  |  |  | 0.305\* |
|  |  |  |  | (0.140) |
| Concession number (group-blind) |  |  | -0.030 | 0.015 |
|  |  |  | (0.081) | (0.105) |
| Concession number (group-blind) x DN party |  |  |  | -0.063 |
|  |  |  |  | (0.146) |
| DN party | 0.083 | 0.054 | 0.085 | 0.060 |
|  | (0.166) | (0.166) | (0.165) | (0.165) |
| DN party in government | 0.041 | 0.053 | 0.043 | 0.055 |
|  | (0.093) | (0.093) | (0.094) | (0.095) |
| Months to next election (log) | -0.061\*\* | -0.062\*\* | -0.063\*\* | -0.064\*\* |
|  | (0.023) | (0.023) | (0.023) | (0.023) |
| Recent subordinate group protest | 0.393\*\*\* | 0.395\*\*\* | 0.394\*\*\* | 0.397\*\*\* |
|  | (0.083) | (0.083) | (0.083) | (0.083) |
| Recent civil violence | 0.149 | 0.147 | 0.145 | 0.142 |
|  | (0.123) | (0.120) | (0.121) | (0.118) |
| Battle deaths (last 10y, log) | 0.066 | 0.069 | 0.067 | 0.071 |
|  | (0.072) | (0.071) | (0.071) | (0.070) |
| Democracy level | -0.406 | -0.409 | -0.374 | -0.401 |
|  | (0.324) | (0.330) | (0.333) | (0.328) |
| Abs. size (log) | 0.211 | 0.216 | 0.213 | 0.227 |
|  | (0.182) | (0.179) | (0.180) | (0.176) |
| GDP p.c. (log) | -0.223 | -0.227 | -0.210 | -0.213 |
|  | (0.299) | (0.300) | (0.296) | (0.296) |
| GDP growth | -0.934† | -0.918† | -0.959† | -0.961† |
|  | (0.501) | (0.501) | (0.509) | (0.509) |
| Regional DG mobilization events (log) | 0.067\* | 0.067\* | 0.067\* | 0.067\* |
|  | (0.029) | (0.029) | (0.029) | (0.029) |
| Constant | 0.666 | 0.705 | 0.517 | 0.534 |
|  | (3.250) | (3.252) | (3.220) | (3.209) |
| Country-FE | yes | yes | yes | yes |
| Year-FE | yes | yes | yes | yes |
| Wald-Test Chisq |  |  |  |  |
| Joint sig. int. concession |  | 0.001\*\* |  |  |
| Joint sig. int. concession (group-based) |  |  |  | 0.002\*\* |
| Joint sig. int. concession (group-blind) |  |  |  | 0.648 |
| N | 38130 | 38130 | 38130 | 38130 |
| Log Likelihood | -23042.400 | -23038.400 | -23037.730 | -23031.620 |
| theta | 0.511\*\*\* (0.014) | 0.512\*\*\* (0.014) | 0.513\*\*\* (0.014) | 0.514\*\*\* (0.014) |
| AIC | 46420.800 | 46414.800 | 46413.450 | 46405.240 |
|  | | | | |
| † p<0.1; \* p<0.05; \*\* p<0.01; \*\*\* p<0.001; country-clustered SE's in parentheses; cubic terms for group-wise months without mobilization included but not reported. | | | | |
