# Supplementary material for: Ethnic Accommodation and the Backlash From Dominant Groups
Source: J Conflict Resolut. 2025 May 22;70(2-3):359–86. doi: 10.1177/00220027251343836 (PMC12782309; doi:10.1177/00220027251343836)
Supplement: Supplemental Material - Ethnic Accommodation and the Backlash From Dominant Groups [file sj-zip-3-jcr-10.1177_00220027251343836.zip › tables/results/app3.2_major.html]

**Ethnic accommodation and the number of mobilization events involving the dominant group [major mobilization incidents].**

|  | | | | |
|  | **Model 1** | **Model 2** | **Model 3** | **Model 4** |
|  | | | | |
| Concession number | 0.089\* | 0.067 |  |  |
|  | (0.036) | (0.059) |  |  |
| Concession number x DN party |  | 0.037 |  |  |
|  |  | (0.070) |  |  |
| Concession number (group-based) |  |  | 0.104 | -0.025 |
|  |  |  | (0.101) | (0.124) |
| Concession number (group-based) x DN party |  |  |  | 0.220 |
|  |  |  |  | (0.184) |
| Concession number (group-blind) |  |  | 0.073 | 0.154 |
|  |  |  | (0.104) | (0.139) |
| Concession number (group-blind) x DN party |  |  |  | -0.137 |
|  |  |  |  | (0.191) |
| DN party | 0.011 | 0.007 | 0.012 | 0.008 |
|  | (0.149) | (0.149) | (0.149) | (0.148) |
| DN party in government | 0.019 | 0.022 | 0.020 | 0.023 |
|  | (0.103) | (0.103) | (0.103) | (0.104) |
| Months to next election (log) | -0.052\* | -0.052\* | -0.052\* | -0.053\* |
|  | (0.023) | (0.023) | (0.023) | (0.023) |
| Recent subordinate group protest | 0.365\*\*\* | 0.365\*\*\* | 0.365\*\*\* | 0.366\*\*\* |
|  | (0.085) | (0.085) | (0.085) | (0.085) |
| Recent civil violence | 0.141 | 0.141 | 0.141 | 0.140 |
|  | (0.114) | (0.113) | (0.113) | (0.112) |
| Battle deaths (last 10y, log) | 0.035 | 0.035 | 0.035 | 0.036 |
|  | (0.069) | (0.069) | (0.069) | (0.069) |
| Democracy level | -0.727\* | -0.729\* | -0.725\* | -0.738\*\* |
|  | (0.282) | (0.283) | (0.285) | (0.284) |
| Abs. size (log) | 0.092 | 0.093 | 0.093 | 0.098 |
|  | (0.149) | (0.149) | (0.149) | (0.148) |
| GDP p.c. (log) | -0.296 | -0.297 | -0.295 | -0.295 |
|  | (0.267) | (0.268) | (0.267) | (0.267) |
| GDP growth | -0.705 | -0.704 | -0.706 | -0.711 |
|  | (0.506) | (0.507) | (0.507) | (0.510) |
| Regional DG mobilization events (log) | 0.052† | 0.052† | 0.052† | 0.052† |
|  | (0.030) | (0.030) | (0.030) | (0.030) |
| Constant | 1.708 | 1.721 | 1.698 | 1.690 |
|  | (2.878) | (2.882) | (2.873) | (2.872) |
| Country-FE | yes | yes | yes | yes |
| Year-FE | yes | yes | yes | yes |
| Wald-Test Chisq |  |  |  |  |
| Joint sig. int. concession |  | 0.015\* |  |  |
| Joint sig. int. concession (group-based) |  |  |  | 0.152 |
| Joint sig. int. concession (group-blind) |  |  |  | 0.906 |
| N | 38130 | 38130 | 38130 | 38130 |
| Log Likelihood | -18513.650 | -18513.480 | -18513.620 | -18512.370 |
| theta | 0.484\*\*\* (0.017) | 0.484\*\*\* (0.017) | 0.484\*\*\* (0.017) | 0.484\*\*\* (0.017) |
| AIC | 37363.310 | 37364.960 | 37365.240 | 37366.740 |
|  | | | | |
| † p<0.1; \* p<0.05; \*\* p<0.01; \*\*\* p<0.001; country-clustered SE's in parentheses; cubic terms for group-wise months without mobilization included but not reported. | | | | |
