# Supplementary material for: Ethnic Accommodation and the Backlash From Dominant Groups
Source: J Conflict Resolut. 2025 May 22;70(2-3):359–86. doi: 10.1177/00220027251343836 (PMC12782309; doi:10.1177/00220027251343836)
Supplement: Supplemental Material - Ethnic Accommodation and the Backlash From Dominant Groups [file sj-zip-3-jcr-10.1177_00220027251343836.zip › tables/results/app3.2_non_violent.html]

**Ethnic accommodation and the number of mobilization events involving the dominant group [non-violent mobilization events].**

|  | | | | |
|  | **Model 1** | **Model 2** | **Model 3** | **Model 4** |
|  | | | | |
| Concession number | 0.060 | 0.032 |  |  |
|  | (0.050) | (0.093) |  |  |
| Concession number x DN party |  | 0.048 |  |  |
|  |  | (0.100) |  |  |
| Concession number (group-based) |  |  | 0.128 | 0.076 |
|  |  |  | (0.097) | (0.121) |
| Concession number (group-based) x DN party |  |  |  | 0.087 |
|  |  |  |  | (0.168) |
| Concession number (group-blind) |  |  | -0.006 | -0.011 |
|  |  |  | (0.109) | (0.146) |
| Concession number (group-blind) x DN party |  |  |  | 0.009 |
|  |  |  |  | (0.215) |
| DN party | 0.140 | 0.134 | 0.140 | 0.135 |
|  | (0.167) | (0.167) | (0.167) | (0.166) |
| DN party in government | 0.050 | 0.052 | 0.050 | 0.053 |
|  | (0.101) | (0.101) | (0.102) | (0.101) |
| Months to next election (log) | -0.039† | -0.039† | -0.039† | -0.040† |
|  | (0.023) | (0.023) | (0.023) | (0.023) |
| Recent subordinate group protest | 0.373\*\*\* | 0.372\*\*\* | 0.372\*\*\* | 0.372\*\*\* |
|  | (0.100) | (0.099) | (0.099) | (0.099) |
| Recent civil violence | 0.022 | 0.022 | 0.021 | 0.022 |
|  | (0.119) | (0.119) | (0.119) | (0.119) |
| Battle deaths (last 10y, log) | 0.074 | 0.075 | 0.075 | 0.076 |
|  | (0.077) | (0.077) | (0.076) | (0.077) |
| Democracy level | -0.820\* | -0.820\* | -0.810\* | -0.812\* |
|  | (0.347) | (0.349) | (0.349) | (0.350) |
| Abs. size (log) | 0.607\* | 0.607\* | 0.608\* | 0.609\* |
|  | (0.304) | (0.303) | (0.302) | (0.302) |
| GDP p.c. (log) | 0.149 | 0.148 | 0.152 | 0.151 |
|  | (0.194) | (0.194) | (0.194) | (0.193) |
| GDP growth | -1.015\* | -1.013\* | -1.020\* | -1.019\* |
|  | (0.472) | (0.473) | (0.474) | (0.475) |
| Regional DG mobilization events (log) | 0.058† | 0.058† | 0.058† | 0.058† |
|  | (0.033) | (0.033) | (0.033) | (0.033) |
| Constant | -4.595† | -4.587† | -4.627† | -4.619† |
|  | (2.402) | (2.397) | (2.397) | (2.392) |
| Country-FE | yes | yes | yes | yes |
| Year-FE | yes | yes | yes | yes |
| Wald-Test Chisq |  |  |  |  |
| Joint sig. int. concession |  | 0.127 |  |  |
| Joint sig. int. concession (group-based) |  |  |  | 0.221 |
| Joint sig. int. concession (group-blind) |  |  |  | 0.99 |
| N | 38130 | 38130 | 38130 | 38130 |
| Log Likelihood | -17786.370 | -17786.130 | -17785.820 | -17785.540 |
| theta | 0.528\*\*\* (0.019) | 0.528\*\*\* (0.019) | 0.528\*\*\* (0.019) | 0.528\*\*\* (0.019) |
| AIC | 35908.740 | 35910.250 | 35909.650 | 35913.070 |
|  | | | | |
| † p<0.1; \* p<0.05; \*\* p<0.01; \*\*\* p<0.001; country-clustered SE's in parentheses; cubic terms for group-wise months without mobilization included but not reported. | | | | |
