# Supplementary material for: Ethnic Accommodation and the Backlash From Dominant Groups
Source: J Conflict Resolut. 2025 May 22;70(2-3):359–86. doi: 10.1177/00220027251343836 (PMC12782309; doi:10.1177/00220027251343836)
Supplement: Supplemental Material - Ethnic Accommodation and the Backlash From Dominant Groups [file sj-zip-3-jcr-10.1177_00220027251343836.zip › tables/results/app3.2_violent.html]

**Ethnic accommodation and the number of mobilization events involving the dominant group [violent mobilization events].**

|  | | | | |
|  | **Model 1** | **Model 2** | **Model 3** | **Model 4** |
|  | | | | |
| Concession number | 0.307\*\*\* | 0.185\* |  |  |
|  | (0.056) | (0.072) |  |  |
| Concession number x DN party |  | 0.211† |  |  |
|  |  | (0.113) |  |  |
| Concession number (group-based) |  |  | 0.454\* | 0.037 |
|  |  |  | (0.177) | (0.212) |
| Concession number (group-based) x DN party |  |  |  | 0.656\* |
|  |  |  |  | (0.321) |
| Concession number (group-blind) |  |  | 0.153 | 0.316 |
|  |  |  | (0.178) | (0.226) |
| Concession number (group-blind) x DN party |  |  |  | -0.247 |
|  |  |  |  | (0.332) |
| DN party | 0.123 | 0.091 | 0.114 | 0.080 |
|  | (0.266) | (0.263) | (0.264) | (0.258) |
| DN party in government | 0.101 | 0.114 | 0.106 | 0.125 |
|  | (0.149) | (0.148) | (0.149) | (0.150) |
| Months to next election (log) | -0.089\* | -0.090\* | -0.092\* | -0.095\*\* |
|  | (0.036) | (0.035) | (0.036) | (0.035) |
| Recent subordinate group protest | 0.528\*\*\* | 0.533\*\*\* | 0.528\*\*\* | 0.534\*\*\* |
|  | (0.117) | (0.118) | (0.116) | (0.117) |
| Recent civil violence | 0.415\* | 0.409\* | 0.413\* | 0.408\* |
|  | (0.187) | (0.184) | (0.184) | (0.181) |
| Battle deaths (last 10y, log) | 0.104 | 0.106 | 0.105 | 0.112 |
|  | (0.101) | (0.100) | (0.100) | (0.099) |
| Democracy level | 0.386 | 0.378 | 0.412 | 0.370 |
|  | (0.497) | (0.500) | (0.505) | (0.494) |
| Abs. size (log) | -0.170 | -0.164 | -0.172 | -0.153 |
|  | (0.311) | (0.308) | (0.312) | (0.307) |
| GDP p.c. (log) | -0.434 | -0.440 | -0.416 | -0.418 |
|  | (0.581) | (0.583) | (0.579) | (0.580) |
| GDP growth | -2.031\*\* | -1.992\*\* | -2.084\*\* | -2.061\*\* |
|  | (0.752) | (0.754) | (0.754) | (0.757) |
| Regional DG mobilization events (log) | 1.837 | 1.902 | 1.653 | 1.662 |
|  | (6.199) | (6.213) | (6.183) | (6.190) |
| Country-FE | yes | yes | yes | yes |
| Year-FE | yes | yes | yes | yes |
| Wald-Test Chisq |  |  |  |  |
| Joint sig. int. concession |  | 0\*\*\* |  |  |
| Joint sig. int. concession (group-based) |  |  |  | 0.003\*\* |
| Joint sig. int. concession (group-blind) |  |  |  | 0.779 |
| N | 38215 | 38215 | 38215 | 38215 |
| Log Likelihood | -10436.590 | -10433.680 | -10434.820 | -10428.570 |
| theta | 0.249\*\*\* (0.011) | 0.250\*\*\* (0.011) | 0.249\*\*\* (0.011) | 0.252\*\*\* (0.011) |
| AIC | 21201.180 | 21197.360 | 21199.630 | 21191.130 |
|  | | | | |
| † p<0.1; \* p<0.05; \*\* p<0.01; \*\*\* p<0.001; country-clustered SE's in parentheses; cubic terms for group-wise months without mobilization included but not reported. | | | | |
