# Supplementary material for: Ethnic Accommodation and the Backlash From Dominant Groups
Source: J Conflict Resolut. 2025 May 22;70(2-3):359–86. doi: 10.1177/00220027251343836 (PMC12782309; doi:10.1177/00220027251343836)
Supplement: Supplemental Material - Ethnic Accommodation and the Backlash From Dominant Groups [file sj-zip-3-jcr-10.1177_00220027251343836.zip › tables/results/app3.3_democracies.html]

**Ethnic accommodation and the number of mobilization events involving the dominant group [only electoral or liberal democracies].**

|  | | | | |
|  | **Model 1** | **Model 2** | **Model 3** | **Model 4** |
|  | | | | |
| Concession number | 0.177\*\* | 0.103 |  |  |
|  | (0.060) | (0.105) |  |  |
| Concession number x DN party |  | 0.111 |  |  |
|  |  | (0.119) |  |  |
| Concession number (group-based) |  |  | 0.364\*\*\* | 0.250 |
|  |  |  | (0.105) | (0.214) |
| Concession number (group-based) x DN party |  |  |  | 0.158 |
|  |  |  |  | (0.230) |
| Concession number (group-blind) |  |  | -0.024 | -0.018 |
|  |  |  | (0.087) | (0.161) |
| Concession number (group-blind) x DN party |  |  |  | -0.004 |
|  |  |  |  | (0.193) |
| DN party | 0.169 | 0.161 | 0.170 | 0.165 |
|  | (0.220) | (0.220) | (0.220) | (0.221) |
| DN party in government | 0.035 | 0.036 | 0.035 | 0.036 |
|  | (0.137) | (0.137) | (0.136) | (0.136) |
| Months to next election (log) | 0.027 | 0.026 | 0.025 | 0.025 |
|  | (0.030) | (0.030) | (0.030) | (0.030) |
| Recent subordinate group protest | 0.300\*\*\* | 0.304\*\*\* | 0.299\*\*\* | 0.301\*\*\* |
|  | (0.084) | (0.084) | (0.083) | (0.083) |
| Recent civil violence | 0.318 | 0.314 | 0.303 | 0.301 |
|  | (0.211) | (0.209) | (0.204) | (0.202) |
| Battle deaths (last 10y, log) | 0.091 | 0.092 | 0.099 | 0.100 |
|  | (0.090) | (0.090) | (0.089) | (0.088) |
| Democracy level | -0.614 | -0.620 | -0.561 | -0.569 |
|  | (0.628) | (0.625) | (0.625) | (0.621) |
| Abs. size (log) | 0.352 | 0.354 | 0.365 | 0.369 |
|  | (0.328) | (0.328) | (0.325) | (0.325) |
| GDP p.c. (log) | -0.375 | -0.371 | -0.367 | -0.363 |
|  | (0.308) | (0.307) | (0.304) | (0.304) |
| GDP growth | -0.119 | -0.118 | -0.129 | -0.136 |
|  | (0.817) | (0.819) | (0.814) | (0.816) |
| Regional DG mobilization events (log) | 0.048 | 0.048 | 0.049 | 0.049 |
|  | (0.032) | (0.032) | (0.032) | (0.032) |
| Constant | 1.922 | 1.895 | 1.777 | 1.737 |
|  | (3.289) | (3.282) | (3.239) | (3.232) |
| Country-FE | yes | yes | yes | yes |
| Year-FE | yes | yes | yes | yes |
| Wald-Test Chisq |  |  |  |  |
| Joint sig. int. concession |  | 0.001\*\* |  |  |
| Joint sig. int. concession (group-based) |  |  |  | 0\*\*\* |
| Joint sig. int. concession (group-blind) |  |  |  | 0.827 |
| N | 20289 | 20289 | 20289 | 20289 |
| Log Likelihood | -12328.390 | -12327.870 | -12325.110 | -12324.750 |
| theta | 0.694\*\*\* (0.029) | 0.695\*\*\* (0.029) | 0.696\*\*\* (0.029) | 0.697\*\*\* (0.029) |
| AIC | 24910.780 | 24911.730 | 24906.220 | 24909.500 |
|  | | | | |
| † p<0.1; \* p<0.05; \*\* p<0.01; \*\*\* p<0.001; country-clustered SE's in parentheses; cubic terms for group-wise months without mobilization included but not reported. | | | | |
