# Supplementary material for: Ethnic Accommodation and the Backlash From Dominant Groups
Source: J Conflict Resolut. 2025 May 22;70(2-3):359–86. doi: 10.1177/00220027251343836 (PMC12782309; doi:10.1177/00220027251343836)
Supplement: Supplemental Material - Ethnic Accommodation and the Backlash From Dominant Groups [file sj-zip-3-jcr-10.1177_00220027251343836.zip › tables/results/app3.3_plurality.html]

**Ethnic accommodation and the number of mobilization events involving the dominant group [only demographic plurality groups].**

|  | | | | |
|  | **Model 1** | **Model 2** | **Model 3** | **Model 4** |
|  | | | | |
| Concession number | 0.212\*\*\* | 0.149† |  |  |
|  | (0.052) | (0.076) |  |  |
| Concession number x DN party |  | 0.115 |  |  |
|  |  | (0.107) |  |  |
| Concession number (group-based) |  |  | 0.313\*\* | 0.164 |
|  |  |  | (0.119) | (0.144) |
| Concession number (group-based) x DN party |  |  |  | 0.253 |
|  |  |  |  | (0.217) |
| Concession number (group-blind) |  |  | 0.111 | 0.134 |
|  |  |  | (0.136) | (0.153) |
| Concession number (group-blind) x DN party |  |  |  | -0.027 |
|  |  |  |  | (0.253) |
| DN party | 0.039 | 0.023 | 0.037 | 0.021 |
|  | (0.233) | (0.232) | (0.232) | (0.231) |
| DN party in government | 0.143 | 0.150 | 0.145 | 0.152 |
|  | (0.116) | (0.116) | (0.116) | (0.117) |
| Months to next election (log) | -0.052\* | -0.053\* | -0.054\* | -0.055\* |
|  | (0.026) | (0.026) | (0.026) | (0.026) |
| Recent subordinate group protest | 0.565\*\*\* | 0.566\*\*\* | 0.565\*\*\* | 0.568\*\*\* |
|  | (0.097) | (0.097) | (0.097) | (0.097) |
| Recent civil violence | 0.232 | 0.228 | 0.231 | 0.227 |
|  | (0.161) | (0.159) | (0.160) | (0.159) |
| Battle deaths (last 10y, log) | 0.167† | 0.168† | 0.168† | 0.171\* |
|  | (0.087) | (0.086) | (0.086) | (0.086) |
| Democracy level | -0.896† | -0.898† | -0.879† | -0.893† |
|  | (0.522) | (0.526) | (0.526) | (0.528) |
| Abs. size (log) | 1.707\* | 1.708\* | 1.714\* | 1.701\* |
|  | (0.700) | (0.704) | (0.714) | (0.711) |
| GDP p.c. (log) | -0.193 | -0.195 | -0.186 | -0.188 |
|  | (0.403) | (0.405) | (0.402) | (0.404) |
| GDP growth | -0.742 | -0.726 | -0.761 | -0.751 |
|  | (0.511) | (0.511) | (0.516) | (0.517) |
| Regional DG mobilization events (log) | -4.140 | -4.113 | -4.231 | -4.156 |
|  | (5.307) | (5.346) | (5.328) | (5.356) |
| Country-FE | yes | yes | yes | yes |
| Year-FE | yes | yes | yes | yes |
| Wald-Test Chisq |  |  |  |  |
| Joint sig. int. concession |  | 0\*\*\* |  |  |
| Joint sig. int. concession (group-based) |  |  |  | 0.012\* |
| Joint sig. int. concession (group-blind) |  |  |  | 0.605 |
| N | 35188 | 35188 | 35188 | 35188 |
| Log Likelihood | -22451.580 | -22449.770 | -22449.850 | -22447.410 |
| theta | 0.435\*\*\* (0.012) | 0.435\*\*\* (0.012) | 0.435\*\*\* (0.012) | 0.436\*\*\* (0.012) |
| AIC | 45211.160 | 45209.540 | 45209.710 | 45208.830 |
|  | | | | |
| † p<0.1; \* p<0.05; \*\* p<0.01; \*\*\* p<0.001; country-clustered SE's in parentheses; cubic terms for group-wise months without mobilization included but not reported. | | | | |
