# Supplementary material for: Ethnic Accommodation and the Backlash From Dominant Groups
Source: J Conflict Resolut. 2025 May 22;70(2-3):359–86. doi: 10.1177/00220027251343836 (PMC12782309; doi:10.1177/00220027251343836)
Supplement: Supplemental Material - Ethnic Accommodation and the Backlash From Dominant Groups [file sj-zip-3-jcr-10.1177_00220027251343836.zip › figures/margins/app3.3_plurality.pdf]

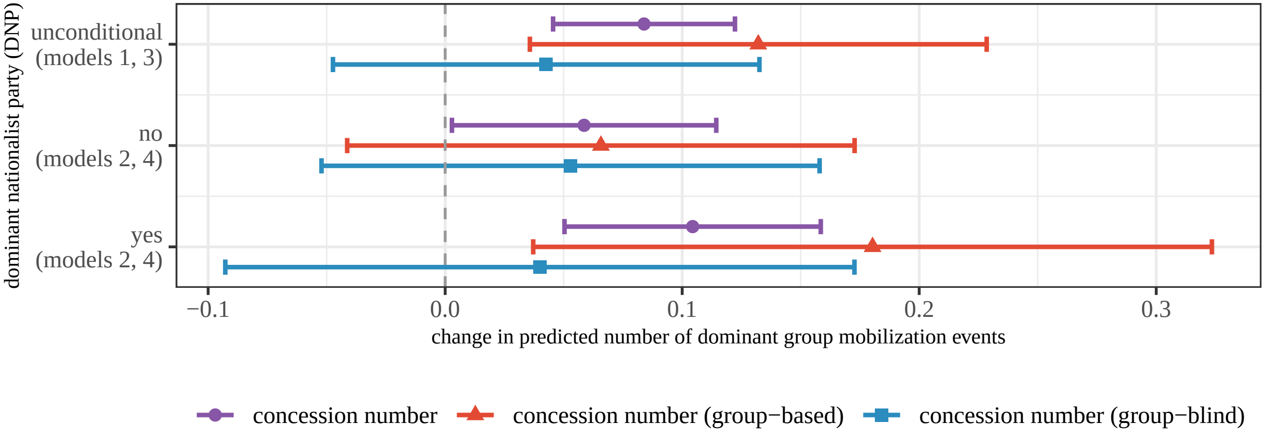

Wald test for difference in AME between DNP = 1 vs. DNP = 0:
concession number: 0.339; concession number (group−based): 0.287; concession number (group−blind): 0.898
