# Supplementary material for: Ethnic Accommodation and the Backlash From Dominant Groups
Source: J Conflict Resolut. 2025 May 22;70(2-3):359–86. doi: 10.1177/00220027251343836 (PMC12782309; doi:10.1177/00220027251343836)
Supplement: Supplemental Material - Ethnic Accommodation and the Backlash From Dominant Groups [file sj-zip-3-jcr-10.1177_00220027251343836.zip › tables/results/app3.3_sc_last12.html]

**Ethnic accommodation and the number of mobilization events involving the dominant group [including groups that lost dominance in the last 12 months].**

|  | | | | |
|  | **Model 1** | **Model 2** | **Model 3** | **Model 4** |
|  | | | | |
| Concession number | 0.167\*\*\* | 0.109 |  |  |
|  | (0.043) | (0.067) |  |  |
| Concession number x DN party |  | 0.100 |  |  |
|  |  | (0.092) |  |  |
| Concession number (group-based) |  |  | 0.297\*\* | 0.134 |
|  |  |  | (0.112) | (0.136) |
| Concession number (group-based) x DN party |  |  |  | 0.274 |
|  |  |  |  | (0.211) |
| Concession number (group-blind) |  |  | 0.040 | 0.085 |
|  |  |  | (0.122) | (0.157) |
| Concession number (group-blind) x DN party |  |  |  | -0.073 |
|  |  |  |  | (0.238) |
| DN party | 0.113 | 0.099 | 0.111 | 0.098 |
|  | (0.196) | (0.195) | (0.195) | (0.193) |
| DN party in government | 0.098 | 0.104 | 0.101 | 0.108 |
|  | (0.114) | (0.114) | (0.115) | (0.115) |
| Months to next election (log) | -0.059\* | -0.059\* | -0.061\* | -0.061\* |
|  | (0.025) | (0.025) | (0.025) | (0.025) |
| Recent subordinate group protest | 0.489\*\*\* | 0.490\*\*\* | 0.489\*\*\* | 0.491\*\*\* |
|  | (0.089) | (0.089) | (0.089) | (0.089) |
| Recent civil violence | 0.220 | 0.218 | 0.220 | 0.218 |
|  | (0.146) | (0.145) | (0.145) | (0.144) |
| Battle deaths (last 10y, log) | 0.068 | 0.070 | 0.069 | 0.072 |
|  | (0.079) | (0.078) | (0.078) | (0.078) |
| Democracy level | -0.555 | -0.561 | -0.537 | -0.552 |
|  | (0.418) | (0.421) | (0.423) | (0.423) |
| Abs. size (log) | 0.226 | 0.231 | 0.226 | 0.233 |
|  | (0.209) | (0.209) | (0.208) | (0.208) |
| GDP p.c. (log) | -0.281 | -0.283 | -0.272 | -0.273 |
|  | (0.347) | (0.347) | (0.345) | (0.345) |
| GDP growth | -1.081† | -1.075† | -1.108† | -1.106† |
|  | (0.568) | (0.568) | (0.572) | (0.573) |
| Regional DG mobilization events (log) | 0.738 | 0.755 | 0.648 | 0.646 |
|  | (3.721) | (3.724) | (3.705) | (3.701) |
| Country-FE | yes | yes | yes | yes |
| Year-FE | yes | yes | yes | yes |
| Wald-Test Chisq |  |  |  |  |
| Joint sig. int. concession |  | 0\*\*\* |  |  |
| Joint sig. int. concession (group-based) |  |  |  | 0.011\* |
| Joint sig. int. concession (group-blind) |  |  |  | 0.949 |
| N | 38681 | 38681 | 38681 | 38681 |
| Log Likelihood | -24019.360 | -24017.810 | -24016.430 | -24013.720 |
| theta | 0.423\*\*\* (0.011) | 0.424\*\*\* (0.011) | 0.424\*\*\* (0.011) | 0.425\*\*\* (0.011) |
| AIC | 48366.720 | 48365.630 | 48362.870 | 48361.430 |
|  | | | | |
| † p<0.1; \* p<0.05; \*\* p<0.01; \*\*\* p<0.001; country-clustered SE's in parentheses; cubic terms for group-wise months without mobilization included but not reported. | | | | |
