# Supplementary material for: Ethnic Accommodation and the Backlash From Dominant Groups
Source: J Conflict Resolut. 2025 May 22;70(2-3):359–86. doi: 10.1177/00220027251343836 (PMC12782309; doi:10.1177/00220027251343836)
Supplement: Supplemental Material - Ethnic Accommodation and the Backlash From Dominant Groups [file sj-zip-3-jcr-10.1177_00220027251343836.zip › tables/results/app3.4_ac1.html]

**Ethnic accommodation and the number of mobilization events involving the dominant group [additional structural controls].**

|  | | | | |
|  | **Model 1** | **Model 2** | **Model 3** | **Model 4** |
|  | | | | |
| Concession number | 0.144\*\*\* | 0.072 |  |  |
|  | (0.041) | (0.073) |  |  |
| Concession number x DN party |  | 0.119 |  |  |
|  |  | (0.090) |  |  |
| Concession number (group-based) |  |  | 0.302\*\* | 0.190 |
|  |  |  | (0.106) | (0.116) |
| Concession number (group-based) x DN party |  |  |  | 0.179 |
|  |  |  |  | (0.178) |
| Concession number (group-blind) |  |  | -0.011 | -0.038 |
|  |  |  | (0.110) | (0.129) |
| Concession number (group-blind) x DN party |  |  |  | 0.050 |
|  |  |  |  | (0.200) |
| DN party | 0.180 | 0.166 | 0.176 | 0.163 |
|  | (0.165) | (0.163) | (0.163) | (0.161) |
| DN party in government | 0.058 | 0.065 | 0.061 | 0.068 |
|  | (0.096) | (0.097) | (0.098) | (0.098) |
| Months to next election (log) | -0.063\*\* | -0.063\*\* | -0.065\*\* | -0.065\*\* |
|  | (0.024) | (0.024) | (0.024) | (0.024) |
| Recent subordinate group protest | 0.362\*\*\* | 0.364\*\*\* | 0.362\*\*\* | 0.363\*\*\* |
|  | (0.084) | (0.084) | (0.084) | (0.083) |
| Recent civil violence | 0.166 | 0.163 | 0.164 | 0.162 |
|  | (0.125) | (0.124) | (0.124) | (0.122) |
| Battle deaths (last 10y, log) | 0.067 | 0.068 | 0.068 | 0.070 |
|  | (0.077) | (0.077) | (0.077) | (0.076) |
| Democracy level | -0.245 | -0.251 | -0.226 | -0.236 |
|  | (0.323) | (0.326) | (0.327) | (0.328) |
| Abs. size (log) | 0.242 | 0.241 | 0.238 | 0.238 |
|  | (0.222) | (0.220) | (0.223) | (0.221) |
| GDP p.c. (log) | -0.059 | -0.061 | -0.051 | -0.053 |
|  | (0.202) | (0.201) | (0.200) | (0.199) |
| GDP growth | -1.531\*\* | -1.522\*\* | -1.566\*\* | -1.558\*\* |
|  | (0.485) | (0.481) | (0.495) | (0.491) |
| Regional DG mobilization events (log) | 0.092 | 0.093 | 0.092 | 0.093 |
|  | (0.095) | (0.095) | (0.094) | (0.093) |
| tt\_vdem2\_dem | 0.045 | 0.043 | 0.043 | 0.041 |
|  | (0.079) | (0.079) | (0.078) | (0.078) |
| unemployment\_mod | 0.793 | 0.790 | 0.827 | 0.823 |
|  | (0.898) | (0.903) | (0.896) | (0.900) |
| gini | -0.015 | -0.015 | -0.014 | -0.014 |
|  | (0.009) | (0.009) | (0.009) | (0.009) |
| lunreg\_backlash\_no | 0.064\* | 0.064\* | 0.064\* | 0.064\* |
|  | (0.030) | (0.030) | (0.030) | (0.030) |
| Constant | -0.797 | -0.761 | -0.877 | -0.851 |
|  | (2.568) | (2.558) | (2.546) | (2.533) |
| Country-FE | yes | yes | yes | yes |
| Year-FE | yes | yes | yes | yes |
| Wald-Test Chisq |  |  |  |  |
| Joint sig. int. concession |  | 0\*\*\* |  |  |
| Joint sig. int. concession (group-based) |  |  |  | 0.012\* |
| Joint sig. int. concession (group-blind) |  |  |  | 0.939 |
| N | 35335 | 35335 | 35335 | 35335 |
| Log Likelihood | -21606.270 | -21604.310 | -21602.060 | -21600.140 |
| theta | 0.534\*\*\* (0.016) | 0.534\*\*\* (0.016) | 0.535\*\*\* (0.016) | 0.535\*\*\* (0.016) |
| AIC | 43532.530 | 43530.620 | 43526.120 | 43526.270 |
|  | | | | |
| † p<0.1; \* p<0.05; \*\* p<0.01; \*\*\* p<0.001; country-clustered SE's in parentheses; cubic terms for group-wise months without mobilization included but not reported. | | | | |
