# Supplementary material for: Ethnic Accommodation and the Backlash From Dominant Groups
Source: J Conflict Resolut. 2025 May 22;70(2-3):359–86. doi: 10.1177/00220027251343836 (PMC12782309; doi:10.1177/00220027251343836)
Supplement: Supplemental Material - Ethnic Accommodation and the Backlash From Dominant Groups [file sj-zip-3-jcr-10.1177_00220027251343836.zip › tables/results/app3.4_ac2.html]

**Ethnic accommodation and the number of mobilization events involving the dominant group [additional bargaining controls].**

|  | | | | |
|  | **Model 1** | **Model 2** | **Model 3** | **Model 4** |
|  | | | | |
| Concession number | 0.150\*\*\* | 0.101† |  |  |
|  | (0.039) | (0.057) |  |  |
| Concession number x DN party |  | 0.084 |  |  |
|  |  | (0.078) |  |  |
| Concession number (group-based) |  |  | 0.253\* | 0.050 |
|  |  |  | (0.106) | (0.120) |
| Concession number (group-based) x DN party |  |  |  | 0.332† |
|  |  |  |  | (0.191) |
| Concession number (group-blind) |  |  | 0.048 | 0.148 |
|  |  |  | (0.107) | (0.123) |
| Concession number (group-blind) x DN party |  |  |  | -0.162 |
|  |  |  |  | (0.199) |
| DN party | 0.082 | 0.071 | 0.081 | 0.071 |
|  | (0.167) | (0.166) | (0.166) | (0.165) |
| DN party in government | 0.034 | 0.039 | 0.036 | 0.042 |
|  | (0.092) | (0.092) | (0.093) | (0.094) |
| Months to next election (log) | -0.059\* | -0.060\*\* | -0.061\*\* | -0.061\*\* |
|  | (0.023) | (0.023) | (0.023) | (0.023) |
| Recent subordinate group protest | 0.368\*\*\* | 0.369\*\*\* | 0.368\*\*\* | 0.370\*\*\* |
|  | (0.077) | (0.077) | (0.077) | (0.077) |
| Recent civil violence | 0.139 | 0.138 | 0.138 | 0.137 |
|  | (0.119) | (0.118) | (0.118) | (0.116) |
| Battle deaths (last 10y, log) | 0.061 | 0.062 | 0.061 | 0.064 |
|  | (0.066) | (0.066) | (0.066) | (0.066) |
| Democracy level | -0.409 | -0.416 | -0.390 | -0.414 |
|  | (0.350) | (0.354) | (0.355) | (0.353) |
| Abs. size (log) | 0.354 | 0.347 | 0.352 | 0.352 |
|  | (0.369) | (0.370) | (0.369) | (0.368) |
| GDP p.c. (log) | -0.241 | -0.243 | -0.234 | -0.235 |
|  | (0.300) | (0.301) | (0.298) | (0.299) |
| GDP growth | -0.944† | -0.937† | -0.962† | -0.962† |
|  | (0.491) | (0.493) | (0.495) | (0.498) |
| Regional DG mobilization events (log) | -0.156 | -0.160 | -0.152 | -0.152 |
|  | (0.169) | (0.168) | (0.170) | (0.169) |
| lsdm\_any\_c\_no | 0.228 | 0.229 | 0.213 | 0.222 |
|  | (0.202) | (0.202) | (0.201) | (0.201) |
| size\_diff\_nsc | -0.200 | -0.185 | -0.194 | -0.181 |
|  | (0.594) | (0.599) | (0.592) | (0.596) |
| lstate\_control\_years | 0.170\*\* | 0.169\*\* | 0.169\*\* | 0.168\*\* |
|  | (0.063) | (0.063) | (0.063) | (0.062) |
| lunreg\_backlash\_no | 0.069\* | 0.069\* | 0.069\* | 0.069\* |
|  | (0.028) | (0.028) | (0.028) | (0.028) |
| Constant | -0.409 | -0.360 | -0.454 | -0.442 |
|  | (3.637) | (3.653) | (3.624) | (3.632) |
| Country-FE | yes | yes | yes | yes |
| Year-FE | yes | yes | yes | yes |
| Wald-Test Chisq |  |  |  |  |
| Joint sig. int. concession |  | 0.001\*\* |  |  |
| Joint sig. int. concession (group-based) |  |  |  | 0.01\*\* |
| Joint sig. int. concession (group-blind) |  |  |  | 0.93 |
| N | 38130 | 38130 | 38130 | 38130 |
| Log Likelihood | -23017.320 | -23016.250 | -23015.450 | -23011.890 |
| theta | 0.518\*\*\* (0.015) | 0.518\*\*\* (0.015) | 0.518\*\*\* (0.015) | 0.519\*\*\* (0.015) |
| AIC | 46378.640 | 46378.490 | 46376.890 | 46373.770 |
|  | | | | |
| † p<0.1; \* p<0.05; \*\* p<0.01; \*\*\* p<0.001; country-clustered SE's in parentheses; cubic terms for group-wise months without mobilization included but not reported. | | | | |
