# Supplementary material for: Ethnic Accommodation and the Backlash From Dominant Groups
Source: J Conflict Resolut. 2025 May 22;70(2-3):359–86. doi: 10.1177/00220027251343836 (PMC12782309; doi:10.1177/00220027251343836)
Supplement: Supplemental Material - Ethnic Accommodation and the Backlash From Dominant Groups [file sj-zip-3-jcr-10.1177_00220027251343836.zip › tables/results/app3.4_ac3.html]

**Ethnic accommodation and the number of mobilization events involving the dominant group [additional controls for dominant nationalists' influence].**

|  | | | | |
|  | **Model 1** | **Model 2** | **Model 3** | **Model 4** |
|  | | | | |
| Concession number | 0.133\* | 0.148 |  |  |
|  | (0.055) | (0.096) |  |  |
| Concession number x DN party |  | -0.027 |  |  |
|  |  | (0.116) |  |  |
| Concession number (group-based) |  |  | 0.245\* | 0.129 |
|  |  |  | (0.100) | (0.143) |
| Concession number (group-based) x DN party |  |  |  | 0.190 |
|  |  |  |  | (0.196) |
| Concession number (group-blind) |  |  | 0.029 | 0.164 |
|  |  |  | (0.118) | (0.142) |
| Concession number (group-blind) x DN party |  |  |  | -0.230 |
|  |  |  |  | (0.224) |
| DN party | 0.081 | 0.083 | 0.076 | 0.080 |
|  | (0.159) | (0.158) | (0.157) | (0.156) |
| DN party in government |  | 0.022 |  | 0.018 |
|  |  | (0.046) |  | (0.046) |
| Months to next election (log) | 0.553† | 0.596 | 0.540† | 0.551 |
|  | (0.301) | (0.412) | (0.300) | (0.400) |
| Recent subordinate group protest | -0.029 | -0.027 | -0.024 | -0.014 |
|  | (0.193) | (0.193) | (0.193) | (0.192) |
| Recent civil violence | 0.022 |  | 0.021 |  |
|  | (0.046) |  | (0.046) |  |
| Battle deaths (last 10y, log) | 0.046 | 0.046 | 0.048 | 0.048 |
|  | (0.094) | (0.094) | (0.095) | (0.095) |
| Democracy level | -0.071\* | -0.071\* | -0.072\* | -0.071\* |
|  | (0.032) | (0.032) | (0.032) | (0.032) |
| Abs. size (log) | 0.382\*\*\* | 0.382\*\*\* | 0.382\*\*\* | 0.383\*\*\* |
|  | (0.082) | (0.082) | (0.081) | (0.082) |
| GDP p.c. (log) | 0.147 | 0.147 | 0.145 | 0.146 |
|  | (0.123) | (0.123) | (0.122) | (0.122) |
| GDP growth | 0.068 | 0.068 | 0.069 | 0.071 |
|  | (0.071) | (0.072) | (0.071) | (0.072) |
| Regional DG mobilization events (log) | -0.382 | -0.382 | -0.368 | -0.381 |
|  | (0.329) | (0.329) | (0.334) | (0.328) |
| lsize\_abs | 0.229 | 0.229 | 0.229 | 0.235 |
|  | (0.182) | (0.182) | (0.182) | (0.180) |
| lgdppc | -0.232 | -0.232 | -0.225 | -0.223 |
|  | (0.302) | (0.302) | (0.300) | (0.299) |
| gdppc\_change | -0.887† | -0.887† | -0.908† | -0.919† |
|  | (0.496) | (0.496) | (0.499) | (0.500) |
| tt\_vdem2\_dem | 0.048 | 0.048 | 0.049 | 0.047 |
|  | (0.093) | (0.093) | (0.093) | (0.093) |
| month\_const\_shock3\_close3 | -0.243 | -0.268 | -0.257 | -0.265 |
|  | (0.234) | (0.293) | (0.234) | (0.292) |
| lunreg\_backlash\_no | 0.067\* | 0.067\* | 0.067\* | 0.067\* |
|  | (0.029) | (0.029) | (0.029) | (0.029) |
| Constant | 0.717 | 0.712 | 0.635 | 0.603 |
|  | (3.243) | (3.240) | (3.226) | (3.213) |
| Country-FE | yes | yes | yes | yes |
| Year-FE | yes | yes | yes | yes |
| Wald-Test Chisq |  |  |  |  |
| Joint sig. int. concession |  | 0.063† |  |  |
| Joint sig. int. concession (group-based) |  |  |  | 0.016\* |
| Joint sig. int. concession (group-blind) |  |  |  | 0.708 |
| N | 38130 | 38130 | 38130 | 38130 |
| Log Likelihood | -23029.260 | -23029.190 | -23027.210 | -23025.250 |
| theta | 0.514\*\*\* (0.014) | 0.514\*\*\* (0.014) | 0.514\*\*\* (0.015) | 0.515\*\*\* (0.015) |
| AIC | 46404.530 | 46406.370 | 46402.420 | 46402.500 |
|  | | | | |
| † p<0.1; \* p<0.05; \*\* p<0.01; \*\*\* p<0.001; country-clustered SE's in parentheses; cubic terms for group-wise months without mobilization included but not reported. | | | | |
