# Supplementary material for: Ethnic Accommodation and the Backlash From Dominant Groups
Source: J Conflict Resolut. 2025 May 22;70(2-3):359–86. doi: 10.1177/00220027251343836 (PMC12782309; doi:10.1177/00220027251343836)
Supplement: Supplemental Material - Ethnic Accommodation and the Backlash From Dominant Groups [file sj-zip-3-jcr-10.1177_00220027251343836.zip › tables/results/app3.5_cylevel.html]

**Ethnic accommodation and the number of mobilization events involving the dominant group [country year-level estimation].**

|  | | | | |
|  | **Model 1** | **Model 2** | **Model 3** | **Model 4** |
|  | | | | |
| Concession number | 0.136\*\* | 0.030 |  |  |
|  | (0.050) | (0.063) |  |  |
| Concession number x DN party |  | 0.179† |  |  |
|  |  | (0.098) |  |  |
| Concession number (group-based) |  |  | 0.231\* | -0.073 |
|  |  |  | (0.111) | (0.147) |
| Concession number (group-based) x DN party |  |  |  | 0.499\* |
|  |  |  |  | (0.212) |
| Concession number (group-blind) |  |  | 0.050 | 0.125 |
|  |  |  | (0.121) | (0.165) |
| Concession number (group-blind) x DN party |  |  |  | -0.108 |
|  |  |  |  | (0.235) |
| DN party | 0.085 | 0.048 | 0.085 | 0.050 |
|  | (0.180) | (0.179) | (0.179) | (0.177) |
| DN party in government | 0.159 | 0.169 | 0.159 | 0.167 |
|  | (0.109) | (0.111) | (0.109) | (0.110) |
| Months to next election (log) | 0.082 | 0.084 | 0.085 | 0.092 |
|  | (0.067) | (0.067) | (0.066) | (0.066) |
| Recent subordinate group protest | -0.987† | -0.981† | -0.971† | -0.992† |
|  | (0.508) | (0.513) | (0.514) | (0.512) |
| Recent civil violence | 0.455† | 0.460\* | 0.460\* | 0.479\* |
|  | (0.233) | (0.231) | (0.232) | (0.231) |
| Battle deaths (last 10y, log) | -0.134 | -0.138 | -0.123 | -0.122 |
|  | (0.305) | (0.305) | (0.302) | (0.301) |
| Democracy level | -1.777\*\* | -1.734\*\*\* | -1.789\*\* | -1.772\*\*\* |
|  | (0.541) | (0.526) | (0.547) | (0.534) |
| Abs. size (log) | 1.455 | 1.500 | 1.334 | 1.299 |
|  | (3.287) | (3.289) | (3.258) | (3.245) |
| Country-FE | yes | yes | yes | yes |
| Year-FE | yes | yes | yes | yes |
| Wald-Test Chisq |  |  |  |  |
| Joint sig. int. concession |  | 0.003\*\* |  |  |
| Joint sig. int. concession (group-based) |  |  |  | 0.003\*\* |
| Joint sig. int. concession (group-blind) |  |  |  | 0.923 |
| N | 3376 | 3376 | 3376 | 3376 |
| Log Likelihood | -7191.516 | -7189.258 | -7190.717 | -7186.379 |
| theta | 0.986\*\*\* (0.037) | 0.989\*\*\* (0.037) | 0.987\*\*\* (0.037) | 0.992\*\*\* (0.037) |
| AIC | 14705.030 | 14702.510 | 14705.430 | 14700.760 |
|  | | | | |
| † p<0.1; \* p<0.05; \*\* p<0.01; \*\*\* p<0.001; country-clustered SE's in parentheses; cubic terms for group-wise months without mobilization included but not reported. | | | | |
