# Supplementary material for: Ethnic Accommodation and the Backlash From Dominant Groups
Source: J Conflict Resolut. 2025 May 22;70(2-3):359–86. doi: 10.1177/00220027251343836 (PMC12782309; doi:10.1177/00220027251343836)
Supplement: Supplemental Material - Ethnic Accommodation and the Backlash From Dominant Groups [file sj-zip-3-jcr-10.1177_00220027251343836.zip › tables/results/app3.5_logit.html]

**Ethnic accommodation and the number of mobilization events involving the dominant group [binary dependent variable and logistic specification].**

|  | | | | |
|  | **Model 1** | **Model 2** | **Model 3** | **Model 4** |
|  | | | | |
| Concession number | 0.112\*\* | 0.114† |  |  |
|  | (0.042) | (0.062) |  |  |
| Concession number x DN party |  | -0.005 |  |  |
|  |  | (0.086) |  |  |
| Concession number (group-based) |  |  | 0.225\* | 0.078 |
|  |  |  | (0.090) | (0.131) |
| Concession number (group-based) x DN party |  |  |  | 0.262 |
|  |  |  |  | (0.183) |
| Concession number (group-blind) |  |  | 0.0004 | 0.149 |
|  |  |  | (0.089) | (0.118) |
| Concession number (group-blind) x DN party |  |  |  | -0.269 |
|  |  |  |  | (0.165) |
| DN party | 0.138 | 0.139 | 0.140 | 0.142 |
|  | (0.144) | (0.142) | (0.143) | (0.141) |
| DN party in government | 0.110 | 0.110 | 0.111 | 0.111 |
|  | (0.091) | (0.091) | (0.091) | (0.091) |
| Months to next election (log) | -0.019 | -0.019 | -0.020 | -0.020 |
|  | (0.021) | (0.021) | (0.021) | (0.021) |
| Recent subordinate group protest | 0.366\*\*\* | 0.366\*\*\* | 0.365\*\*\* | 0.366\*\*\* |
|  | (0.067) | (0.067) | (0.067) | (0.067) |
| Recent civil violence | 0.092 | 0.092 | 0.089 | 0.090 |
|  | (0.116) | (0.116) | (0.115) | (0.114) |
| Battle deaths (last 10y, log) | 0.055 | 0.055 | 0.055 | 0.056 |
|  | (0.055) | (0.055) | (0.054) | (0.055) |
| Democracy level | -0.200 | -0.199 | -0.185 | -0.189 |
|  | (0.329) | (0.329) | (0.330) | (0.324) |
| Abs. size (log) | -0.025 | -0.025 | -0.023 | -0.016 |
|  | (0.162) | (0.162) | (0.162) | (0.162) |
| GDP p.c. (log) | -0.113 | -0.113 | -0.107 | -0.107 |
|  | (0.246) | (0.246) | (0.246) | (0.245) |
| GDP growth | -0.823\* | -0.823\* | -0.827\* | -0.839\* |
|  | (0.408) | (0.408) | (0.411) | (0.412) |
| Regional DG mobilization events (log) | 0.082\*\* | 0.082\*\* | 0.082\*\* | 0.083\*\* |
|  | (0.027) | (0.027) | (0.027) | (0.027) |
| Constant | -0.368 | -0.369 | -0.450 | -0.469 |
|  | (2.621) | (2.618) | (2.620) | (2.610) |
| Country-FE | yes | yes | yes | yes |
| Year-FE | yes | yes | yes | yes |
| Wald-Test Chisq |  |  |  |  |
| Joint sig. int. concession |  | 0.057† |  |  |
| Joint sig. int. concession (group-based) |  |  |  | 0.005\*\* |
| Joint sig. int. concession (group-blind) |  |  |  | 0.302 |
| N | 38130 | 38130 | 38130 | 38130 |
| Log Likelihood | -15259.610 | -15259.600 | -15257.900 | -15255.560 |
| AIC | 30855.220 | 30857.210 | 30853.800 | 30853.130 |
|  | | | | |
| † p<0.1; \* p<0.05; \*\* p<0.01; \*\*\* p<0.001; country-clustered SE's in parentheses; cubic terms for group-wise months without mobilization included but not reported. | | | | |
