# Supplementary material for: Ethnic Accommodation and the Backlash From Dominant Groups
Source: J Conflict Resolut. 2025 May 22;70(2-3):359–86. doi: 10.1177/00220027251343836 (PMC12782309; doi:10.1177/00220027251343836)
Supplement: Supplemental Material - Ethnic Accommodation and the Backlash From Dominant Groups [file sj-zip-3-jcr-10.1177_00220027251343836.zip › tables/results/app3.5_ols.html]

**Ethnic accommodation and the number of mobilization events involving the dominant group [OLS model].**

|  | | | | |
|  | **Model 1** | **Model 2** | **Model 3** | **Model 4** |
|  | | | | |
| Concession number | 0.079\* | 0.040 |  |  |
|  | (0.036) | (0.062) |  |  |
| Concession number x DN party |  | 0.071 |  |  |
|  |  | (0.085) |  |  |
| Concession number (group-based) |  |  | 0.171\* | 0.055 |
|  |  |  | (0.069) | (0.065) |
| Concession number (group-based) x DN party |  |  |  | 0.207 |
|  |  |  |  | (0.129) |
| Concession number (group-blind) |  |  | -0.004 | 0.028 |
|  |  |  | (0.070) | (0.084) |
| Concession number (group-blind) x DN party |  |  |  | -0.058 |
|  |  |  |  | (0.141) |
| DN party | 0.068 | 0.059 | 0.068 | 0.062 |
|  | (0.099) | (0.098) | (0.099) | (0.098) |
| DN party in government | -0.009 | -0.006 | -0.010 | -0.007 |
|  | (0.041) | (0.042) | (0.041) | (0.041) |
| Months to next election (log) | -0.018\* | -0.019\* | -0.019\* | -0.019\* |
|  | (0.008) | (0.008) | (0.008) | (0.008) |
| Recent subordinate group protest | 0.343\*\* | 0.344\*\* | 0.341\*\* | 0.343\*\* |
|  | (0.129) | (0.129) | (0.129) | (0.129) |
| Recent civil violence | 0.031 | 0.031 | 0.029 | 0.030 |
|  | (0.079) | (0.078) | (0.078) | (0.078) |
| Battle deaths (last 10y, log) | 0.100 | 0.101 | 0.100 | 0.102 |
|  | (0.095) | (0.096) | (0.095) | (0.095) |
| Democracy level | -0.040 | -0.044 | -0.030 | -0.034 |
|  | (0.249) | (0.247) | (0.251) | (0.248) |
| Abs. size (log) | 0.262 | 0.263 | 0.264 | 0.268 |
|  | (0.222) | (0.222) | (0.222) | (0.222) |
| GDP p.c. (log) | -0.091 | -0.092 | -0.088 | -0.088 |
|  | (0.180) | (0.180) | (0.179) | (0.179) |
| GDP growth | -0.367 | -0.368 | -0.373 | -0.377 |
|  | (0.240) | (0.241) | (0.242) | (0.243) |
| Regional DG mobilization events (log) | 0.010 | 0.010 | 0.010 | 0.010 |
|  | (0.014) | (0.014) | (0.014) | (0.014) |
| Constant | 0.499 | 0.509 | 0.454 | 0.450 |
|  | (2.279) | (2.274) | (2.278) | (2.269) |
| Country-FE | yes | yes | yes | yes |
| Year-FE | yes | yes | yes | yes |
| Wald-Test Chisq |  |  |  |  |
| Joint sig. int. concession |  | 0.025\* |  |  |
| Joint sig. int. concession (group-based) |  |  |  | 0.019\* |
| Joint sig. int. concession (group-blind) |  |  |  | 0.782 |
| N | 38130 | 38130 | 38130 | 38130 |
| Log Likelihood | -57437.140 | -57434.060 | -57431.470 | -57425.460 |
| AIC | 115210.300 | 115206.100 | 115200.900 | 115192.900 |
|  | | | | |
| † p<0.1; \* p<0.05; \*\* p<0.01; \*\*\* p<0.001; country-clustered SE's in parentheses; cubic terms for group-wise months without mobilization included but not reported. | | | | |
