# Supplementary material for: Ethnic Accommodation and the Backlash From Dominant Groups
Source: J Conflict Resolut. 2025 May 22;70(2-3):359–86. doi: 10.1177/00220027251343836 (PMC12782309; doi:10.1177/00220027251343836)
Supplement: Supplemental Material - Ethnic Accommodation and the Backlash From Dominant Groups [file sj-zip-3-jcr-10.1177_00220027251343836.zip › tables/results/app4_indresults.html]

**Individual-level results.**

|  | | | | | | |
|  | **Model 1** | **Model 2** | **Model 3** | **Model 4** | **Model 5** | **Model 6** |
|  | | | | | | |
| conc\_const\_sum1 | -0.068 | -0.114 | -0.145 |  |  |  |
|  | (0.111) | (0.111) | (0.112) |  |  |  |
| conc\_const\_sum1:authoritarian |  | 0.210\*\*\* |  |  |  |  |
|  |  | (0.033) |  |  |  |  |
| conc\_const\_sum1:rightwing |  |  | 0.298\*\*\* |  |  |  |
|  |  |  | (0.027) |  |  |  |
| conc\_symbolic\_const\_sum1 |  |  |  | 0.342\*\* | 0.302\* | 0.263\* |
|  |  |  |  | (0.125) | (0.124) | (0.125) |
| conc\_symbolic\_const\_sum1:authoritarian |  |  |  |  | 0.190\*\*\* |  |
|  |  |  |  |  | (0.032) |  |
| conc\_symbolic\_const\_sum1:rightwing |  |  |  |  |  | 0.270\*\*\* |
|  |  |  |  |  |  | (0.028) |
| conc\_nonsymbolic\_const\_sum1 |  |  |  | -0.282\* | -0.317\* | -0.288\* |
|  |  |  |  | (0.142) | (0.142) | (0.143) |
| authoritarian:conc\_nonsymbolic\_const\_sum1 |  |  |  |  | 0.077\* |  |
|  |  |  |  |  | (0.033) |  |
| rightwing:conc\_nonsymbolic\_const\_sum1 |  |  |  |  |  | 0.050† |
|  |  |  |  |  |  | (0.028) |
| ld10\_bdead\_allc | 0.049 | 0.049 | 0.045 | 0.066 | 0.064 | 0.062 |
|  | (0.080) | (0.080) | (0.080) | (0.079) | (0.078) | (0.079) |
| mnm\_party\_exists | -0.110 | -0.108 | -0.109 | -0.139 | -0.144 | -0.135 |
|  | (0.149) | (0.149) | (0.149) | (0.145) | (0.145) | (0.145) |
| mnm\_partygov | -0.093 | -0.099 | -0.090 | -0.075 | -0.082 | -0.073 |
|  | (0.081) | (0.081) | (0.082) | (0.081) | (0.080) | (0.081) |
| vdem\_libdem | 0.692 | 0.691 | 0.661 | 0.965\* | 0.944\* | 0.948\* |
|  | (0.437) | (0.436) | (0.438) | (0.438) | (0.436) | (0.437) |
| lsize\_abs | -1.089† | -1.088† | -1.072† | -1.191† | -1.200\* | -1.184† |
|  | (0.621) | (0.620) | (0.622) | (0.608) | (0.605) | (0.608) |
| lgdppc | -1.171\*\*\* | -1.166\*\*\* | -1.167\*\*\* | -1.060\*\*\* | -1.054\*\*\* | -1.056\*\*\* |
|  | (0.233) | (0.233) | (0.234) | (0.230) | (0.229) | (0.230) |
| gdppc\_change | -1.781\* | -1.738\* | -1.827\* | -1.682\* | -1.604\* | -1.746\* |
|  | (0.798) | (0.797) | (0.800) | (0.778) | (0.775) | (0.778) |
| ltt\_nextelec | 0.093\*\* | 0.094\*\* | 0.094\*\* | 0.093\*\* | 0.094\*\* | 0.095\*\* |
|  | (0.030) | (0.030) | (0.030) | (0.030) | (0.030) | (0.030) |
| month\_protest2\_nsc\_forward3\_close3 | -0.227\*\* | -0.228\*\* | -0.230\*\* | -0.220\*\* | -0.221\*\* | -0.221\*\* |
|  | (0.083) | (0.083) | (0.084) | (0.083) | (0.082) | (0.083) |
| month\_viol\_civil\_nsc\_forward3\_close3 | 0.021 | 0.021 | 0.017 | -0.020 | -0.027 | -0.020 |
|  | (0.135) | (0.135) | (0.136) | (0.134) | (0.133) | (0.134) |
| age | -0.018\*\*\* | -0.018\*\*\* | -0.018\*\*\* | -0.018\*\*\* | -0.018\*\*\* | -0.018\*\*\* |
|  | (0.0003) | (0.0003) | (0.0003) | (0.0003) | (0.0003) | (0.0003) |
| gender | -0.302\*\*\* | -0.303\*\*\* | -0.304\*\*\* | -0.302\*\*\* | -0.302\*\*\* | -0.303\*\*\* |
|  | (0.011) | (0.011) | (0.011) | (0.011) | (0.011) | (0.011) |
| educ\_high | 0.570\*\*\* | 0.569\*\*\* | 0.568\*\*\* | 0.570\*\*\* | 0.569\*\*\* | 0.570\*\*\* |
|  | (0.015) | (0.015) | (0.015) | (0.015) | (0.015) | (0.015) |
| interest\_pol\_d | 0.868\*\*\* | 0.868\*\*\* | 0.866\*\*\* | 0.868\*\*\* | 0.868\*\*\* | 0.867\*\*\* |
|  | (0.011) | (0.011) | (0.011) | (0.011) | (0.011) | (0.011) |
| rightwing | -0.067\*\*\* | -0.067\*\*\* | -0.281\*\*\* | -0.067\*\*\* | -0.066\*\*\* | -0.245\*\*\* |
|  | (0.012) | (0.012) | (0.023) | (0.012) | (0.012) | (0.021) |
| authoritarian | -0.184\*\*\* | -0.343\*\*\* | -0.184\*\*\* | -0.183\*\*\* | -0.352\*\*\* | -0.183\*\*\* |
|  | (0.014) | (0.029) | (0.014) | (0.014) | (0.026) | (0.014) |
| Constant | 13.667\*\*\* | 13.616\*\*\* | 13.637\*\*\* | 12.964\*\*\* | 12.972\*\*\* | 12.937\*\*\* |
|  | (3.650) | (3.644) | (3.659) | (3.581) | (3.565) | (3.579) |
| Country-FE | yes | yes | yes | yes | yes | yes |
| Year-FE | yes | yes | yes | yes | yes | yes |
| Survey wave-FE | yes | yes | yes | yes | yes | yes |
| N | 175635 | 175635 | 175635 | 175635 | 175635 | 175635 |
| Log Likelihood | -105058.300 | -105037.600 | -104997.300 | -105054.300 | -105021.000 | -104980.400 |
| AIC | 210348.700 | 210309.300 | 210228.500 | 210342.600 | 210280.100 | 210198.800 |
| BIC | 211517.500 | 211488.200 | 211407.400 | 211521.600 | 211479.200 | 211397.800 |
|  | | | | | | |
| \*\*\*p < .01; \*\*p < .05; \*p < .1 | | | | | | |
