# Supplementary material for: Ethnic Accommodation and the Backlash From Dominant Groups
Source: J Conflict Resolut. 2025 May 22;70(2-3):359–86. doi: 10.1177/00220027251343836 (PMC12782309; doi:10.1177/00220027251343836)
Supplement: Supplemental Material - Ethnic Accommodation and the Backlash From Dominant Groups [file sj-zip-3-jcr-10.1177_00220027251343836.zip › tables/descriptives/descriptive_stats.html]

**Descriptive statistics.**

|  | | | | | |
| Statistic | N | Mean | St. Dev. | Min | Max |
|  | | | | | |
| No. DG mobilization events | 38130 | 0.323 | 1.159 | 0 | 56 |
| No. DG anti-government protests | 38130 | 0.278 | 0.890 | 0 | 31 |
| No. DG violent incidents | 38130 | 0.046 | 0.718 | 0 | 53 |
| Concession number | 38130 | 0.081 | 0.404 | 0 | 6 |
| Concession number (group-based) | 38130 | 0.035 | 0.220 | 0 | 3 |
| Concession number (group-blind) | 38130 | 0.046 | 0.240 | 0 | 3 |
| Months to next election (log) | 38130 | 2.792 | 0.990 | 0.000 | 5.257 |
| Recent subordinate group protest | 38130 | 0.060 | 0.238 | 0 | 1 |
| Recent civil violence | 38130 | 0.143 | 0.350 | 0 | 1 |
| Battle deaths (last 10y, log) | 38130 | 0.448 | 0.922 | 0.000 | 5.141 |
| DN party | 38130 | 0.453 | 0.498 | 0 | 1 |
| DN party in government | 38130 | 0.295 | 0.456 | 0 | 1 |
| Democracy level | 38130 | 0.413 | 0.248 | 0.017 | 0.861 |
| Abs. size (log) | 38130 | 2.108 | 1.270 | 0.025 | 5.867 |
| GDP p.c. (log) | 38130 | 8.818 | 1.136 | 5.870 | 11.357 |
| GDP growth | 38130 | 0.017 | 0.072 | -1.647 | 0.584 |
| Regional DG mobilization events (log) | 38130 | 1.036 | 0.766 | 0.000 | 4.043 |
| Months without DG mobilization | 38130 | 15.409 | 27.970 | 1 | 339 |
| Year | 38130 | 2004.522 | 8.218 | 1990 | 2018 |
|  | | | | | |
