# Supplementary material for: Ethnic Accommodation and the Backlash From Dominant Groups
Source: J Conflict Resolut. 2025 May 22;70(2-3):359–86. doi: 10.1177/00220027251343836 (PMC12782309; doi:10.1177/00220027251343836)
Supplement: Supplemental Material - Ethnic Accommodation and the Backlash From Dominant Groups [file sj-zip-3-jcr-10.1177_00220027251343836.zip › tables/results/main.html]

**Ethnic accommodation and the number of mobilization events involving the dominant group.**

|  | | | | |
|  | **Model 1** | **Model 2** | **Model 3** | **Model 4** |
|  | | | | |
| Concession number | 0.147\*\*\* | 0.092 |  |  |
|  | (0.039) | (0.060) |  |  |
| Concession number x DN party |  | 0.095 |  |  |
|  |  | (0.081) |  |  |
| Concession number (group-based) |  |  | 0.267\* | 0.059 |
|  |  |  | (0.107) | (0.119) |
| Concession number (group-based) x DN party |  |  |  | 0.340† |
|  |  |  |  | (0.188) |
| Concession number (group-blind) |  |  | 0.028 | 0.123 |
|  |  |  | (0.107) | (0.129) |
| Concession number (group-blind) x DN party |  |  |  | -0.150 |
|  |  |  |  | (0.205) |
| DN party | 0.082 | 0.070 | 0.080 | 0.069 |
|  | (0.166) | (0.165) | (0.165) | (0.163) |
| DN party in government | 0.041 | 0.046 | 0.043 | 0.049 |
|  | (0.093) | (0.093) | (0.094) | (0.094) |
| Months to next election (log) | -0.059\*\* | -0.060\*\* | -0.061\*\* | -0.062\*\* |
|  | (0.023) | (0.023) | (0.023) | (0.023) |
| Recent subordinate group protest | 0.385\*\*\* | 0.386\*\*\* | 0.385\*\*\* | 0.387\*\*\* |
|  | (0.082) | (0.082) | (0.082) | (0.082) |
| Recent civil violence | 0.145 | 0.144 | 0.144 | 0.143 |
|  | (0.122) | (0.122) | (0.121) | (0.120) |
| Battle deaths (last 10y, log) | 0.065 | 0.066 | 0.066 | 0.069 |
|  | (0.072) | (0.072) | (0.072) | (0.071) |
| Democracy level | -0.400 | -0.404 | -0.383 | -0.401 |
|  | (0.324) | (0.327) | (0.330) | (0.326) |
| Abs. size (log) | 0.210 | 0.212 | 0.210 | 0.219 |
|  | (0.183) | (0.182) | (0.182) | (0.179) |
| GDP p.c. (log) | -0.222 | -0.225 | -0.214 | -0.215 |
|  | (0.298) | (0.299) | (0.297) | (0.297) |
| GDP growth | -0.941† | -0.931† | -0.961† | -0.960† |
|  | (0.502) | (0.503) | (0.506) | (0.508) |
| Regional DG mobilization events (log) | 0.067\* | 0.067\* | 0.067\* | 0.067\* |
|  | (0.029) | (0.029) | (0.029) | (0.029) |
| Constant | 0.656 | 0.687 | 0.566 | 0.574 |
|  | (3.248) | (3.253) | (3.231) | (3.226) |
| Country-FE | yes | yes | yes | yes |
| Year-FE | yes | yes | yes | yes |
| Wald-Test Chisq |  |  |  |  |
| Joint sig. int. concession |  | 0.001\*\* |  |  |
| Joint sig. int. concession (group-based) |  |  |  | 0.007\*\* |
| Joint sig. int. concession (group-blind) |  |  |  | 0.86 |
| N | 38130 | 38130 | 38130 | 38130 |
| Log Likelihood | -23038.070 | -23036.680 | -23035.530 | -23031.730 |
| theta | 0.513\*\*\* (0.014) | 0.513\*\*\* (0.014) | 0.513\*\*\* (0.014) | 0.514\*\*\* (0.014) |
| AIC | 46412.130 | 46411.360 | 46409.060 | 46405.460 |
|  | | | | |
| † p<0.1; \* p<0.05; \*\* p<0.01; \*\*\* p<0.001; country-clustered SE's in parentheses; cubic terms for group-wise months without mobilization included but not reported. | | | | |
